# Heritable transgene-free genome editing in plants by grafting of wild-type shoots to transgenic donor rootstocks

**DOI:** 10.1038/s41587-022-01585-8

**Published:** 2023-01-02

**Authors:** Lei Yang, Frank Machin, Shuangfeng Wang, Eleftheria Saplaoura, Friedrich Kragler

**Affiliations:** Max Planck Institute of Molecular Plant Physiology, Potsdam, Germany

**Keywords:** Molecular engineering in plants, Plant breeding, Plant breeding

## Abstract

Generation of stable gene-edited plant lines using clustered regularly interspaced short palindromic repeats (CRISPR)–CRISPR-associated protein 9 (Cas9) requires a lengthy process of outcrossing to eliminate CRISPR–Cas9-associated sequences and produce transgene-free lines. We have addressed this issue by designing fusions of *Cas9* and guide RNA transcripts to tRNA-like sequence motifs that move RNAs from transgenic rootstocks to grafted wild-type shoots (scions) and achieve heritable gene editing, as demonstrated in wild-type *Arabidopsis thaliana* and *Brassica rapa*. The graft-mobile gene editing system enables the production of transgene-free offspring in one generation without the need for transgene elimination, culture recovery and selection, or use of viral editing vectors. We anticipate that using graft-mobile editing systems for transgene-free plant production may be applied to a wide range of breeding programs and crop plants.

## Abstract (web summary)

Gene-edited plants free of CRISPR-associated sequences are generated by grafting.

## Main

Programmed by guide RNA (gRNA) sequences, the clustered regularly interspaced short palindromic repeats (CRISPR)-associated protein 9 (Cas9) nuclease creates DNA double-strand breaks at specific genomic sequences, resulting in edited sequences^1,2^. In plants, to obtain transgene-free mutants and to stabilize the genomes, both Cas9 enzyme and gRNA must be either introduced transiently or outcrossed from a mutant progeny^3^. For example, functional gRNA and Cas9 complexes can be ectopically introduced into protoplasted cells or embryos from which transgene-free edited plants have to be regenerated and selected. These approaches require time-consuming plant regeneration and selection steps, or expensive reagents and special equipment^4^. Given that most plant species are not easily accessible to transformation or have long generation times we sought to design *Cas9* and gRNA transcripts that are transported from transgenic roots to wild-type shoots (scions). On such grafted wild-type scions, gene-edited (that is, mutant) seeds might be created by gRNA and *Cas9* transcripts transported from a transgenic rootstock. Transcript mobility can be introduced by tRNA-like sequence (TLS) motifs and variants thereof shown to license transport of protein encoding transcripts over graft junctions^5^ or to parasitic *Cuscuta* plants feeding on host plants^6^. We postulated that the addition of TLS motifs to *Cas9* and gRNA transcripts will result in their root-to-shoot movement and cause editing in recipient-grafted wild-type tissues. Consequently, transgene-free scions grafted on transgenic mobile *Cas9*/gRNA expressing rootstocks should produce seeds with edited genomes.

## Results

**Fig. 1:**
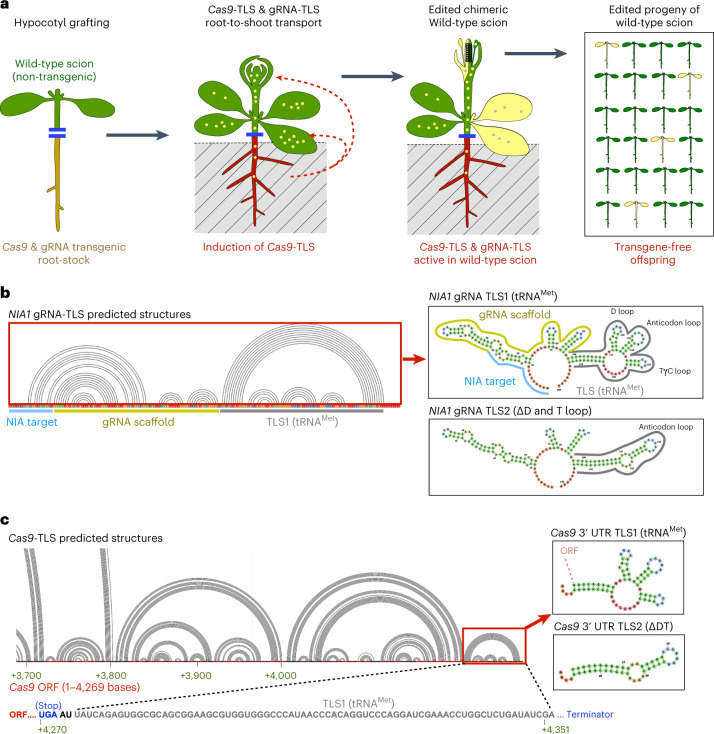
Conceptional overview of the applied transgene-free genome editing methods via mobile CRISPR–Cas9. **a**, Scheme of CRISPR–Cas9-mediated transgene-free gene editing by grafting. **b**,**c**, Predicted folding structures of the *gNIA1*-TLS and *Cas9*-TLS RNAs according to co-transcriptional folding. Red boxes indicate the two types of TLS motifs TLS1 (tRNA^Met^) and TLS2 (tRNA^Met ΔDT^) used in the study.

To test whether *Cas9* transcript and gRNA can be delivered from donor rootstocks to wild-type scions (Fig. 1a), we created two gRNAs introducing a genomic deletion in the *NITRATE REDUCTASE1* (*NIA1*, AT1G77760) gene. NIA1 is one of two *Arabidopsis* nitrate reductase enzymes converting nitrate (NO_3_) to ammonium (NH_4_) and on NH_4_-deficient medium *nia1* mutants develop a clearly visible phenotype with smaller plants that become chlorotic over time^7,8^, which should allow us to identify *nia1* mutants in progeny plants by phenotype. We generated *Arabidopsis thaliana* lines expressing zCas9^9^ from an estradiol-inducible promoter and two *NIA1*-targeting gRNAs (*gNIA1)* driven by the constitutive Pol-III promoters *U6-26* and *U6-29*^10^. The two *NIA1*-targeting gRNAs are designed to create double genome edits (deletions) resulting in the deletion of approximately 1,000 base pairs (bp) of the *NIA1* gene sequence creating a *nia1* knock out. Additionally, we constructed gRNAs targeting a transgenic *35S*_*promoter*_*::Venus::35S*_*terminator*_*::BASTA* construct. The two *gRNAs* are designed to introduce a deletion removing the Venus and 35S terminator sequences resulting in BASTA-resistant plants (Supplementary Data 1).The *Cas9* and the two *gNIA1* transcripts were either fused to TLS1 (tRNA^Met^ sequence) or TLS2 (tRNA^Met-ΔDT^ sequence, lacking the D and T loop)^5^ and a short poly-A tail was added to the 3′ end of each *gNIA1-TLS* (Fig. 1a,b and Supplementary Data 1). Co-fold RNA structure predictions^11^ confirmed that folding of the TLS motif was unlikely to be affected in the gRNA or Cas9 fusion constructs (Fig. 1b,c). Also, addition of related TLS motifs to gRNAs was shown not to interfere with the function of gRNAs^12,13^.

## *Cas9*/gRNA-TLS fusions move from root to shoot in grafted *Arabidopsis*

**Fig. 2:**
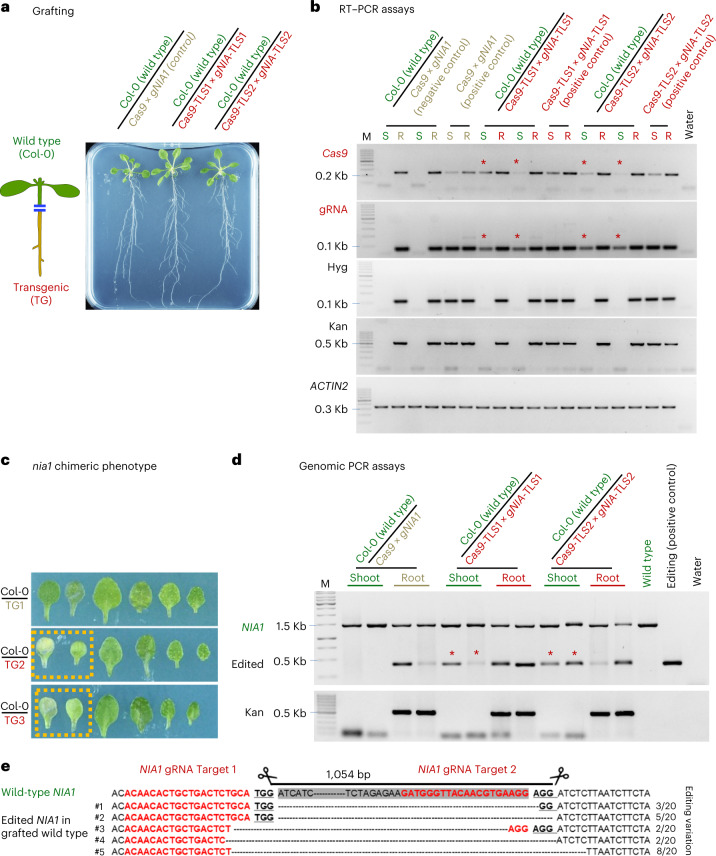
*Cas9* and *gNIA1*-TLS fusions move from root to shoot inducing *NIA1* edits in juvenile *Arabidopsis*. **a**, Three weeks after grafting shoot (S) and root (R) samples were harvested from plants cultured on 5 μM estradiol and 0% sucrose 0.5 MS medium. **b**, RT–PCR detection (45 PCR cycles) of *Cas9* and *gNIA1* transcripts (each sample a pool of 4–6 grafted plants) in transgenic root and grafted wild-type scion samples. Stars indicate presence of *Cas9*-TLS and *gNIA1*-TLS amplicons in Col-0 wild-type. M indicates molecular weight markers. RT–PCR of kanamycin (Kan) and hygromycin (Hyg) transcripts were used as contamination controls. Note that transcript absence was confirmed using 50 PCR cycles. **c**, Chlorotic leaf phenotype of *nia1* chimeric mutant in juvenile grafted plants. Dashed boxes indicate chlorotic phenotype detected in wild-type leaves. TG1, *Cas9* × *gNIA1* (control); TG2, *Cas9*-TLS1 × *gNIA1*-TLS1; and TG3, *Cas9*-TLS2 × *gNIA1*-TLS2. **d**, Genomic PCR assays to detect edited *NIA1* fragment with a deletion. PCR amplicons marked by stars indicate CRISPR–Cas9-induced mutations in wild-type tissue. Four independent replicates (4–6 pooled root or shoot samples) per graft combination were analyzed. Expected wild-type *NIA1* amplicon is 1,469 bp and edited *NIA1* is approximately 430 bp. **e**, Edits induced by mobile CRISPR–Cas9 confirmed by Sanger sequencing. Red bases indicate g*NIA1* target 1 and g*NIA1* target 2 sequences. Bold and underlined bases indicate the two protospacer-adjacent motif sites of the gRNA. Source data

We first tested whether *Cas9* and/or *gNIA1* transcripts were root-to-shoot mobile without a TLS by hypocotyl grafting *Cas9* × *gNIA1* rootstocks with wild-type (Col-0) scions. Three weeks after grafting, shoot and root samples were harvested separately (*n* = 4, samples were pools of 4–6 independent grafted plants) (Fig. 2a). We did not detect *Cas9* or gRNA in these wild-type scion samples, though both were detected in the rootstock samples, indicating that *Cas9* mRNA and gRNA transcripts targeting *NIA1* are not root-to-shoot mobile.

Conversely, in a similar experiment with rootstocks expressing *Cas9*-TLS1 and *gNIA1*-TLS1 or *Cas9*-TLS2 and *gNIA1*-TLS2, we detected the *Cas9*-TLS (four replicates for each TLS1 and TLS2 fusion) and the *gNIA1*-TLS (four replicates for each TLS1 and TLS2 fusion) transcripts in grafted transgene-free wild-type shoots (Fig. 2b). This finding confirmed that adding a TLS motif is sufficient to promote long-distance mobility of the *Cas9* and gRNA transcripts. As a control for tissue contamination and specificity of TLS-mediated transport, we used the hygromycin and kanamycin resistance genes expressed by transgenic roots. Both resistance genes were not detected in the wild-type shoot samples indicating that no contamination occurred during harvest and that both transcripts are not root-to-shoot transmissible (Fig. 2b).

Even though the *Cas9*-TLS and the *gNIA1*-TLS transcripts were moving from root to shoot, low transcript abundance or lack of *Cas9*-TLS translation in distal tissues might result in insufficient or no editing activity. Therefore, we asked whether gene deletion edits are detectable in root and shoot tissue samples of grafted plants. A chlorotic *nia1* mutant phenotype was detected in some leaves formed on grafted scions of plants grown on NH_4_-deficient medium 14 days after grafting (Fig. 2c), indicating that the mobile transcripts were functional in recipient wild-type tissues. This was observed in 20 out of 28 of Col-0/*Cas9*-TLS1 *×* *gNIA1*-TLS1 grafted plants and in 26 out 30 of Col-0/*Cas9*-TLS2 × *gNIA1*-TLS2 grafted plants, but never observed in the 20 tested Col-0/*Cas9* *×* *gNIA1* grafted plants. In line, *NIA1* deletion edits could be detected in all grafted transgenic roots (Fig. 2d) and in all tested wild-type scions grafted onto the mobile transcripts producing *Cas9*-TLS1 × *gNIA1*-TLS1 (*n* = 4) and *Cas9*-TLS2 × *gNIA1*-TLS2 (*n* = 4) rootstocks (Fig. 2d,e). Serving as contamination controls, we also probed for the presence of kanamycin resistance gene, which was not detectable in genomic PCR assays performed on wild-type scion samples (Fig. 2b,d). These results also show that the root-produced mobile *Cas9*-TLS transcript was translated into a functional protein in recipient tissues. Further, this indicates that both *Cas9*-TLS and *gNIA1*-TLS are transported in sufficient amounts for effective genome editing of the *NIA1* gene in grafted non-transgenic wild-type scions in the seedling stage.

## Mobile *Cas9*/gRNA produces heritable gene edits

**Fig. 3:**
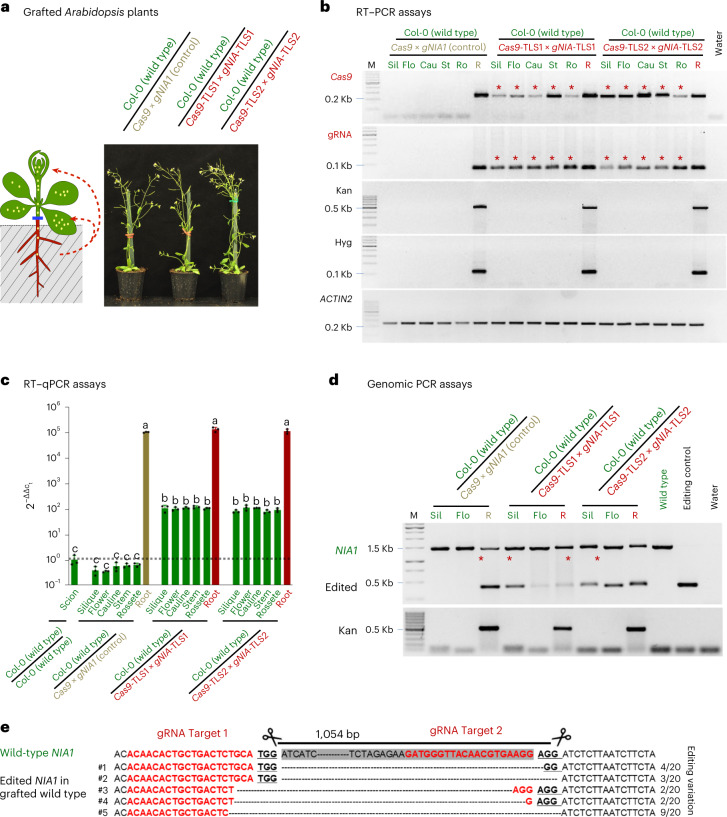
*Cas9*- and g*NIA1*-TLS fusion constructs are functional and mobile in adult plants. **a**, Appearance of grafted plants (43 days after grafting) grown on soil and treated with 5 μM estradiol. **b**, RT–PCR detection (45 PCR cycles) of *Cas9* and *gNIA1* transcripts in rootstock (R) and grafted wild-type tissues samples from silique (Sil), flower (Flo), cauline leaf (Cau), stem (St), and rosette (Ro). Four independent replicates (each sample a pool of three grafted plants) were analyzed. Stars indicate RT–PCR *Cas9*-TLS and *gNIA1*-TLS amplicons detected in wild-type scion samples. Kan and Hyg transcripts serve as contamination controls. Note that transcript absence was confirmed by 50 PCR cycles. **c**, RT–qPCR detection of mobile *Cas9*-TLS transcripts in scion tissues and in grafted rootstock (Root). *y*-axis, mean $2^{-\Delta\Delta c_t}$ values; log_10_ scale. Each value represents the mean of three independent biological replicates presented as black dots on the bar. Note that every $2^{-\Delta\Delta c_t}$ data point below the dashed line represents technical background as measured with wild-type scion samples grafted onto wild-type rootstocks with no *Cas9* transcript present. Significance was calculated using Student’s *t*-test (two tails); *P* values indicated by a, b, and c: a,b = 8.31567 × 10^−22^; b,c = 1.16396 × 10^−22^; a,c = 1.90267 × 10^−11^. **d**, Genomic PCR to detect *NIA1* edited fragments in siliques and flowers. Samples were from the same plant material as analyzed in **c**. **e**, Mobile CRISPR*–Cas9-*induced genome edits confirmed by Sanger sequencing. Source data

**Extended Data Fig. 1:**
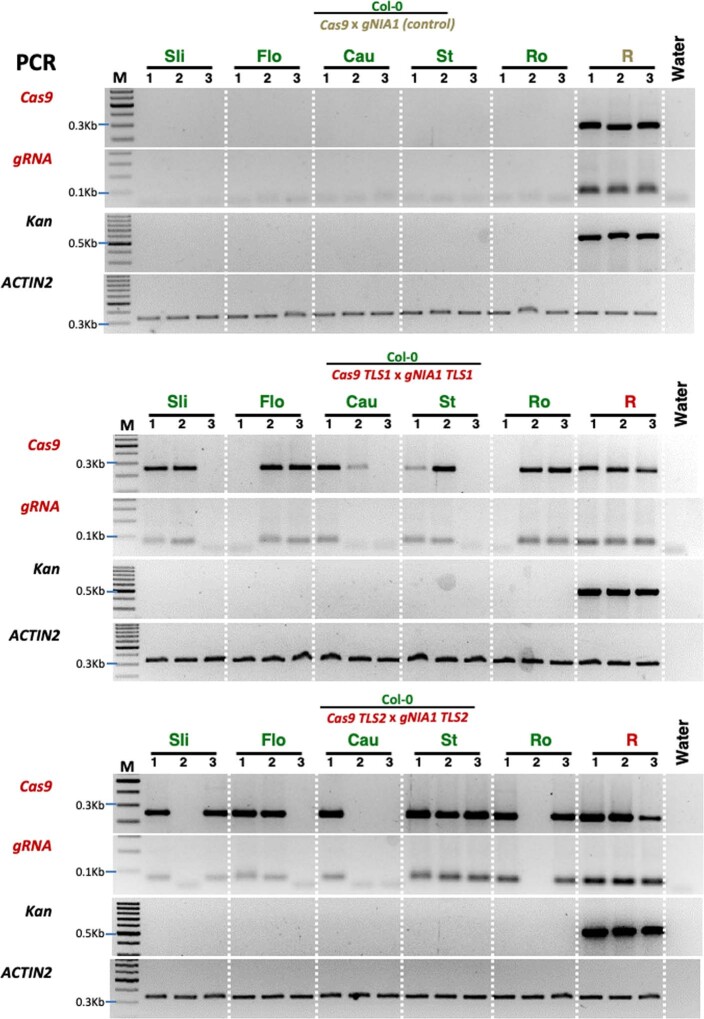
*Cas9* and g*NIA1* transcript presence in grafted wild-type scions. Tissue samples from transgenic roots (R) and wild-type silique (Sil), flower (Flo), cauline leaf (Cau), stem (St), rosette (Ro) of grafted plants were pooled for RT-PCR detection of *Cas9-TLS* and *gNIA1-TLS* fusion transcripts. For each graft combination three independent replicates each representing a pool of three grafted plants are shown which were tested by RT-PCR (45 PCR cycles) for the presence of fusion transcripts. *Kanamycin* (*Kan*) transcript expressed only in grafted transgenic rootstocks was used as contamination control in RT-PCR assays. Note that transcript absence of *Kan* was additionally confirmed by 50 PCR cycles. M: indicates molecular weight markers. Source data

**Extended Data Fig. 2:**
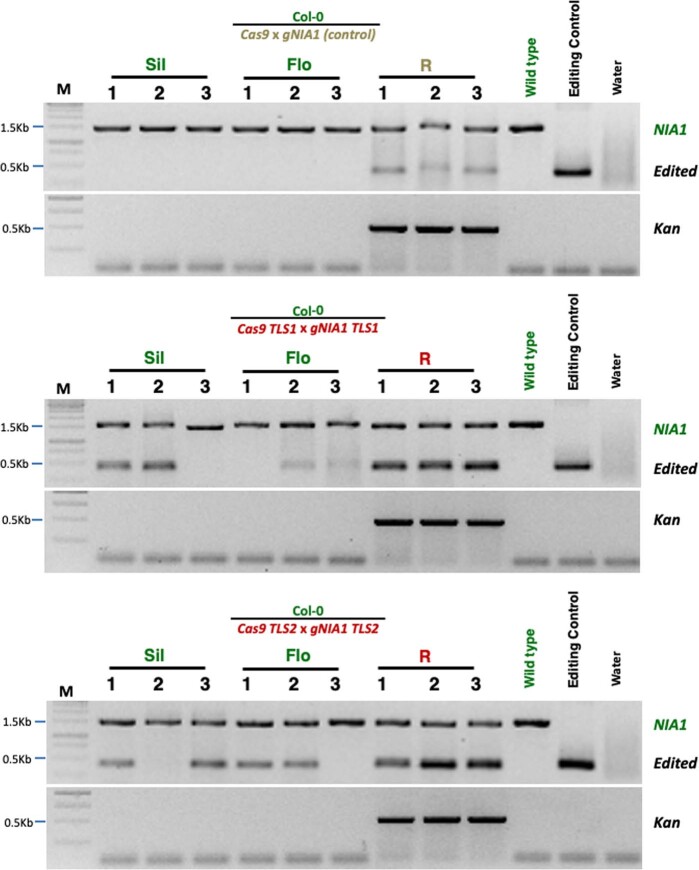
Root expressed mobile *Cas9* and g*NIA1* induce *NIA1* gene editing in the wild-type scion tissues of adult Arabidopsis. Genotyping PCR to detect *NIA1* edited fragments in siliques (Sil) and flowers (Flo). Genomic samples were from the same plant material as analyzed and shown in Fig. 3b. Expected wild-type *NIA1* fragment size is 1469 bp. Expected edited *NIA1* deletion fragment is approximately 430 bp. *Kanamycin* (*Kan*) gene-specific PCRs were used as a control confirming that the detected *NIA1* edits were not a result of contamination of wild-type tissues during harvesting. M: indicates molecular weight markers. Source data

Previous studies suggested that a dominant-negative acting *DISRUPTION OF MEIOTIC CONTROL 1* transcript interfering with meiosis fused to TLS motifs is graft transmissible from rootstocks to wild-type germline cells interfering with male sporogenesis in *Nicotiana* sp.^5^. Additionally, grafting experiments with mobile short interfering RNAs (siRNAs) showed that these are transported to meiotic precursor cells^14^. Therefore, we considered it to be likely that *Cas9*-TLS and *gNIA1*-TLS fusions can be delivered to wild-type flowers and cause heritable edits in the germline cells. Alternatively, these mobile fusions could be transported to meristems that would then give rise to a genome-edited lineage resulting in edited germline progenitor cells producing the next generation. To address this notion, we asked whether the *Cas9*-TLS and *gNIA1*-TLS fusion transcripts moved to leaves (cauline/rosette) and along the stem to reproductive tissues (siliques/flowers) in adult grafted plants. To detect the presence of the *Cas9* and gRNA transcripts we performed PCR with reverse transcription (RT–PCR) assays on RNA samples from adult grafted plants (Fig. 3a,b). These assays indicate that *Cas9* and *gNIA1* lacking TLS motifs were not moving to distant tissues (*n* = 4; independent replicates for each tissue). By contrast, both *Cas9* and *gNIA1* fused to either TLS1 or TLS2 were detected in three out of four samples of grafted plants in scion tissues, respectively (Fig. 3b and Extended Data Fig. 1). These findings were confirmed by quantitative PCR with reverse transcription (RT–qPCR) assays. Here, with wild type/*Cas9* × *gNIA1* grafts, we do not detect *Cas9* transcripts above technical background noise levels in wild-type siliques, flowers, stem, cauline, and rosette leaves (Fig. 3c). By contrast, *Cas9*-TLS1 × *gNIA1*-TLS1/wild type and *Cas9*-TLS2 × *gNIA1*-TLS2/wild type grafted plants, we detect *Cas9*-TLS transcripts in wild-type siliques, flowers, stem, cauline leaves, and rosette leaves. We estimated the transcript root-to-shoot delivery ratio using the RT–qPCR data, which indicates that approximately 1 out of 1,000 root-produced transcripts is delivered to non-transgenic shoot tissues (Table 1 and Fig. 3c). Here no significant difference was detected between *Cas9*-TLS1 and *Cas9*-TLS2 presence in the scion samples. We next analyzed whether the delivered fusion constructs are active by confirming the presence of genome edits in wild-type siliques and flowers by genomic PCR and Sanger sequencing of independent replicates (Fig. 3d,e and Extended Data Fig. 2). These assays revealed the presence of the expected *NIA1* genomic deletions in recipient wild-type tissues. This indicates that the delivered *Cas9*-TLS transcripts were translated into a functional protein in recipient tissues, and that both *Cas9*-TLS and *gNIA1*-TLS transcripts are delivered in sufficient amounts for effective genome editing of the *NIA1* gene in non-transgenic tissue of adult plants.

**Table 1:** *Cas9*-TLS transcript levels in transgenic roots versus grafted wild-type shoots as measured by RT–qPCR

Graft combination (wild-type scion/root)	Mean presence *Cas9*-TLS transcript in wild-type scions (s.d.^a^)	Mean presence *Cas9*-TLS transcript in transgenic roots (s.d.^a^)	Ratio (%) scion/root
*Arabidopsis*/*Arabidopsis*	110.86 (21.29)	122,017.63 (22,873.26)	0.091
*Brassica rapa*/*Arabidopsis*	560.36 (185.09)	119,753.66 (6,019.98)	0.468

^a^Note that no significant difference was found between TLS1 and TLS2 fusion constructs.

**Fig. 4:**
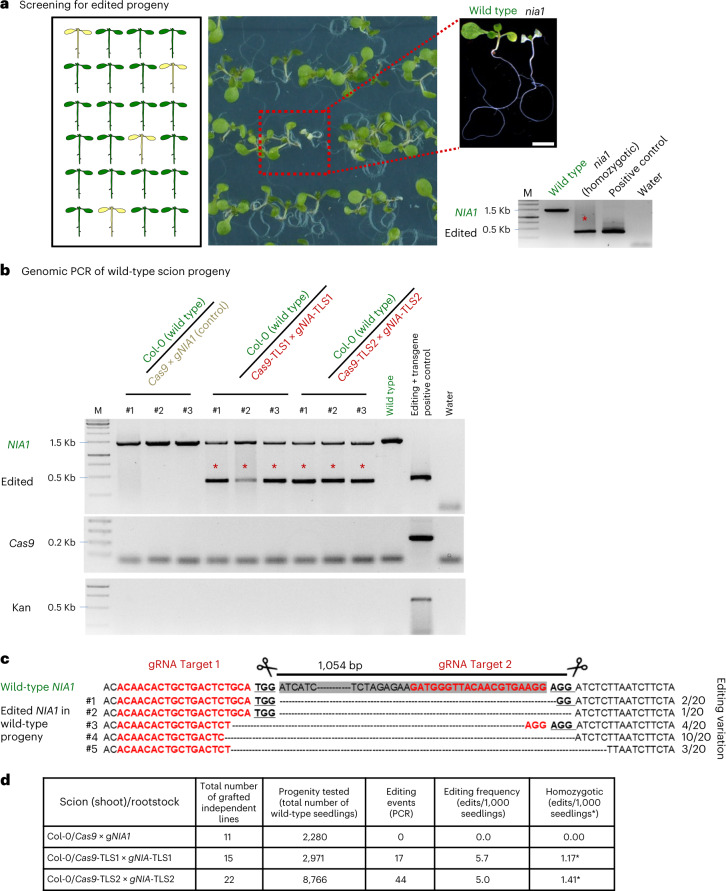
Gene edits are detected in the progeny of wild-type scions grafted on *Cas9* *×* *gNIA1* rootstocks. **a**, Screening for *NIA1* gene-edited grafted plants offspring and phenotyping for *nia1* homozygous mutants generated by mobile CRISPR–Cas9. Homozygotic *nia1* mutants were phenotypically screened from 14-day-old seedlings grown on NH_4_-limited plates and submitted to PCR assays. Scale bar, 5 mm. **b**, Genomic PCR to detect *NIA1* edits in the wild-type scion progeny. Edited *NIA1* PCR amplicons are marked by stars. All samples were harvested from 10-day-old seedlings grown on NH_4_-limited plates. Each replicate is a pool of ~70 to ~100 seedlings (Supplementary Table 1). *Cas9* and *Kan* transcript RT–qPCRs were used to confirm that the progeny was transgene-free. **c**, Transgene-free genome edits confirmed by Sanger sequencing. **d**, Editing efficiency analysis of *Cas9*-TLS*/*gRNA-TLS constructs in the progeny of grafted plants (Supplementary Table 1). Asterisk indicates the number of homozygotes identified in 1,000 seedlings. Source data

**Extended Data Fig. 3:**
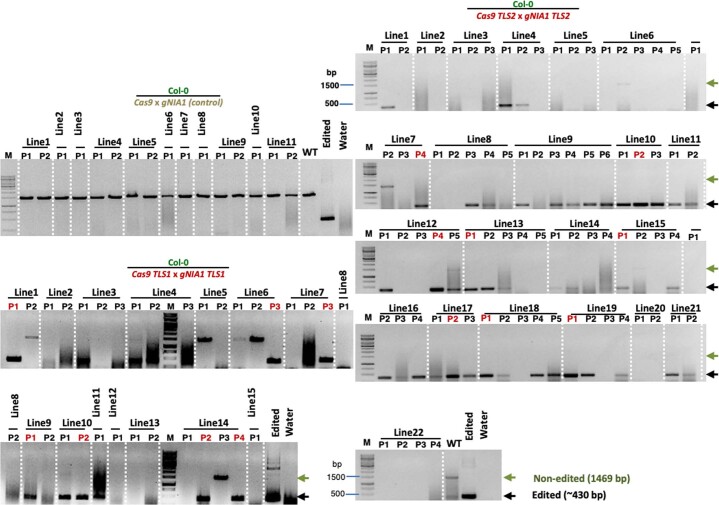
Identifying gene edits in the progeny of Col-0 / *Cas9* × *gNIA1* grafted plants. Genomic PCR to detect *NIA1* edited (deletion) fragments in the wild-type progeny of grafted plants. The smaller PCR amplicons are caused by mobile *Cas9* and *gRNA* - induced deletions in the *NIA1* gene. All samples were harvested from 10 days-old seedlings grown NH_4_ -depleted medium. Each pool contains genomic DNA of ~70 to ~100 seedlings (see Supplementary Table 1). To increase the detection sensitivity of the PCR all pooled samples - except Col-0 / *Cas9* x *gNIA1* control samples shown in the upper left - were digested by HindIII which targets the wild-type *NIA1* sequence but not the edited *NIA1* deletion sequence. Wild-type *NIA1* fragment size is 1469 bp and the edited deletion fragment is approximately 430 bp. Line # indicates individual grafted plants (lines) from which seeds were collected as pools (P) and analyzed as seedlings by genomic PCR. P # indicates pool of progeny seedlings deriving from a given grafted plant (see also Supplementary Table 1). Note that presence of edits (deletions) was confirmed by additional PCR assays and in part by Sanger sequencing are indicated in red (Fig. 4c). Source data

For the grafted plants, we next allowed the wild-type scions to set seeds, which were germinated on NH_4_-deficient medium to evaluate the appearance of *nia1* homozygous chlorotic leaf and small size phenotypes (Fig. 4a), and the presence of *NIA1* gene edits by genomic PCR and Sanger sequencing (Fig. 4b,c). As a *nia1* mutant phenotype is most noticeable when a plant is homozygous, we concluded that we were underestimating the frequency of genome editing inherited by the progeny. Therefore, we also examined the seedlings for the presence of the edited *NIA1* gene by genomic PCR to calculate the frequency of heritable genome edits created by graft-mobile *Cas9* and *gNIA1* (Fig. 4d, Extended Data Fig. 3 and Supplementary Table 1). In total 11 out of 15 grafts with Col-0 (wild-type) scions and *Cas9*-TLS1 *×* *gNIA1*-TLS1 rootstock and 17 out of 22 grafts with Col-0 scions and *Cas9*-TLS2 × *gNIA1*-TLS2 rootstock produced offspring with detectable genome edits. No *NIA1* edits were found in the progeny of 11 wild-type scions grafted on *Cas9* *×* gRNA lines lacking the TLS sequences. The calculated frequency of editing induced by mobile *Cas9*-TLS and *gNIA1*-TLS transcripts was between 5.7 (TLS1) and 5.0 (TLS2) per 1,000 seeds of which 1.17 (TLS1) and 1.41 (TLS2) per 1,000 seeds showed a clear *nia1* mutant phenotype indicating that these were homozygous (Fig. 4d). The TLS1 or TLS2 constructs caused no significant editing frequency difference according to Student’s *t*-test (two tails, *P* > 0.1) suggesting that both are similarly active in delivering editing factors to recipient tissues.

**Extended Data Fig. 4:**
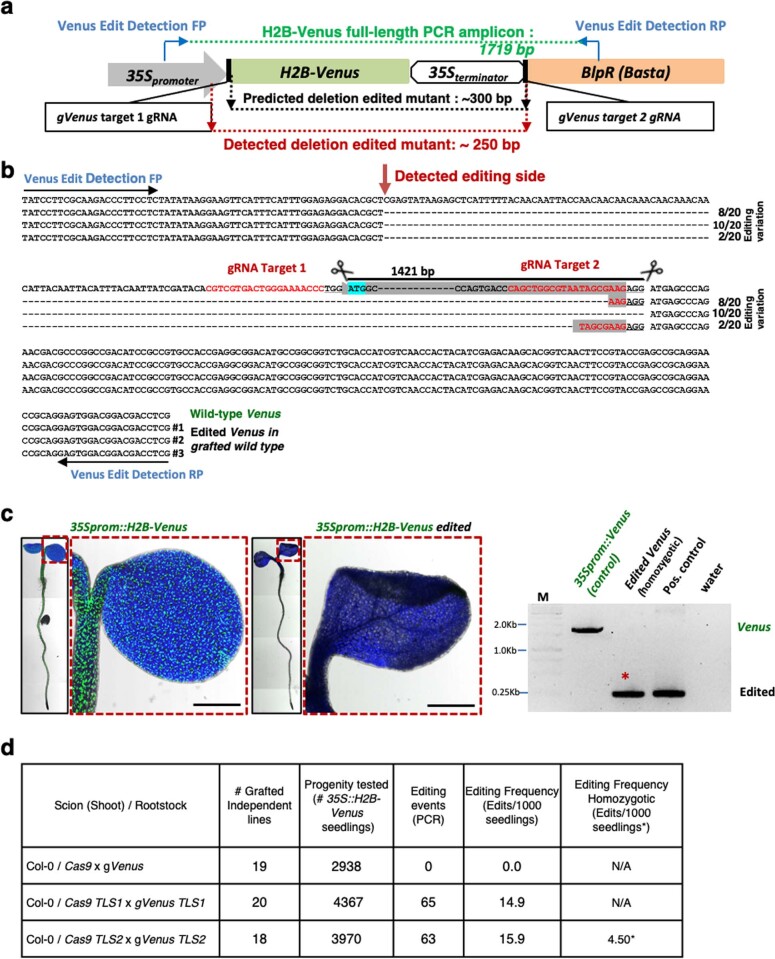
Gene edits detected in the progeny of wild-type scions grafted on *Cas9* x *gVenus* rootstocks. **a**, Schematic diagram showing the *35* *S*_*promoter*_*::H2B-Venus::35S*_*terminator*_*::Basta* cassette targeted by gVenus gRNAs (target1 and 2) and the predicted and PCR detected deletions. The black arrows indicate predicted and the red arrows indicate the detected PCR amplicons created by *gVenus target 1* and *gVenus target 2* gRNA constructs. Note that the predicted deletion edits were planned in such to remove the *H2B-Venus* sequence resulting in *Basta* resistance gene expression (see Supplementary Data 1d). However, the detected deletion edit eliminated part of the *35* *S* promoter sequence resulting in no expression of the resistance gene. **b**, Genome edits confirmed by Sanger sequencing. Red arrow indicates detected gRNA 1 mediated editing side which was not predicted, Light blue colored letters indicate the *Venus* start codon, Black arrows indicate used PCR primers to detected deletion edits. **c**, Confocal microscopy images of a 6-day-old F1 seedling harvested from a grafted *35S*_*promoter*_*::H2B-Venus::35S*_*terminator*_*::Basta* / Cas9-*TLS2 x gVenus-TLS2* parent showing no Venus fluorescence. This indicates homozygous elimination of the *H2B-Venus* sequence by deletion editing which was confirmed by genomic PCR assays (right panel). Scale Bar, 350 μm. M: indicates molecular weight markers. **d**, Editing efficiency analysis of Cas9-*TLS / gVenus-TLS* constructs in the progeny of grafted plants. # indicates the total number of grafted plants from which the progeny was tested (for details see Supplementary Table 2). * indicates the number of homozygotes identified by loss of Venus fluorescence per 1000 seedlings. Source data

To further confirm whether this graft-mobile gene editing system also works on other gene targets and thus in the context of other gRNA targeting sequences, we created two gRNAs, g*Venus1* and g*Venus2*, with and without fused TLS1 or TLS2 sequences. These gRNAs target an artificial *35S*_*promoter*_*::H2B-Venus::35S*_*terminator*_*::BastaR* construct (Extended Data Fig. 4a and Supplementary Data 1). After accurate double editing by both g*Venus1* and two gRNAs, this artificial construct should lose the *H2B-Venus::35S*_*terminator*_ sequences and consequently express the downstream *BastaR* resistance gene. Offspring of such gene-edited plants should gain dominant BASTA resistance and homozygous plants should lose Venus yellow fluorescence.

**Extended Data Fig. 5:**
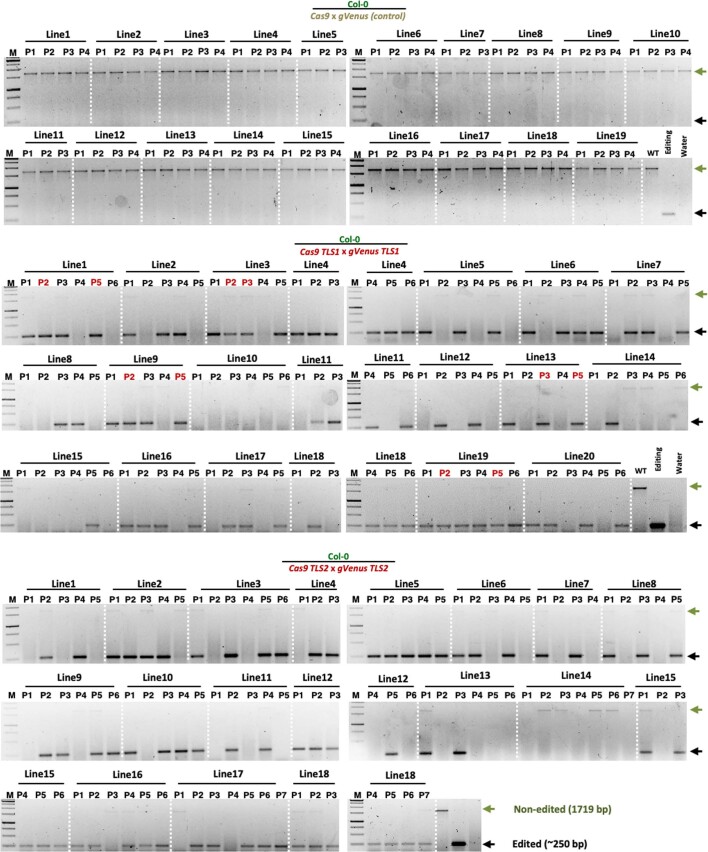
Identifying gene edits in the progeny of Col-0 / *Cas9* × *gVenus* grafted plants. Genomic PCR to detect *gVenus* edited (deletion) fragments in the wild-type progeny of grafted plants. The smaller PCR amplicons are caused by mobile *Cas9* and *gVenus* - induced deletions in the *Venus* gene. All samples were harvested from 10 days-old seedlings grown 0.5 MS medium. Each pool contains genomic DNA of about 40 seedlings (see Supplementary Table 2). To increase the detection sensitivity of the PCR all pooled samples - except Col-0 / *Cas9* x *gVenus* control samples were digested by *PstI* which targets the wild-type *Venus* sequence but not the edited *Venus* deletion sequence. Wild-type *Venus* fragment size is 1719 bp and the edited deletion fragment is approximately 250 bp. Line # indicates individual grafted plants (lines) from which seeds were collected as pools (P) and analyzed as seedlings by genomic PCR. P # indicates pool of progeny seedlings deriving from a given grafted plant (see also Supplementary Table 2). Note that presence of edits (deletions) confirmed by Sanger sequencing are indicated in red letters. Black arrows indicate detected size of *Venus* edited bands of approx. 250 bp. Green arrows indicate *Venus* wild-type expected bands of 1719 bp. M: indicates molecular weight markers. Source data

We grafted rootstocks expressing Cas9 and g*Venus* with and without TLS fusions with *35S*_*promoter*_*::H2B-Venus::35S*_*terminator*_*::BastaR* transgenic scions and examined the offspring (seedlings) for the presence of the edited *Venus* gene by genomic PCR and in part by Sanger sequencing to calculate the frequency of heritable genome edits created by graft-mobile *Cas9* and g*Venus* (Extended Data Figs 4b and 5, and Supplementary Table 2). In total 19 out of 20 grafts with *35S*_*promoter*_*::H2B-Venus::35S*_*terminator*_*::BastaR* transgenic scions and *Cas9*-TLS1 *×* *gVenus*-TLS1 rootstock and 17 out of 18 grafts with *35S*_*promoter*_*::H2B-Venus::35S*_*terminator*_*::BastaR* transgenic scions and *Cas9*-TLS2 *×* *gVenus*-TLS2 rootstock produced offspring with detectable genome edits. Again, no edits were found in the progeny of 19 wild-type scions grafted on *Cas9* *×* *gVenus* lines lacking the TLS sequences. The calculated frequency of editing induced by mobile *Cas9*-TLS2 and *gVenus*-TLS2 transcripts was above 15.9 per 1,000 seeds (Extended Data Fig. 4d and Supplementary Table 2). However, none of the edited offspring showed the anticipated BASTA resistance. This was because the *gVenus*1 gRNA cut the *35S*_*promoter*_ target region further upstream as predicted which resulted in a truncated *35S* promoter (Extended Data Fig. 4a,b). Thus, the deletion edit did not activate expression of the downstream *BastaR* gene providing resistance. We next analyzed the presence of Venus fluorescence in the offspring of three homozygotic *35S*_*promoter*_*::H2B-Venus::35S*_*terminator*_*::BastaR* scions grafted on *Cas9*-TLS2 × *gVenus*-TLS2 rootstocks. Here we detected loss of Venus fluorescence in 7 of 1,557 offspring seedlings indicating presence of homozygous gene edits in approximately 0.45% of plants (Extended Data Fig. 4c,d). In summary, these results indicate that the mobile CRISPR–Cas9 transgene-free genome editing is also functional with alternative gRNA sequences and that without the addition of TLS motifs it is not introducing heritable gene edits in grafted wild-type scions.

## *Cas9/gRNA-TLS* moves from Arabidopsis roots to *Brassica rapa* shoots

**Fig. 5:**
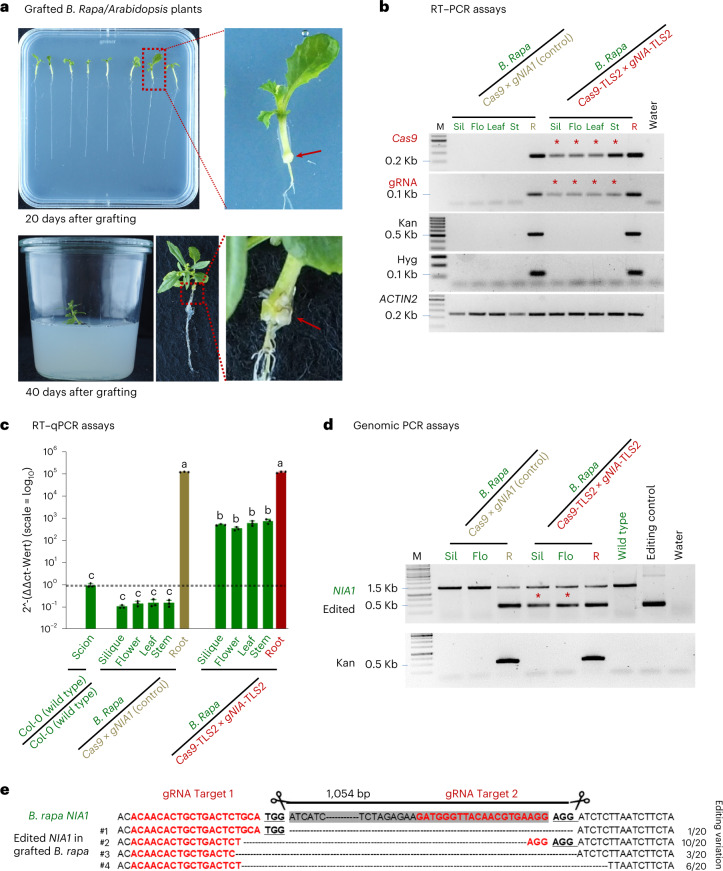
*Cas9*- and g*NIA1*-TLS2 fusion constructs are mobile and functional in *B. rapa*/*Arabidopsis* grafted plants. **a**, Appearance of grafted plants (20 and 40 days after grafting) grown on MS medium and treated with 5 μM estradiol. Red arrows indicate the graft junction. **b**, RT–PCR detection (45 PCR cycles) of *Cas9* and *gNIA1* transcripts in rootstock, and grafted wild-type tissues samples from silique, flower, leaf, and stem from one independent replicate (the other three replicates are presented in Extended Data Fig. 3) was analyzed. Stars, RT–PCR *Cas9*-TLS and *gNIA1*-TLS amplicons detected in *B. rapa* wild-type scion samples. Kan and Hyg transcripts serve as contamination controls. Note that transcript absence was confirmed by 50 PCR cycles. **c**, RT–qPCR detection of mobile *Cas9*-TLS transcripts in scion tissues and in grafted rootstock (Root). *y*-axis, mean $2^{-\Delta\Delta c_t}$ values; log_10_ scale. Each value represents the mean of three independent biological replicates presented as black dots on the bar. Note that every $2^{-\Delta\Delta c_t}$ value below the dashed line represents technical background as measured with wild-type scion samples grafted onto wild-type rootstocks with no *Cas9* transcript present. Significance was calculated using Student’s *t*-test (two tails); *P* values indicated by a, b and c: a,b = 3.48073 × 10^−8^; b,c = 2.29801 × 10^−7^; a,c = 3.43932 × 10^−8^. **d**, Genomic PCR to detect *NIA1* edited fragments in siliques and flowers. Samples were from the same plant material as analyzed in **b**,**c**. **e**, Mobile CRISPR–Cas9*-*induced genome edits confirmed by Sanger sequencing. Source data

We next asked whether the mobile *Cas9*-TLS and gRNA-TLS constructs are also transported to a distantly related crop species grafted onto transgenic Arabidopsis roots. To address this, we grafted *Brassica rapa*, a worldwide cultivated vegetable and oilseed crop, with the Arabidopsis lines expressing the mobile and non-mobile *NIA1*-targeting gRNAs. As the *NIA1* gene sequence is conserved between *Arabidopsis* and *Brassica rapa* we would expect to detect *Cas9*-TLS and gRNA-TLS transcripts and *NIA1* edits in shoots of grafted *B. rapa*/*Cas9*-TLS *×* *gNIA*-TLS plants. To test this, we hypocotyl-grafted *Arabidopsis* *Cas9* *×* *gNIA1* or *Cas9*-TLS2 × *gNIA1*-TLS2 rootstocks with *B. rapa* scions (Fig. 5a) and confirmed successful healing of the graft junction 10, 20, and 40 days after grafting by slightly pulling the scion and rootstock. This cross-species hypocotyl grafting protocol was very successful with 13 out of 15 *B. rapa*/*Cas9* × *gNIA1* and 15 out of 16 *B. rapa*/*Cas9*-TLS2 × *gNIA1*-TLS2 grafted plants passing the graft take test.

**Extended Data Fig. 6:**
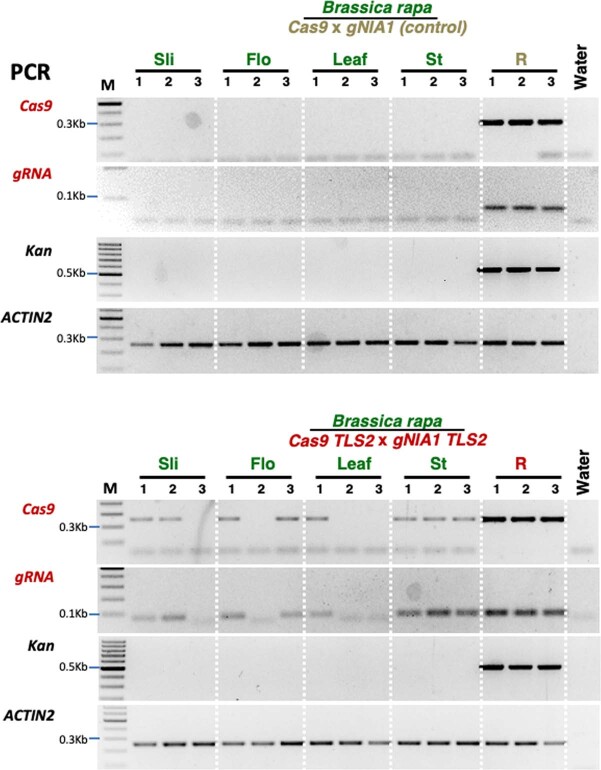
*Cas9* and g*NIA1* transcript presence in grafted *Brassica rapa* / Arabidopsis wild-type scions. Tissue samples from Arabidopsis transgenic roots (R) and *Brassica rapa* wild-type silique (Sil), flower (Flo), leaf, stem (St) of grafted plants were used for RT-PCR detection of *Cas9-TLS2* and *gNIA1-TLS2* fusion transcripts. For each graft combination three independent replicates (each sample was a pool of two grafted plants) are presented which were tested by RT-PCR (45 PCR cycles) for the presence of fusion transcripts. *Kanamycin* (*Kan*) transcript expressed only in grafted transgenic rootstocks was used as contamination control in RT-PCR assays. Note that *Kan* transcript absence was additionally confirmed by 50 PCR cycles. M: indicates molecular weight markers. Source data

**Extended Data Fig. 7:**
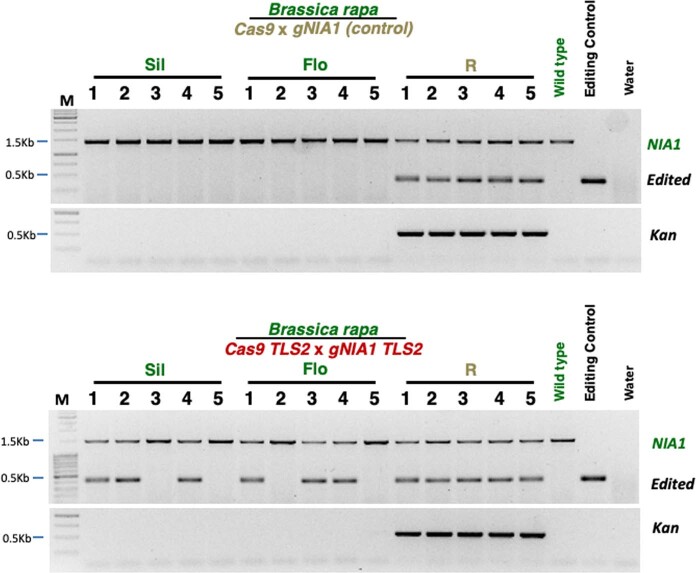
Root expressed mobile *Cas9* and g*NIA1* induce *NIA1* gene editing in the wild-type scion tissues of adult *Brassica rapa*. Genotyping PCR to detect *NIA1* edited fragments in siliques (Sil) and flowers (Flo). Genomic samples were from the same plant material as analyzed and shown in Fig. 5b, c, and Extended Data Fig. 6. The expected wild-type *NIA1* fragment size is 1469 bp. The expected edited *NIA1* deletion fragment is approximately 430 bp. *Kanamycin* (*Kan*) gene-specific PCRs were used as controls confirming that the detected *NIA1* edits were not a result of contamination of wild-type tissues during harvesting. M: indicates molecular weight markers. Source data

We next asked whether *Cas9* and/or *gNIA1* transcripts, with or without a TLS transport motif are also root-to-shoot mobile in these heterografted plants. We sampled RNA and gDNA from siliques, flowers, stem, leaves, and roots (*n* = 4, two grafted plants pooled for each sample) 40 days after grafting and submitted the samples to RT–PCR and genomic PCR assays (Fig. 5a). In *B. rapa* scion samples from plants grafted on *Arabidopsis* *Cas9* *×* *gNIA1* transgenic roots we did not detect *Cas9* or gRNA transcripts (*n* = 4, each sample pool of two grafted plants), although both were present in the rootstock samples (Fig. 5b,c and Extended Data Fig. 6). By contrast, in the *B. rapa* siliques, flowers, stem, and leaf samples harvested from plants grafted onto *Cas9*-TLS2 × *gNIA1*-TLS2 rootstocks, we detected both *Cas9*-TLS2 and *gNIA1*-TLS2 (*n* = 4, each sample pool of two grafted plants) (Fig. 5b,c and Extended Data Fig. 6). We next measured the *Cas9* transcript levels in *B. rapa* siliques, flowers, stem, leaf, and *Arabidopsis* root tissue samples by RT–qPCR. In *B. rapa* samples harvested from plants supported by *Cas9* *×* *gNIA1* rootstocks no *Cas9-*specific transcript could be detected. In *B. rapa* samples from plants supported by *Cas9*-TLS2 × *gNIA1*-TLS2 rootstocks we found relatively high levels of *Cas9*-TLS2 transcripts (Fig. 5c) as indicated by the higher shoot/root ratio of ~0.4 (Table 1) compared to homografted *Arabidopsis* plants showing a ratio of ~0.09 (Table 1). Thus, the TLS motif was efficiently promoting long-distance mobility of the *Cas9* and gRNA transcripts from *Arabidopsis* rootstocks to *B. rapa* scions. We also tested whether mobile *Cas9-TLS* and gRNA-TLS fusions are functional in introducing deletion edits into the conserved *B. rapa NIA1* gene. Consistent with the findings with heterografted *Arabidopsis*, we detected genome edits in four out of six siliques and in four out of six flowers formed on grafted *B. rapa* plants by genomic PCR and Sanger sequencing (Fig. 5d,e and Extended Data Fig. 7). These findings suggest that both *Cas9*-TLS and *gNIA1*-TLS constructs are transported in sufficient amounts and functional in editing the genome of a heterografted crop plant.

## Discussion

We have demonstrated a simple, effective method for creating heritable transgene-free genome edits using grafted plants. TLS motifs fused to *Cas9* and gRNA mediate their transport from transgenic roots to grafted wild-type shoots. The grafted recipient wild-type tissues show edited genomes and their flowers produce seeds with inherited edited genomes. The detected efficiency of inherited deletion edits in the range of ~0.1% for homozygotic and of ~1.6% for heterozygotic edits was reasonably high, although these numbers are most likely an underestimation as pools of approximately 100 or 40 seedlings for *NIA1* or *Venus* edits were analyzed, respectively (Fig. 4d and Extended Data Fig. 5). Indeed, as a deletion depends on two gRNA edit sites, the actual editing efficiency, including single position edits, induced by mobile *Cas9* and gRNAs in wild-type scions, is most likely underestimated. Given that multiplex PCRs allow for fast screening of edits in a large number of seedlings deriving from grafted plants, homozygous mutants can be identified in a relatively short time. Notably, the presented approach is not only useful for plant researchers to generate stable transgene-free lines harboring, for example, multiplex genome edits^15^, but also for more complex applications. For example, as not all somatic cells will be edited in grafted wild-type scions, this also constitutes a chimeric somatic mutant allowing for the analysis of otherwise lethal mutations.

The z*Cas9* construct used in this study, with approximately 4,200 bases in length, is currently the longest mRNA that has been made mobile by the addition of an RNA mobility motif, and *Cas9*-TLS is one of the largest mobile mRNAs reported for *Arabidopsis*^16^. Further, our data show that mobile *Cas9*-TLS and gRNA-TLS transcripts are delivered to heterologous plants and that root-produced *Cas9*-TLS transcripts are indeed translated into a functional editing enzyme in recipient scion cells. We also noticed that approximately 1 of 250 *Arabidopsis*-root-produced *Cas9*-TLS transcripts is detectable in *B. rapa* scion tissues as estimated from RT–qPCR data (Table 1 and Extended Data Fig. 5c), implying that a reasonably high number of *Cas9*-TLS transcripts are actually delivered over graft junctions. While the exact mechanisms of TLS-induced mRNA mobility from roots to shoots remain to be elucidated, we have demonstrated that mobile TLS fusion constructs move to all major shoot tissues such as rosette and cauline leaves, stem, flowers, and seed-bearing siliques (Fig. 3b) where they can induce genome editing. We also noticed that, although siliques seem to receive lower amounts of mobile *Cas9*-TLS transcript, they appear to show more genomic deletions than flowers or rosette leaves (Extended Data Fig. 2). We hypothesize that the appearance of more gene edits in the seed producing siliques is either due to their clonal origin from flower meristems or the capacity of meiocytes to receive graft-mobile transcripts, as seen with gene-silencing-inducing siRNAs^14^. In line, it is known that addition of a TLS motif can promote long-distance mRNA mobility in *Arabidopsis* and *Nicotiana tabacum*. In both species, a dominant-negative acting *DISRUPTION OF MEIOTIC CONTROL 1* (_*DN*_*DMC1*) variant, which lacks the N-terminal 92 amino acid residues and therefore interferes with meiotic progression, fused to TLS is transported from grafted rootstocks to wild-type flowers and interferes effectively with meiosis in recipient meiocytic cells^5^. Other mobile RNA motifs, derived from *FLOWERING LOCUS T* (*FT*) transcripts, when fused to gRNAs seem to license transport of viral expressed gRNAs in *A. thaliana*, *Nicotiana sp*., and wheat^12,17^. Therefore, RNA mobility mediated by RNA transport motifs seems to be a conserved mechanism which can be used across a wide range of plant families and crop species. Although it seems unlikely and has to be experimentally shown, one could speculate that *Cas9* mobility might be increased by adding multiple TLS motifs or alternative relatively short RNA mobility motifs identified in other mobile mRNAs, such as *TRANSLATIONALLY CONTROLLED TUMOR PROTEIN 1* (*TCTP1*), that mediate transport of heterologous transcripts^18^.

The major task in gene editing of plants is to generate transgene-free edited offspring to ensure genomic stability and their use in food production. For this purpose, one must eliminate all editing components from the edited crop plant before their commercial release. Thus, elimination of exogenous DNA components has become one of the major objectives in gene editing research. This can be achieved by local expression of Cas9/gRNA-related gene components using Agrobacterium-mediated transformation^19,20^ or plant-virus-mediated gene editing^21,22^, or by directly delivering active Cas9 protein–gRNA complexes into plant cells^18,23–26^. The disadvantages of these systems are that many crop plants and fruit trees are inaccessible or that they are difficult to be transiently infected or transformed. For example, in the previous methods the edited plant material still contains the *Cas9* gene and/or remains contaminated by transgenic bacteria or viruses. This is undesirable for commercial crop production owing to expensive containment and time-consuming elimination protocols that have to be implemented and the risks involved by using genetically modified viruses in production pipelines.

Here we present an alternative, rapid method for producing heritable genome edits induced by graft-mobile TLS fusion transcripts. The presented grafting system does not need elimination of gRNA or *Cas9* transgenes and regeneration of plants from tissues or protoplasts and, thus, the desired mutation can be produced and used after the first generation. Alternative approaches resulting in transgene-free edited plant material rely either on delivery of active Cas9–gRNA protein–RNA complexes into callus cells, protoplasts, or immature embryos followed by elaborative and time-consuming regeneration and selection of edited plant material for further use. Again, this is difficult or not possible for most crop species. In the presented grafting system, *Cas9*-TLS mRNA and gRNA*-*TLS fusions are delivered directly to germline progenitor cells where they are actively editing the genome. This allows for producing edited seeds with no need for transgene or virus elimination.

The gene editing method presented here, despite the required production of a transgenic rootstock line, is not more time consuming than alternative transgene-free genome editing methods. Gene editing by grafting confers an advantage as there is no requirement for multiple additional generations to remove the transgene or regeneration of plants from transfected protoplasts. For example, by our estimates, it takes approximately 3 months to generate a T1 transgenic *Arabidopsis* rootstock line that can be used as a gene editing donor, another 3 to 4 months to generate seeds and to select by genomic multiplex PCR individual edited lines. In comparison to an alternate transgene-free editing method using protoplast transfection with pre-assembled ribonucleoprotein complexes^26^, we find that the time required is similar—approximately 3 months to generate edited plantlets, then an additional 3 to 4 months to produce and select the next generation of seeds. While that method is efficient, one should also consider that protoplast regeneration protocols do not exist for a wide variety of plant species and that protoplast regeneration has been observed to induce genome instability^27^, which has to be assessed in the following generations. Though both the grafting method and the protoplast method each have advantages and disadvantages, the total time required for both methods is similar. The grafting system also permits for the introduction of edits in any graft-compatible cultivar that can be combined with a given *Cas9*-TLS/gRNA-TLS expressing rootstock. While the production of a transgenic rootstock is time consuming, any rootstock species that is graft-compatible and easily transformable can be used as a donor for the graft-mobile gene editing system. Also, once established, a rootstock line can be easily maintained and distributed and used as a donor plant line for any variety or graft-compatible crop species of particular interest for breeders. That this is feasible we have shown by using transgenic *Arabidopsis* rootstocks as donors to introduce gene edits to grafted *B. rapa* scions that show different growth and flowering behavior.

As mentioned above, the introduction of gene edits by grafting would greatly simplify the production of mutant plants where transformation is difficult, impossible or undesirable. The wild-type scion cultivar grafted onto a given transgenic rootstock donor can belong to a very distant plant family. For example, *A. thaliana* and *Nicotiana sp*. can easily be used as rootstocks for a very wide range of quite distantly related species including tomato, carrots, soybean, and onions^28^. Although both species are good donor plants that can be grafted with many crop species, there is still a limitation for their use with distantly related species. One has to consider that the donor (stock) produced gRNA sequences must be optimized to match the gene sequences of more distantly related species to warrant sufficient sequence similarity for successful editing. Thus, it might be required to produce additional transgenic donor lines expressing optimized gRNAs sequences specifically matching the target gene(s) of heterologous scions.

In light of the recent study that demonstrates grafting in monocotyledonous species^29^, we can further speculate that this technique based on mobile *Cas9*-TLS and gRNA-TLS fusions will find use in main crops such as maize, wheat, and rice. In addition, a graft-transmissible editing system could be used in many agricultural settings as grafting is a major technological platform in commercial agricultural productions^30^.

## Methods

### Plasmid construction

The *rbcs-e9t* terminator sequence was PCR amplified from pJF3101 (a modified version of pHEE2E-TRI)^10^, using primers *rbcsT* FP and *rbcsT* RP (Supplementary Table 3). The PCR product was digested with BsaI to produce two XhoI-compatible ends, such that the 3′ end creates a non-functional scar site in place of the XhoI site, leaving the final vector with one remaining XhoI site at the 5′ end of the *rbcs-e9t* sequence. This was cloned into the estradiol-inducible binary vector pMDC7^31^ at the XhoI site, and the orientation of insert was confirmed by digestion with XhoI and AscI. This intermediate vector was termed pMDC7_rbcs-e9t.

**Extended Data Fig. 8:**
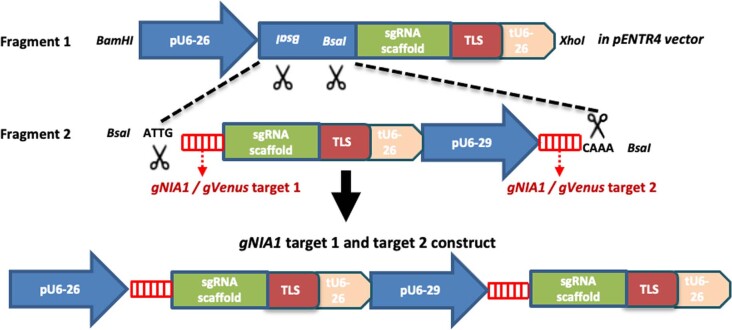
Schematic diagram showing the construction of *gNIA1* expression cassette. Schematic diagram showing the construction of gNIA1 expression cassette.

We used the *Cas9* (*zCas9*) sequence from pJF1031^10^. This *Cas9* sequence features an optimized codon usage for plants, a nuclear localization signal, and a FLAG-tag at the 3′ end of the *Cas9* coding sequence. The *Cas9* and *Cas9*-TLS fusion sequence fragments were made by PCR amplification using *Cas9* FP and *Cas9* RP, *Cas9-ΔDT* RP or *Cas9-tMet* RP primers (Supplementary Table 3) containing BsaI sites with compatible overhangs (Extended Data Fig. 8). Amplification was done using Phusion DNA polymerase (Thermo Fisher) with the addition of 3% dimethylsulfoxide to prevent folding of the TLS primers. Amplified regions were cloned into CloneJet Blunt end ligation vector (Thermo Fisher), confirmed by sequencing, and cloned into the XhoI site in pMDC7_*rbcs-e9t* binary gateway vector using the compatible BsaI sites at the 5′ and 3′ end. The pMDC7 *Cas9* clones were transformed into DB3.1 *E. coli* strain owing to the toxicity of the *ccdb* gene present in pMDC7^30,31^.

The gRNA backbone sequences Fragment 1 and Fragment 2 were created by gene synthesis (Eurofins) (Extended Data Fig. 8). Fragment 1 (with or without TLS motifs) was cloned into pENTR4 using BamHI and XhoI. Fragment 2 sequences were used as PCR templates for amplification with *NIA1* and *Venus* target sequence primers with BsaI cut sites (*NIA1* Fragment 2 FP/RP and *Venus* Fragment 2 FP/RP; Supplementary Table 3). Fragment 2 (with *NIA1* and Venus target sequences added) were cloned into Fragment 1 using BsaI restriction sites with compatible overhangs. Completed gRNA (*gNIA1* and *Venus* target 1 and 2*)* expressing sequences in pENTR4 were then transferred into pMDC100^31^ by Gateway cloning (LR reaction). The *35S*_*promoter*_*::H2B-Venus:35S*_*terminator*_*:Basta*:*Nos*_*terminator*_ binary plasmid was constructed by gene synthesis and entry vector gateway cloning into the pRI 909 destination vector. All final constructs in the binary vectors were confirmed by Sanger sequencing and are listed in Supplementary Table 4.

### Generation of transgenic arabidopsis lines

Binary vector constructs were transformed to *Agrobacterium tumefaciens* strain AGL1 subsequently used to transform *Arabidopsis* Col-0 by the floral dip method^32^. T1 seedlings were selected on 0.5 MS, 0.5% sucrose plates solidified with 0.68% micro agar (Duchefa Biochemie). *gNIA1*, *gNIA1*-TLS1*, gNIA1*-TLS2*,* g*Venus*-TLS1, or g*Venus*-TLS2 expressing transgenic lines were crossed with transgenic lines harboring estradiol-inducible *Cas9*, *Cas9*-TLS1, or *Cas9*-TLS2 sequences, respectively. The antibiotics used for each construct can be found in Supplementary Table 2. Lines were screened for 3:1 segregation on antibiotic selection medium and the absence of visible growth phenotypes.

### Hypocotyl grafting of arabidopsis with arabidopsis

*Arabidopsis thaliana* Col-0 wild-type and transgenic seeds were placed on plates containing 0.5 MS salts, 1% sucrose, and 1% micro agar (Duchefa Biochemie) and vertically grown in short-day conditions (8 h light/16 h dark; day 22 °C/night 19 °C; light intensity: 170 μE m^−2^ s^−1^). Seedlings (6–7 days after germination, DAG) with ~4 cm long roots were cut in the upper half of the hypocotyl using a sterile razor blade. Silicon micro-tubes (0.3 mm internal diameter) were used to stabilize the graft junction. Grafted plants were transferred onto new plates (0.5 MS salts, 0% sucrose, 1% micro agar (Duchefa Biochemie), and 5 µM estradiol) and vertically grown in short-day conditions. Beginning at 6–7 days after grafting, adventitious roots appearing on the upper (scion) hypocotyl junction were removed every day. Twenty-one days after grafting (young) plants were harvested from plates for RNA and DNA isolation, or 14 days after grafting plants were transferred to soil with 5 µM estradiol (16 h light/8 h dark), 43 days after grafting (adult flowering) plants were harvested for RNA and DNA isolation, or kept to produced seeds.

### Hypocotyl grafting of *B. rapa* with *Arabidopsis*

Sterilized *Arabidopsis thaliana* Col-0 transgenic seeds were placed on plates containing 0.5 MS salts, 1% sucrose, and 1% micro agar (Duchefa Biochemie) and vertically grown in short-day conditions (8 h light / 16 h dark; day 22 °C / night 19 °C; light intensity: 170 μE·m − 2·s^−1^). 7 days after preparing Arabidopsis seeds, sterilized *B. rapa* (RCBr) wild-type seeds (provided by the John Innes Centre, UK) were placed on plates containing 0.5 MS salts, 1% sucrose, and 1% micro agar (Duchefa Biochemie) and vertically grown in long-day conditions (16 h light/8 h dark; day 22 °C/night 19 °C; light intensity: 170 μE m^−2^ s^−1^). Two-week-old *Arabidopsis* seedlings and one-week-old *B. rapa* seedlings were used for grafting. *Arabidopsis* plants were cut in the lower half of the hypocotyl for use as a rootstock. To prepare *B. rapa* scions for grafting the cotyledons were removed and then the hypocotyl was cut 2 cm below the shoot apical region. The *Arabidopsis* rootstock and *B. rapa* scion were aligned together on a new plate containing 0.5 MS salts, 1% sucrose, and 1% micro agar (Duchefa Biochemie). Grafted plants were grown under long-day conditions with plates in a vertical orientation (5–10°). The graft success was evaluated 10 and 14 days after grafting by gently pulling the scion and rootstock apart. Fourteen days after grafting plants were transferred to new 0.5 MS plates (1% sucrose, and 1% micro agar) supplemented with 5 µM estradiol to induce gene expression in the root and grown under long-day conditions (16 h light/8 h dark; day 22 °C/night 19 °C; light intensity: 170 μE m^−2^ s^−1^). Twenty-five days after grafting plants were transferred to 0.5 MS agar medium (1% sucrose, supplemented with 5 µM estradiol) in glass jars and grown till flowering (approximately 40 days after grafting) and harvested for RNA and genomic DNA isolation (Fig. 5).

### Genomic DNA extraction and genomic edits detection by PCR

Plant tissue was harvested, frozen in liquid nitrogen, and stored at −80 °C in Eppendorf tubes until extraction. Frozen tissue was incubated in DNA extraction buffer, manually broken, and centrifuged to remove debris. The supernatant was transferred to a new tube and extracted with an equal volume of phenol:chloroform:isoamyl alcohol (25:24:1, v/v), homogenized and centrifuged for 10 min (13,000*g*). The upper phase was transferred to a new tube, mixed with four volumes of cold isopropanol, and DNA was precipitated at −20 °C overnight and collected (15 min, 13,000*g*). The DNA pellet was washed twice with ice-cold 70% ethanol and resuspended in 25 µl distilled water.

Specific PCR primers for *NIA1* deletion edits (*NIA1* Edit Detection FP/RP*)* and *VENUS* deletion edits *(VENUS* Edit Detection FP/RP; Supplementary Table 1) were used to amplify the region surrounding the predicted targeted edit site, producing a 1,469 bp (*NIA1*) and 1,719 bp (*Venus*) band in the wild-type genome with approximately 430 bp and 250 bp in the edited (deletion mutant) genome, respectively. Fifty nanograms of gDNA was used for a 20 μl PCR reaction with Phusion DNA Polymerase (NEB #M0530). PCR annealing temperature of 55 °C and extension time of 5 s was used to promote amplification of the smaller gene-edited fragment. Forty-five PCR cycles were used unless otherwise stated. To confirm the identity of bands, PCR fragments were isolated from the gels and ligated into CloneJet blunt-end ligation vector (Thermo Fisher) before being transformed to *E. coli*. Twenty clones were selected for each graft and were submitted to Sanger sequencing (LGC Genomics).

### RNA isolation and RT–PCR

Plant tissues, either from juvenile grafted plants or from adult flowering grafted plants were independently collected in 2-ml Eppendorf tubes containing metal beads. After freezing in liquid nitrogen, 750 µl TRIzol Reagent (Invitrogen) was added before samples were homogenized and incubated at room temperature for 5 min. Chloroform was then added (0.2 ml per 0.75 ml TRIzol) and homogenized by vortexing. The upper (aqueous) phase was transferred to 1.5-ml tubes and 1 volume of isopropanol and 0.1 volumes of 3 M NaOAc (pH 5.2) were added to precipitate total RNA. RNA pellets were then washed twice with 80% ethanol and then once with 99% ethanol. The RNA pellets were resuspended in 25 µl DEPC-treated water.

Reverse transcription was done using AMV Reverse Transcriptase (Promega). Approximately 1.5 µg isolated total RNA was used per reaction. Oligo(dT) primer was used for cDNA production of mRNAs including *Cas9*, kanamycin, hygromycin, and *ACTIN2*, a gRNA-specific primer (gRNA RT-RP; Supplementary Table 2) was also included for reverse transcription of *gNIA1* gRNAs to facilitate detection by PCR. The total RNA was denatured at 70 °C for 10 min followed by annealing incubation for 5 min at 37 °C. The RT reaction was done at 42 °C for 90 min followed by inactivation at 72 °C for 10 min.

RT–PCR was done using Phusion polymerase according to the standard protocols. *ACTIN2* primers (Supplementary Table 2) and *ACTIN2* amplification was done with 30 PCR cycles and to confirm cDNA quality. PCRs with primers for detecting *Cas9* and gRNA mobility were done using 45 PCR cycles for increased sensitivity. To confirm signal (amplicon) absence 50 PCR cycles were used unless specified otherwise in the text. As a control for tissue contamination and specificity of transport, we used the hygromycin and kanamycin resistance genes as targets for RT–PCR as these transcripts were shown to be not root-to-shoot graft transmissible in this study.

### RT–qPCR

To measure *Cas9* transcript levels by RT–qPCR we used an Applied Biosystems 7900HT fast Real-time PCR machine with SYBR Green as readout following the supplier’s manual. For all assays three technical replicates were performed. The program of thermal cycling was as follows. Step 1: 1 cycle, 2 min at 50 °C. Step 2: 1 cycle, 10 min at 95 °C. Step 3: 40 cycles, 15 s at 95 °C, 1 min at 60 °C. Dissociation step: 15 s at 95 °C, 15 s at 60 °C, 15 s at 95 °C. *UBQ10* gene was used as the reference. *Cas9* transcript expression levels presented by $2^{-\Delta\Delta c_t}$ (ΔΔ*c*_t_ = ∆*c*_*t*_ (*Cas9*) − ∆*c*_*t*_ (control average)); ∆*c*_*t*_ (*Cas9*) = *c*_*t*_ (*Cas9*) − *c*_*t*_ (*UBQ10*); ∆*c*_*t*_ (control) = ∆*c*_*t*_ (∆*c*_*t*_ average of wild-type grafted scions) following established protocols^33^. The bar plots in Fig. 3c were created using the GraphPad Prism9 software. *Cas9* and *UBQ10* qPCR primer sequences used see Supplementary Table 3.

### *nia1* Phenotype screening

Progeny seeds from grafted plants were surface sterilized by washing in 70% ethanol followed by washing in 100% ethanol and allowed to dry. Seeds were placed on NH_4_-free medium (see Supplementary Table 4), stratified at 4 °C for 2 days, and transferred to a growth chamber for germination (16 h light/8 h dark; day 22 °C/night 22 °C). Seedlings were grown for 14 days, and candidate seedlings showing a *nia1* phenotype were transferred to new plates containing 0.5 MS, 1% sucrose, and 1% micro agar (Duchefa Biochemie) (Supplementary Table 5) for 2 weeks before being transferred to soil. Leaf pieces were then harvested for genomic DNA extraction and genomic DNA was used for PCR assays to confirm the presence of gene edits.

### Measurement of genome edit efficiency

F1 seeds were sterilized as described above and sown on 0.5 MS medium containing 1% sucrose and solidified with 0.68% micro agar. Plates were stratified at 4 °C for 2 days before transfer to a growth chamber (16 h light/8 h dark; day 22 °C/night 22 °C). Ten days after germination, seedlings were harvested for genomic DNA isolation. Approximately 70 to 100 (for *NIA1* edits) or approximately 40 (for *Venus* edits) seedlings were pooled together (Supplementary Table 1) and genomic DNA extracted as described above, except the DNA pellets were resuspended in 100 µl distilled water. Samples were then diluted by a factor of 50 before PCR, owing to the high concentration of DNA in the samples. To facilitate detection of an *NIA1* and *Venus* deletion edits, we digested 2 µl of pooled genomic DNA samples with FastDigest HindIII (*NIA1*) or PstI (*Venus*) (Thermo Fisher) enzymes targeting the respective wild-type (non-edited) genomic DNA for 1 h at 37 °C in a 10 µl reaction using 1 µl (10 U) of enzyme following the manufacturer’s protocol. 2 µL of the digested sample was used for PCR using *NIA Edit Detection FP* / *RP* or *Venus Edit Detection FP / RP* primers as described above. PCR results from samples were then scored for the presence or absence of the edited *NIA1* or *Venus* gene (Extended Data Figs 3 and 5), and partially confirmed by Sanger sequencing (Fig. 4c and Extended Data Fig. 6b). This information was then used to calculate the approximate minimal number of edited seedlings in the F1 generation as shown in Fig. 4d, Supplementary Tables 1 and 2, Extended Data Fig. 6c.

### Reporting summary

Further information on research design is available in the Nature Portfolio Reporting Summary linked to this article.

## Online content

Any methods, additional references, Nature Portfolio reporting summaries, source data, extended data, supplementary information, acknowledgements, peer review information; details of author contributions and competing interests; and statements of data and code availability are available at 10.1038/s41587-022-01585-8.

## Supplementary information

Supplementary Information Supplementary Tables 1–5 and Supplementary Data 1.

Reporting Summary

## Data Availability

All data generated or analyzed during this study are included in this published article (and its supplementary information). Source data are provided with this paper.

## Source data

Source Data Fig. 2 Unprocessed gels.
Source Data Fig. 3 Unprocessed gels.
Source Data Fig. 4 Unprocessed gels.
Source Data Fig. 5 Unprocessed gels.
Source Data Extended Data Fig. 1 Unprocessed gels.
Source Data Extended Data Fig. 2 Unprocessed gels.
Source Data Extended Data Fig. 3 Unprocessed gels.
Source Data Extended Data Fig. 4 Unprocessed gels.
Source Data Extended Data Fig. 5 Unprocessed gels.
Source Data Extended Data Fig. 6 Unprocessed gels.
Source Data Extended Data Fig. 7 Unprocessed gels.
