# Supplementary material for: Heritable transgene-free genome editing in plants by grafting of wild-type shoots to transgenic donor rootstocks
Source: Nat Biotechnol. 2023 Jan 2;41(7):958–67. doi: 10.1038/s41587-022-01585-8 (PMC10344777; doi:10.1038/s41587-022-01585-8)
Supplement: Supplementary file 1 — Supplementary Tables 1–5 and Supplementary Data 1. [file 41587_2022_1585_MOESM1_ESM.pdf]

nature biotechnology

Article

https://doi.org/10.1038/s41587-022-01585-8

Heritable transgene-free genome editing in plants by grafting of wild-type shoots to transgenic donor rootstocks

In the format provided by the authors and unedited

Supplementary Information

Heritable transgene-free genome editing in plants by grafting of wild-type shoots to transgenic donor rootstock

Authors: Lei Yang[1,2], Frank Machin[1,2], Shuangfeng Wang[1], Eleftheria Saplaoura[1] and Friedrich Kragler[1]*

[1] Max Planck Institute of Molecular Plant Physiology; Am Mühlenberg 1, 14476 Potsdam, Germany

[2] These authors contributed equally: Lei Yang, Frank Machin.

* Corresponding author. Email: Kragler@mpimp-golm.mpg.de

This file includes:

Supplementary Table 1

Supplementary Table 2

Supplementary Table 3

Supplementary Table 4

Supplementary Table 5

Supplementary Data 1

Supplementary Table 1 | Number of *NIA1* Genome Edits Detected in the Transgene-free Offspring.

Note that each tested pool consisted of genomes isolated from ~70 to ~100 seedlings from a grafted parent plant indicated by the line #. Presence of edits (deletions) was confirmed by additional PCR assays and by Sanger sequencing of positive PCR amplicons (see Fig. 4c and Extended Data Fig. 3).

Graft Combination (scion/rootstock)	Grafted line # from which seedlings originated	Pools of seedlings tested	Edit (deletion) detected in a pool	Total number of seedlings tested	Minimal editing frequency per 1000 seedlings
Col-0 / *Cas9* x *gNIA1*	1	2	0	198	0
	2	1	0	255	0
	3	1	0	119	0
	4	2	0	119	0
	5	2	0	255	0
	6	1	0	153	0
	7	1	0	323	0
	8	1	0	221	0
	9	2	0	220	0
	10	1	0	238	0
	11	2	0	178	0
Total	11	16	0	2280	0
Col-0 / *Cas9-TLS1* x *gNIA1-TLS1*	1	2	1	143	7.0
	2	2	2	154	13.0
	3	3	1	242	4.1
	4	3	3	199	15.1
	5	2	0	110	0
	6	3	1	220	4.6
	7	3	2	264	7.6
	8	2	1	132	7.6
	9	2	1	198	5.1
	10	2	2	198	10.1
	11	1	1	77	13.0
	12	1	0	308	0

	13	2	0	242	0
	14	4	2	286	7.0
	15	1	0	198	0
Total	15	33	17	2971	5.7
Col-0 / *Cas9-TLS2* x *gNIA1-TLS2*	1	2	1	352	2.8
	2	2	0	270	0
	3	3	0	299	0
	4	3	2	396	5.1
	5	3	1	330	3.0
	6	5	0	462	0
	7	4	1	440	2.3
	8	5	3	385	7.8
	9	6	5	529	9.5
	10	3	3	484	6.2
	11	2	2	294	6.8
	12	5	3	360	8.3
	13	5	3	252	11.9
	14	4	3	360	8.3
	15	4	2	360	5.6
	16	4	3	378	7.9
	17	3	3	410	7.3
	18	5	4	504	7.9
	19	4	3	418	7.2
	20	2	0	520	0
	21	2	2	526	3.8
	22	3	0	437	0
Total	22	79	44	8766	5.0

Supplementary Table 2 | Number of *Venus* Genome Edits Detected in the Transgene-free Offspring.

Note that each tested pool consisted of genomes isolated from ~ 40 seedlings from a grafted parent plant indicated by the line #. Presence of edits (deletions) was confirmed by additional PCR assays and by Sanger sequencing of positive PCR amplicons (see Extended Data Fig. 4d and Extended Data Fig. 5).

Graft Combination (scion/rootstock)	Grafted line # from which seedlings originated	Pools of seedlings tested	Edit (deletion) detected in a pool	Total number of seedlings tested	Minimal editing frequency per 1000 seedlings
Col-0 / *Cas9* x *gVenus*	1	4	0	167	0
	2	4	0	138	0
	3	4	0	156	0
	4	4	0	145	0
	5	3	0	116	0
	6	4	0	157	0
	7	3	0	125	0
	8	4	0	177	0
	9	4	0	184	0
	10	4	0	158	0
	11	3	0	123	0
	12	4	0	139	0
	13	4	0	173	0
	14	4	0	164	0
	15	4	0	137	0
	16	4	0	173	0
	17	4	0	166	0
	18	4	0	184	0
	19	4	0	156	0
Total	19	76	0	2938	0
Col-0 / *Cas9-TLS1* x *gVenus-TLS1*	1	6	4	234	17.1
	2	5	3	205	14.6
	3	5	4	212	18.9
	4	6	6	243	24.7
	5	6	3	233	12.9

	6	5	4	198	20.2
	7	5	3	203	14.8
	8	5	2	189	10.6
	9	5	4	198	20.2
	10	6	0	226	0
	11	6	4	246	16.3
	12	5	2	208	9.6
	13	5	3	202	14.9
	14	6	1	226	4.4
	15	6	1	238	4.2
	16	5	4	199	20.1
	17	5	3	188	16.0
	18	6	4	247	16.2
	19	6	6	238	25.2
	20	6	4	234	17.1
Total	20	110	65	4367	14.9
Col-0 / *Cas9-TLS2* x *gVenus-TLS2*	1	5	2	205	9.8
	2	5	4	212	18.9
	3	6	4	224	17.9
	4	3	2	116	17.2
	5	5	5	185	27.0
	6	5	3	193	15.5
	7	4	2	147	13.6
	8	5	3	237	12.7
	9	6	4	247	16.2
	10	5	4	233	17.2
	11	5	2	199	10.1
	12	6	4	226	17.7
	13	6	2	244	8.2
	14	7	0	276	0
	15	6	5	243	20.6
	16	6	5	238	21.0
	17	7	5	277	18.1
	18	7	7	268	26.1
Total	18	99	63	3970	15.9

Supplementary Table 3 | Primer Sequences Used in the Study.

Primer	Sequence	Expected Product Size (bp)
*rbcsT*_FP	GCGGGTCTCGTCGAGAGAGCTTTCGTTCGTATCATCGGTTTCGAC	
*rbcsT*_RP	GCGGGTCTCCTCGACGTTGTCAATCAATTGGCAAGTCATAAAATGCATTAAAAAATATTTTCATA	665
Cas9 FP	TCGATGAGACCATGGATTACAAGGACCACGACGGGGATTACAAGG	
Cas9 RP	GCGTCGATGAGACCTCACTTCTTCTTCTTCGCCTGC	4276
Cas9-tRNA^{Met} RP	GCGGGTCTCCTCGATATCAGAGCCAGGTTTCGATCCTGGGACCTGTGGGTTATGGGCCCACCACGCTTCCGCTGCGCCACTCTGATAATTCACTTCTTCTTCTTCGCCTGCCCCGCCTTCTT	4351
Cas9-tRNA$^{Met\ \Delta DT}$ *RP*	GCGGGTCTCCTCGATATCAGAGCGGACCTGTGGGTTATGGGCCCACCACCACTCTGATAATTCACTTCTTCTTCTTCGCCTGCCCCGCCTTCTT	4323
NIA1 Fragment2 FP /<br>*Venus* Fragment2 FP	CGCGGTCTCCATTGACAACACTGCTGACTCTGCAGTTTTAGAGCTAGAAATAGC	*gRNA* Null: 621<br>*gRNA tRNAMet*: 704
NIA1 Fragment2 RP /<br>*Venus* Fragment2 FP	GCGGGTCTCTAAACCCTTCACGTTGTAACCCATCAATCTCTTAGTCGACTCTACC	gRNA *tRNAMetDT*: 676
NIA1 Edit Detection FP	AGGAGCGTCAGCTTGAGATT	Wild-type *NIA1*: 1469
NIA1 Edit Detection RP	GTTGTGACATGAGTCCTGACATGC	Edited *NIA1*: ~430
Cas9 RT-FP	AAGTCCAAGAAGCTCAAGAGC	
Cas9 RT-RP	GAAGTTCACATACTTGGACGGC	249
gRNA RT-FP	GTTTTAGAGCTAGAAATAGCAAG	
gRNA RT-RP	GCACCGACTCGGTGCCAC	76
Hyg RT-FP	ATGAAAAAGCCTGAACTCACC	
Hyg RT-RP	GCTGAAAGCACGAGATTCTTC	120
Kan RT-FP	AGAGGCTATTCGGCTATGACTGG	
Kan RT-RP	ATCGCCATGGGTCACGACGAGAT	450
ACTIN2-FP	GGAAGGATCTGTACGGTAAC	
ACTIN2-RP	TGTGAACGATTCCTGGACCT	245
UBQ10 qRT-FP	CACACTTCACTTGGTCTTGCGT	
UBQ10 qRT-RP	TAGTCTTTCCGGTGAGAGTCTTCA	71
Cas9 qRT-FP	AAGTCCAAGAAGCTCAAGAGC	
Cas9 qRT-RP	GAAGTTCACATACTTGGACGGC	249
Venus Edit Detection FP	TATCCTTCGCAAGACCCTTCCTC	Wild-type Venus: 1719
Venus Edit Detection RP	CGAGGTCGTCCGTCCACTC	Edited *Venus*: ~250

FP: Forward primer; RP: Reverse primer

Supplementary Table 4 | Binary *Cas9* and *gNIA1* Plasmids Created in the Study.

Plasmid	Function	Resistance Bacteria	Resistance Plants
pMDC7_*Cas9*	Estradiol-inducible expression of *Cas9*	Spectinomycin	Hygromycin
pMDC7_*Cas9-tRNA*^{Met}	Estradiol-inducible expression of *Cas9-TLS1*	Spectinomycin	Hygromycin
pMDC7_*Cas9-tRNA*$^{Met\ \Delta DT}$	Estradiol-inducible expression of *Cas9-TLS2*	Spectinomycin	Hygromycin
pMDC100_*gNIA1*	Constitutive expression of 2 *gRNAs* targeting *NIA1*	Kanamycin	Kanamycin
pMDC100_ *gNIA1-tRNA*^{Met}	Constitutive expression of 2 *gRNA-TLS1* targeting *NIA1*	Kanamycin	Kanamycin
pMDC100_ *gNIA1-tRNA*$^{Met\ \Delta DT}$	Constitutive expression of 2 *gRNA-TLS2* targeting *NIA1*	Kanamycin	Kanamycin
pMDC100_*gVenus*	Constitutive expression of 2 *gRNAs* targeting *Venus*	Kanamycin	Kanamycin
pMDC100_ *gVenus-tRNA*^{Met}	Constitutive expression of 2 *gRNA-TLS1* targeting *Venus*	Kanamycin	Kanamycin
pMDC100_ *gVenus-tRNA*$^{Met\ \Delta DT}$	Constitutive expression of 2 *gRNA-TLS2* targeting *Venus*	Kanamycin	Kanamycin
pRI909 35S::H2B-Venus_Basta			

Supplementary Table 5 | Nutrient Composition of the MS Growth Medium.

Component	$+NH_4$ Medium	$-NH_4$ Medium
NH_4NO_3	2.5mM	0
KNO_3	2.5mM	5 mM
$MgSO_4$	2 mM	2 mM
$CaCl_2$	1 mM	1 mM
MES (pH 5.8)	1.25 mM	1.25 mM
H_3BO_3	50 µM	50 µM
$MnSO_4$	12 µM	12 µM
$ZnCl_2$	1 µM	1 µM
$CuSO_4$	1 µM	1 µM
Na_2MoO_4	0.2 µM	0.2 µM
Sucrose	0.5%	0.5%
Micro-agar	0.5%	0.5%

Supplementary Data 1

a, Sequences of the *Cas9*, *Cas9-tRNA*^{Met} *(TLS1)* and *Cas9-tRNA*$^{Met\ \Delta DT}$ *(TLS2)* constructs in pMDC binary vector used in the study.

Light Grey colour background indicates *pG10-90* promoter; Light blue colour background indicates *XVE* gene sequence; Green colour background indicates *Hygromycin* resistance gene sequence. Dark Grey colour background indicates *LexA-35S* minimal promoter. Yellow colour background indicates *Cas9* gene sequence. Pink colour background indicates *tRNA*^{Met} (*TLS1*) or *tRNA*$^{Met\ \Delta DT}$ (*TLS2*) sequences fused to *Cas9*.

1. pMDC_*Cas9*

```
GTTTACCCGCCAATATATCCTGTCAAACACTGATAGTTTAAACTGAAGGCGGGAAACGACAATCTGATCCAAGCTCAAGCTAAGCTTGCATGCCTGCAGGATA
TCGTGGATCCAAGCTTGCCACGTGCCGCCACGTGCCGCCACGTGCCGCCACGTGCCTCTAGAGGATCCATCTCCACTGACGTAAGGGATGACGCACAATCCCA
CTATCCTTCGCAAGACCCTTCCTCTATATAAGGAAGTTCATTTCATTTGGAGAGGACACGCTGGGATCCCCAATTCCGGGCGGAATGAAAGCGTTAACGGCCA
GGCAACAAGAGGTGTTTGATCTCATCCGTGATCACATCAGCCAGACAGGTATGCCGCCGACGCGTGCGGAAATCGCGCAGCGTTTGGGGTTCCGTTCCCCAAA
CGCGGCTGAAGAACATCTGAAGGCGCTGGCACGCAAAGGCGTTATTGAAATTGTTTCCGGCGCATCACGCGGGATTCGTCTGTTGCAGGAAGAGGAAGAAGGG
TTGCCGCTGGTAGGTCGTGTGGCTGCCGGTGAACCGTCGAGCGCCCCCCGACCGATGTCAGCCTGGGGACGAGCTCCACTTAGACGGCGAGGACGTGGCGA
TGGCGCATGCCGACGCGCTAGACGATTTCGATCTGGACATGTTGGGGGACGGGGATTCCCCGGGTCCGGGATTTACCCCCCACGACTCCGCCCCCTACGGCGC
TCTGGATATGGCCGACTTCGAGTTTGAGCAGATGTTTACCGATGCCCTTGGAATTGACGAGTACGGTGGGGATCCGTCTGCTGGAGACATGAGAGCTGCCAAC
CTTTGGCCAAGCCCGCTCATGATCAAACGCTCTAAGAAGAACAGCCTGGCCTTGTCCCTGACGGCCGACCAGATGGTCAGTGCCTTGTTGGATGCTGAGCCCC
CCATACTCTATTCCGAGTATGATCCTACCAGACCCTTCAGTGAAGCTTCGATGATGGGCTTACTGACCAACCTGGCAGACAGGGAGCTGGTTCACATGATCAA
CTGGGCGAAGAGGGTGCCAGGCTTTGTGGATTTGACCCTCCATGATCAGGTCCACCTTCTAGAATGTGCCTGGCTAGAGATCCTGATGATTGGTCTCGTCTGG
CGCTCCATGGAGCACCCAGTGAAGCTACTGTTTGCTCCTAACTTGCTCTTGGACAGGAACCAGGGAAAATGTGTAGAGGGCATGGTGGAGATCTTCGACATGC
TGCTGGCTACATCATCTCGGTTCCGCATGATGAATCTGCAGGGAGAGGAGTTTGTGTGCCTCAAATCTATTATTTTGCTTAATTCTGGAGTGTACACATTTCT
GTCCAGCACCCTGAAGTCTCTGGAAGAGAAGGACCATATCCACCGAGTCCTGGACAAGATCACAGACACTTTGATCCACCTGATGGCCAAGGCAGGCCTGACC
CTGCAGCAGCAGCACCAGCGGCTGGCCCAGCTCCTCCTCATCCTCTCCCACATCAGGCACATGAGTAACAAAGGCATGGAGCATCTGTACAGCATGAAGTGCA
AGAACGTGGTGCCCCTCTATGACCTGCTGCTGGAGATGCTGGACGCCCACCGCCTACATGCGCCCACTAGCCGTGGAGGGGCATCCGTGGAGGAGACGGACCA
AAGCCACTTGGCCACTGCGGGCTCTACTTCATCGCATTCCTTGCAAAAGTATTACATCACGGGGGAGGCAGAGGGTTTCCCTGCCACAGTCTGAGAGCTCCCT
GGCGAATTCCCAGAGATGTTAGCTGAAATCATCACTAATCAGATACCAAAATATTCAAATGGAAATATCAAAAAGCTTCTGTTTCATCAAAAATGACTCGACC
TAACTGAGTAAGCTAGCTTGTTCGAGTATTATGGCATTGGGAAAACTGTTTTTCTTGTACCATTTGTTGTGCTTGTAATTTACTGTGTTTTTTATTCGGTTTT
CGCTATCGAACTGTGAAATGGAAATGGATGGAGAAGAGTTAATGAATGATATGGTCCTTTTGTTCATTCTCAAATTAATATTATTTGTTTTTTCTCTTATTTG
TTGTGTGTTGAATTTGAAATTATAAGAGATATGCAAACATTTTGTTTTGAGTAAAAATGTGTCAAATCGTGGCCTCTAATGACCGAAGTTAATATGAGGAGTA
AAACATCCCAAACAAGCTTGGAAACTGAAGGCGGGAAACGACAATCTGATCATGAGCGGAGAATTAAGGGAGTCACGTTATGACCCCCGCCGATGACGCGGGA
CAAGCCGTTTTACGTTTGGAACTGACAGAACCGCAACGATTGAAGGAGCCACTCAGCCGCGGGTTTCTGGAGTTTAATGAGCTAAGCACATACGTCAGAAACC
ATTATTGCGCGTTCAAAAGTCGCCTAAGGTCACTATCAGCTAGCAAATATTTCTTGTCAAAAATGCTCCACTGACGTTCCATAAATTCCCCTCGGTATCCAAT
TAGAGTCTCATATTCACTCTCAATCCAAATAATCTGCACCGGATCCCCTAGAATGAAAAAGCCTGAACTCACCGCGACGTCTGTCGAGAAGTTTCTGATCGAA
AAGTTCGACAGCGTCTCCGACCTGATGCAGCTCTCGGAGGGCGAAGAATCTCGTGCTTTCAGCTTCGATGTAGGAGGGCGTGGATATGTCCTGCGGGTAAATA
GCTGCGCCGATGGTTTCTACAAAGATCGTTATGTTTATCGGCACTTTGCATCGGCCGCGCTCCCGATTCCGGAAGTGCTTGACATTGGGGAATTCAGCGAGAG
CCTGACCTATTGCATCTCCCGCCGTGCACAGGGTGTCACGTTGCAAGACCTGCCTGAAACCGAACTGCCCGCTGTTCTGCAGCCGGTCGCGGAGGCCATGGAT
GCGATCGCTGCGGCCGATCTTAGCCAGACGAGCGGGTTCGGCCCATTCGGACCGCAAGGAATCGGTCAATACACTACATGGCGTGATTTCATATGCGCGATTG
CTGATCCCCATGTGTATCACTGGCAAACTGTGATGGACGACACCGTCAGTGCGTCCGTCGCGCAGGCTCTCGATGAGCTGATGCTTTGGGCCGAGGACTGCCC
CGAAGTCCGGCACCTCGTGCACGCGGATTTCGGCTCCAACAATGTCCTGACGGACAATGGCCGCATAACAGCGGTCATTGACTGGAGCGAGGCGATGTTCGGG
GATTCCCAATACGAGGTCGCCAACATCTTCTTCTGGAGGCCGTGGTTGGCTTGTATGGAGCAGCAGACGCGCTACTTCGAGCGGAGGCATCCGGAGCTTGCAG
GATCGCCGCGGCTCCGGGCGTATATGCTCCGCATTGGTCTTGACCAACTCTATCAGAGCTTGGTTGACGGCAATTTCGATGATGCAGCTTGGGCGCAGGGTCG
ATGCGACGCAATCGTCCGATCCGGAGCCGGGACTGTCGGGCGTACACAAATCGCCCGCAGAAGCGCGGCCGTCTGGACCGATGGCTGTGTAGAAGTACTCGCC
GATAGTGGAAACCGACGCCCCAGCACTCGTCCGAGGGCAAAGGAATAGCGATCGTTCAAACATTTGGCAATAAAGTTTCTTAAGATTGAATCCTGTTGCCGGT
CTTGCGATGATTATCATATAATTTCTGTTGAATTACGTTAAGCATGTAATAATTAACATGTAATGCATGACGTTATTTATGAGATGGGTTTTTATGATTAGAG
TCCCGCAATTATACATTTAATACGCGATAGAAAACAAAATATAGCGCGCAAACTAGGATAAATTATCGCGCGCGGTGTCATCTATGTTACTAGATCGGGGAAT
TGATCCCCCCTCGACAGCTTGCATGCCAGCTTGGGCTGCAGGTCGAGGCTAAAAACTAATCGCATTATCATCCCCTCGACGTACTGTACATATAACCACTGG
TTTTATATACAGCAGTACTGTACATATAACCACTGGTTTTATATACAGCAGTCGACGTACTGTACATATAACCACTGGTTTTATATACAGCAGTACTGTACAT
ATAACCACTGGTTTTATATACAGCAGTCGAGGTAAGATTAGATATGGATATGTATATGGATATGTATATGGTGGTAATGCCATGTAATATGCTCGACTCTAGG
ATCTTCGCAAGACCCTTCCTCTATATAAGGAAGTTCATTTCATTTGGAGAGGACACGCTGAAGCTAGTCGACTCTAGCCTCGAATGGATTACAAGGACCACGA
CGGGGATTACAAGGACCACGACATTGATTACAAGGATGATGATGACAAGATGGCTCCGAAGAAGAAGAGGAAGGTTGGCATCCACGGGGTGCCAGCTGCTGAC
AAGAAGTACTCGATCGGCCTCGATATTGGGACTAACTCTGTTGGCTGGGCCGTGATCACCGACGAGTACAAGGTGCCCTCAAAGAAGTTCAAGGTCCTGGGCA
ACACCGATCGGCATTCCATCAAGAAGAATCTCATTGGCGCTCTCCTGTTCGACAGCGGCGAGACGGCTGAGGCTACGCGGCTCAAGCGCACCGCCCGCAGGCG
GTACACGCGCAGGAAGAATCGCATCTGCTACCTGCAGGAGATTTTCTCCAACGAGATGGCGAAGGTTGACGATTCTTTCTTCCACAGGCTGGAGGAGTCATTC
CTCGTGGAGGAGGATAAGAAGCACGAGCGGCATCCAATCTTCGGCAACATTGTCGACGAGGTTGCCTACCACGAGAAGTACCCTACGATCTACCATCTGCGGA
AGAAGCTCGTGGACTCCACAGATAAGGCGGACCTCCGCCTGATCTACCTCGCTCTGGCCCACATGATTAAGTTCAGGGGCCATTTCCTGATCGAGGGGGATCT
CAACCCGGACAATAGCGATGTTGACAAGCTGTTCATCCAGCTCGTGCAGACGTACAACCAGCTCTTCGAGGAGAACCCCATTAATGCGTCAGGCGTCGACGCG
AAGGCTATCCTGTCCGCTAGGCTCTCGAAGTCTCGGCGCCTCGAGAACCTGATCGCCCAGCTGCCGGGCGAGAAGAAGAACGGCCTGTTCGGGAATCTCATTG
CGCTCAGCCTGGGGCTCACGCCCAACTTCAAGTCGAATTTCGATCTCGCTGAGGACGCCAAGCTGCAGCTCTCCAAGGACACATACGACGATGACCTGGATAA
CCTCCTGGCCCAGATCGGCGATCAGTACGCGGACCTGTTCCTCGCTGCCAAGAATCTGTCGGACGCCATCCTCCTGTCTGATATTCTCAGGGTGAACACCGAG
ATTACGAAGGCTCCGCTCTCAGCCTCCATGATCAAGCGCTACGACGAGCACCATCAGGATCTGACCCTCCTGAAGGCGCTGGTCAGGCAGCAGCTCCCCGAGA
AGTACAAGGAGATCTTCTTCGATCAGTCGAAGAACGGCTACGCTGGGTACATTGACGGCGGGGCCTCTCAGGAGGAGTTCTACAAGTTCATCAAGCCGATTCT
GGAGAAGATGGACGGCACGGAGGAGCTGCTGGTGAAGCTCAATCGCGAGGACCTCCTGAGGAAGCAGCGGACATTCGATAACGGCAGCATCCCACACCAGATT
```

CATCTCGGGGAGCTGCACGCTATCCTGAGGAGGCAGGAGGACTTCTACCCTTTCCTCAAGGATAACCGCGAGAAGATCGAGAAGATTCTGACTTTCAGGATCC
CGTACTACGTCGGCCCACTCGCTAGGGGCAACTCCCGCTTCGCTTGGATGACCCGCAAGTCAGAGGAGACGATCACGCCGTGGAACTTCGAGGAGGTGGTCGA
CAAGGGCGCTAGCGCTCAGTCGTTCATCGAGAGGATGACGAATTTCGACAAGAACCTGCCAAATGAGAAGGTGCTCCCTAAGCACTCGCTCCTGTACGAGTAC
TTCACAGTCTACAACGAGCTGACTAAGGTGAAGTATGTGACCGAGGGCATGAGGAAGCCGGCTTTCCTGTCTGGGGAGCAGAAGAAGGCCATCGTGGACCTCC
TGTTCAAGACCAACCGGAAGGTCACGGTTAAGCAGCTCAAGGAGGACTACTTCAAGAAGATTGAGTGCTTCGATTCGGTCGAGATCTCTGGCGTTGAGGACCG
CTTCAACGCCTCCCTGGGGACCTACCACGATCTCCTGAAGATCATTAAGGATAAGGACTTCCTGGACAACGAGGAGAATGAGGATATCCTCGAGGACATTGTG
CTGACACTCACTCTGTTCGAGGACCGGGAGATGATCGAGGAGCGCCTGAAGACTTACGCCCATCTCTTCGATGACAAGGTCATGAAGCAGCTCAAGAGGAGGA
GGTACACCGGCTGGGGGAGGCTGAGCAGGAAGCTCATCAACGGCATTCGGGACAAGCAGTCCGGGAAGACGATCCTCGACTTCCTGAAGAGCGATGGCTTCGC
GAACCGCAATTTCATGCAGCTGATTCACGATGACAGCCTCACATTCAAGGAGGATATCCAGAAGGCTCAGGTGAGCGGCCAGGGGGACTCGCTGCACGAGCAT
ATCGCGAACCTCGCTGGCTCGCCAGCTATCAAGAAGGGGATTCTGCAGACCGTGAAGGTTGTGGACGAGCTGGTGAAGGTCATGGGCAGGCACAAGCCTGAGA
ACATCGTCATTGAGATGGCCCGGGAGAATCAGACCACGCAGAAGGGCCAGAAGAACTCACGCGAGAGGATGAAGAGGATCGAGGAGGGCATTAAGGAGCTGGG
GTCCCAGATCCTCAAGGAGCACCCGGTGGAGAACACGCAGCTGCAGAATGAGAAGCTCTACCTGTACTACCTCCAGAATGGCCGCGATATGTATGTGGACCAG
GAGCTGGATATTAACAGGCTCAGCGATTACGACGTCGATCATATCGTTCCACAGTCATTCCTGAAGGATGACTCCATTGACAACAAGGTCCTCACCAGGTCGG
ACAAGAACCGGGGCAAGTCTGATAATGTTCCTTCAGAGGAGGTCGTTAAGAAGATGAAGAACTACTGGCGCCAGCTCCTGAATGCCAAGCTGATCACGCAGCG
GAAGTTCGATAACCTCACAAAGGCTGAGAGGGGCGGGCTCTCTGAGCTGGACAAGGCGGGCTTCATCAAGAGGCAGCTGGTCGAGACACGGCAGATCACTAAG
CACGTTGCGCAGATTCTCGACTCACGGATGAACACTAAGTACGATGAGAATGACAAGCTGATCCGCGAGGTGAAGGTCATCACCCTGAAGTCAAAGCTCGTCT
CCGACTTCAGGAAGGATTTCCAGTTCTACAAGGTTCGGGAGATCAACAATTACCACCATGCCCATGACGCGTACCTGAACGCGGTGGTCGGCACAGCTCTGAT
CAAGAAGTACCCAAAGCTCGAGAGCGAGTTCGTGTACGGGGACTACAAGGTTTACGATGTGAGGAAGATGATCGCCAAGTCGGAGCAGGAGATTGGCAAGGCT
ACCGCCAAGTACTTCTTCTACTCTAACATTATGAATTTCTTCAAGACAGAGATCACTCTGGCCAATGGCGAGATCCGGAAGCGCCCCCTCATCGAGACGAACG
GCGAGACGGGGGAGATCGTGTGGGACAAGGGCAGGGATTTCGCGACCGTCAGGAAGGTTCTCTCCATGCCACAAGTGAATATCGTCAAGAAGACAGAGGTCCA
GACTGGCGGGTTCTCTAAGGAGTCAATTCTGCCTAAGCGGAACAGCGACAAGCTCATCGCCCGCAAGAAGGACTGGGATCCGAAGAAGTACGGCGGGTTCGAC
AGCCCCACTGTGGCCTACTCGGTCCTGGTTGTGGCGAAGGTTGAGAAGGGCAAGTCCAAGAAGCTCAAGAGCGTGAAGGAGCTGCTGGGGATCACGATTATGG
AGCGCTCCAGCTTCGAGAAGAACCCGATCGATTTCCTGGAGGCGAAGGGCTACAAGGAGGTGAAGAAGGACCTGATCATTAAGCTCCCCAAGTACTCACTCTT
CGAGCTGGAGAACGGCAGGAAGCGGATGCTGGCTTCCGCTGGCGAGCTGCAGAAGGGGAACGAGCTGGCTCTGCCGTCCAAGTATGTGAACTTCCTCTACCTG
GCCTCCCACTACGAGAAGCTCAAGGGCAGCCCCGAGGACAACGAGCAGAAGCAGCTGTTCGTCGAGCAGCACAAGCATTACCTCGACGAGATCATTGAGCAGA
TTTCCGAGTTCTCCAAGCGCGTGATCCTGGCCGACGCGAATCTGGATAAGGTCCTCTCCGCGTACAACAAGCACCGCGACAAGCCAATCAGGGAGCAGGCTGA
GAATATCATTCATCTCTTCACCCTGACGAACCTCGGCGCCCCTGCTGCTTTCAAGTACTTCGACACAACTATCGATCGCAAGAGGTACACAAGCACTAAGGAG
GTCCTGGACGCGACCCTCATCCACCAGTCGATTACCGGCCTCTACGAGACGCGCATCGACCTGTCTCAGCTCGGGGGCGACAAGCGGCCAGCGGCGACGAAGA
AGGCGGGGCAGGCGAAGAAGAAGAAG**TGA**TCGAGAGAGCTTTCGTTCGTATCATCGGTTTCGACAACGTTCGTCAAGTTCAATGCATCAGTTTCATTGCGCAC
ACACCAGAATCCTACTGAGTTTGAGTATTATGGCATTGGGAAAACTGTTTTTCTTGTACCATTTGTTGTGCTTGTAATTTACTGTGTTTTTTATTCGGTTTTC
GCTATCGAACTGTGAAATGGAAATGGATGGAGAAGAGTTAATGAATGATATGGTCCTTTTGTTCATTCTCAAATTAATATTATTTGTTTTTTCTCTTATTTGT
TGTGTGTTGAATTTGAAATTATAAGAGATATGCAAACATTTTGTTTTGAGTAAAAATGTGTCAAATCGTGGCCTCTAATGACCGAAGTTAATATGAGGAGTAA
AACACTTGTAGTTGTACCATTATGCTTATTCACTAGGCAACAAATATATTTTCAGACCTAGAAAAGCTGCAAATGTTACTGAATACAAGTATGTCCTCTTGTG
TTTTAGACATTTATGAACTTTCCTTTATGTAATTTTCCAGAATCCTTGTCAGATTCTAATCATTGCTTTATAATTATAGTTATACTCATGGATTTGTAGTTGA
GTATGAAAATATTTTTTAATGCATTTTATGACTTGCCAATTGATTGACAACGTCGAGGCGCGCCAAGCTATCAACAAGTTTGTACAAAAAAGCAGGCTCCACC
ATGGGAACCAATTCAGTCGACTGGATCCGGTACCGAATTCGCTTACTAAAAGCCAGATAACAGTATGCGTATTTGCGCGCTGATTTTTGCGGTATAAGAATAT
ATACTGATATGTATACCCGAAGTATGTCAAAAAGAGGTGTGCTTCTAGACCACTTTGTACAAGAAAGCTGGGTTGATAATTCTTAATTAACTAGTCGATCCAG
GCCTCCCAGCTTTCGTCCGTATCATCGGTTTCGACAACGTTCGTCAAGTTCAATGCATCAGTTTCATTGCCCACACACCAGAATCCTACTAAGTTTGAGTATT
ATGGCATTGGAAAAGCTGTTTTCTTCTATCATTTGTTCTGCTTGTAATTTACTGTGTTCTTTCAGTTTTTGTTTTCGGACATCAAAATGCAAATGGATGGATA
AGAGTTAATAAATGATATGGTCCTTTTGTTCATTCTCAAATTATTATTATCTGTTGTTTTTACTTTAATGGGTTGAATTTAAGTAAGAAAGGAACTAACAGTG
TGATATTAAGGTGCAATGTTAGACATATAAAACAGTCTTTCACCTCTCTTTGGTTATGTCTTGAATTGGTTTGTTTCTTCACTTATCTGTGTAATCAAGTTTA
CTATGAGTCTATGATCAAGTAATTATGCAATCAAGTTAAGTACAGTATAGGCTTTTTGTGTCGAGGGGGTACCGAGTCGAGGAATTCACTGGCCGTCGTTTTA
CAACGTCGTGACTGGGAAAACCCTGGCGTTACCCAACTTAATCGCCTTGCAGCACATCCCCCTTTCGCCAGCTGGCGTAATAGCGAAGAGGCCCGCACCGATC
GCCCTTCCCAACAGTTGCGCAGCCTGAATGGCGGGTACCGAGCTCGAATTCAATTCGGCGTTAATTCAGTACATTAAAAACGTCCGCAATGTGTTATTAAGTT
GTCTAAGCGTCAATTTGTTTACACCACAATATATCCTGCCACCAGCCAGCCAACAGCTCCCCGACCGGCAGCTCGGCACAAAATCACCACTCGATACAGGCAG
CCCATCAGTCCGGGACGGCGTCAGCGGGAGAGCCGTTGTAAGGCGGCAGACTTTGCTCATGTTACCGATGCTATTCGGAAGAACGGCAACTAAGCTGCCGGGT
TTGAAACACGGATGATCTCGCGGAGGGTAGCATGTTGATTGTAACGATGACAGAGCGTTGCTGCCTGTGATCAATTCGGGCACGAACCCAGTGGACATAAGCC
TCGTTCGGTTCGTAAGCTGTAATGCAAGTAGCGTAACTGCCGTCACGCAACTGGTCCAGAACCTTGACCGAACGCAGCGGTGGTAACGGCGCAGTGGCGGTTT
TCATGGCTTCTTGTTATGACATGTTTTTTTGGGGTACAGTCTATGCCTCGGGCATCCAAGCAGCAAGCGCGTTACGCCGTGGGTCGATGTTTGATGTTATGGA
GCAGCAACGATGTTACGCAGCAGGGCAGTCGCCCTAAAACAAAGTTAAACATCATGGGGGAAGCGGTGATCGCCGAAGTATCGACTCAACTATCAGAGGTAGT
TGGCGTCATCGAGCGCCATCTCGAACCGACGTTGCTGGCCGTACATTTGTACGGCTCCGCAGTGGATGGCGGCCTGAAGCCACACAGTGATATTGATTTGCTG
GTTACGGTGACCGTAAGGCTTGATGAAACAACGCGGCGAGCTTTGATCAACGACCTTTTGGAAACTTCGGCTTCCCCTGGAGAGAGCGAGATTCTCCGCGCTG
TAGAAGTCACCATTGTTGTGCACGACGACATCATTCCGTGGCGTTATCCAGCTAAGCGCGAACTGCAATTTGGAGAATGGCAGCGCAATGACATTCTTGCAGG
TATCTTCGAGCCAGCCACGATCGACATTGATCTGGCTATCTTGCTGACAAAAGCAAGAGAACATAGCGTTGCCTTGGTAGGTCCAGCGGCGGAGGAACTCTTT
GATCCGGTTCCTGAACAGGATCTATTTGAGGCGCTAAATGAAACCTTAACGCTATGGAACTCGCCGCCCGACTGGGCTGGCGATGAGCGAAATGTAGTGCTTA
CGTTGTCCCGCATTTGGTACAGCGCAGTAACCGGCAAAATCGCGCCGAAGGATGTCGCTGCCGACTGGGCAATGGAGCGCCTGCCGGCCCAGTATCAGCCCGT
CATACTTGAAGCTAGACAGGCTTATCTTGGACAAGAAGAAGATCGCTTGGCCTCGCGCGCAGATCAGTTGGAAGAATTTGTCCACTACGTGAAAGGCGAGATC
ACCAAGGTAGTCGGCAAATAATGTCTAGCTAGAAATTCGTTCAAGCCGACGCCGCTTCGCCGGCGTTAACTCAAGCGATTAGATGCACTAAGCACATAATTGC
TCACAGCCAAACTATCAGGTCAAGTCTGCTTTTATTATTTTTAAGCGTGCATAATAAGCCCTACACAAATTGGGAGATATATCATGCATGACCAAAATCCCTT
AACGTGAGTTTTCGTTCCACTGAGCGTCAGACCCCGTAGAAAAGATCAAAGGATCTTCTTGAGATCCTTTTTTTCTGCGCGTAATCTGCTGCTTGCAAACAAA
AAAACCACCGCTACCAGCGGTGGTTTGTTTGCCGGATCAAGAGCTACCAACTCTTTTTCCGAAGGTAACTGGCTTCAGCAGAGCGCAGATACCAAATACTGTC
CTTCTAGTGTAGCCGTAGTTAGGCCACCACTTCAAGAACTCTGTAGCACCGCCTACATACCTCGCTCTGCTAATCCTGTTACCAGTGGCTGCTGCCAGTGGCG
ATAAGTCGTGTCTTACCGGGTTGGACTCAAGACGATAGTTACCGGATAAGGCGCAGCGGTCGGGCTGAACGGGGGGTTCGTGCACACAGCCCAGCTTGGAGCG
AACGACCTACACCGAACTGAGATACCTACAGCGTGAGCTATGAGAAAGCGCCACGCTTCCCGAAGGGAGAAAGGCGGACAGGTATCCGGTAAGCGGCAGGGTC
GGAACAGGAGAGCGCACGAGGGAGCTTCCAGGGGGAAACGCCTGGTATCTTTATAGTCCTGTCGGGTTTCGCCACCTCTGACTTGAGCGTCGATTTTTGTGAT
GCTCGTCAGGGGGGCGGAGCCTATGGAAAAACGCCAGCAACGCGGCCTTTTTACGGTTCCTGGCCTTTTGCTGGCCTTTTGCTCACATGTTCTTTCCTGCGTT
ATCCCCTGATTCTGTGGATAACCGTATTACCGCCTTTGAGTGAGCTGATACCGCTCGCCGCAGCCGAACGACCGAGCGCAGCGAGTCAGTGAGCGAGGAAGCG
GAAGAGCGCCTGATGCGGTATTTTCTCCTTACGCATCTGTGCGGTATTTCACACCGCATATGGTGCACTCTCAGTACAATCTGCTCTGATGCCGCATAGTTAA
GCCAGTATACACTCCGCTATCGCTACGTGACTGGGTCATGGCTGCGCCCCGACACCCGCCAACACCCGCTGACGCGCCCTGACGGGCTTGTCTGCTCCCGGCA
TCCGCTTACAGACAAGCTGTGACCGTCTCCGGGAGCTGCATGTGTCAGAGGTTTTCACCGTCATCACCGAAACGCGCGAGGCAGGGTGCCTTGATGTGGGCGC
CGGCGGTCGAGTGGCGACGGCGCGGCTTGTCCGCGCCCTGGTAGATTGCCTGGCCGTAGGCCAGCCATTTTTGAGCGGCCAGCGGCCGCGATAGGCCGACGCG
AAGCGGCGGGGCGTAGGGAGCGCAGCGACCGAAGGGTAGGCGCTTTTTGCAGCTCTTCGGCTGTGCGCTGGCCAGACAGTTATGCACAGGCCAGGCGGGTTTT
AAGAGTTTTAATAAGTTTTAAAGAGTTTTAGGCGGAAAAATCGCCTTTTTTCTCTTTTATATCAGTCACTTACATGTGTGACCGGTTCCCAATGTACGGCTTT
GGGTTCCCAATGTACGGGTTCCGGTTCCCAATGTACGGCTTTGGGTTCCCAATGTACGTGCTATCCACAGGAAAGAGACCTTTTCGACCTTTTTCCCCTGCTA
GGGCAATTTGCCCTAGCATCTGCTCCGTACATTAGGAACCGGCGGATGCTTCGCCCTCGATCAGGTTGCGGTAGCGCATGACTAGGATCGGGCCAGCCTGCCC
CGCCTCCTCCTTCAAATCGTACTCCGGCAGGTCATTTGACCCGATCAGCTTGCGCACGGTGAAACAGAACTTCTTGAACTCTCCGGCGCTGCCACTGCGTTCG
TAGATCGTCTTGAACAACCATCTGGCTTCTGCCTTGCCTGCGGCGCGGCGTGCCAGGCGGTAGAGAAAACGGCCGATGCCGGGATCGATCAAAAAGTAATCGG
GGTGAACCGTCAGCACGTCCGGGTTCTTGCCTTCTGTGATCTCGCGGTACATCCAATCAGCTAGCTCGATCTCGATGTACTCCGGCCGCCCGGTTTCGCTCTT
TACGATCTTGTAGCGGCTAATCAAGGCTTCACCCTCGGATACCGTCACCAGGCGGCCGTTCTTGGCCTTCTTCGTACGCTGCATGGCAACGTGCGTGGTGTTT

AACCGAATGCAGGTTTCTACCAGGTCGTCTTTCTGCTTTCCGCCATCGGCTCGCCGGCAGAACTTGAGTACGTCCGCAACGTGTGGACGGAACACGCGGCCGG
GCTTGTCTCCCTTCCCTTCCCGGTATCGGTTCATGGATTCGGTTAGATGGGAAACCGCCATCAGTACCAGGTCGTAATCCCACACACTGGCCATGCCGGCCGG
CCCTGCGGAAACCTCTACGTGCCCGTCTGGAAGCTCGTAGCGGATCACCTCGCCAGCTCGTCGGTCACGCTTCGACAGACGGAAAACGGCCACGTCCATGATG
CTGCGACTATCGCGGGTGCCCACGTCATAGAGCATCGGAACGAAAAAATCTGGTTGCTCGTCGCCCTTGGGCGGCTTCCTAATCGACGGCGCACCGGCTGCCG
GCGGTTGCCGGGATTCTTTGCGGATTCGATCAGCGGCCGCTTGCCACGATTCACCGGGGCGTGCTTCTGCCTCGATGCGTTGCCGCTGGGCGGCCTGCGCGGC
CTTCAACTTCTCCACCAGGTCATCACCCAGCGCCGCGCCGATTTGTACCGGGCCGGATGGTTTGCGACCGTCACGCCGATTCCTCGGGCTTGGGGGTTCCAGT
GCCATTGCAGGGCCGGCAGACAACCCAGCCGCTTACGCCTGGCCAACCGCCCGTTCCTCCACACATGGGGCATTCCACGGCGTCGGTGCCTGGTTGTTCTTGA
TTTTCCATGCCGCCTCCTTTAGCCGCTAAAATTCATCTACTCATTTATTCATTTGCTCATTTACTCTGGTAGCTGCGCGATGTATTCAGATAGCAGCTCGGTA
ATGGTCTTGCCTTGGCGTACCGCGTACATCTTCAGCTTGGTGTGATCCTCCGCCGGCAACTGAAAGTTGACCCGCTTCATGGCTGGCGTGTCTGCCAGGCTGG
CCAACGTTGCAGCCTTGCTGCTGCGTGCGCTCGGACGGCCGGCACTTAGCGTGTTTGTGCTTTTGCTCATTTTCTCTTTACCTCATTAACTCAAATGAGTTTT
GATTTAATTTCAGCGGCCAGCGCCTGGACCTCGCGGGCAGCGTCGCCCTCGGGTTCTGATTCAAGAACGGTTGTGCCGGCGGCGGCAGTGCCTGGGTAGCTCA
CGCGCTGCGTGATACGGGACTCAAGAATGGGCAGCTCGTACCCGGCCAGCGCCTCGGCAACCTCACCGCCGATGCGCGTGCCTTTGATCGCCCGCGACACGAC
AAAGGCCGCTTGTAGCCTTCCATCCGTGACCTCAATGCGCTGCTTAACCAGCTCCACCAGGTCGGCGGTGGCCCATATGTCGTAAGGGCTTGGCTGCACCGGA
ATCAGCACGAAGTCGGCTGCCTTGATCGCGGACACAGCCAAGTCCGCCGCCTGGGGCGCTCCGTCGATCACTACGAAGTCGCGCCGGCCGATGGCCTTCACGT
CGCGGTCAATCGTCGGGCGGTCGATGCCGACAACGGTTAGCGGTTGATCTTCCCGCACGGCCGCCCAATCGCGGGCACTGCCCTGGGGATCGGAATCGACTAA
CAGAACATCGGCCCCGGCGAGTTGCAGGGCGCGGGCTAGATGGGTTGCGATGGTCGTCTTGCCTGACCCGCCTTTCTGGTTAAGTACAGCGATAACCTTCATG
CGTTCCCCTTGCGTATTTGTTTATTTACTCATCGCATCATATACGCAGCGACCGCATGACGCAAGCTGTTTTACTCAAATACACATCACCTTTTTAGACGGCG
GCGCTCGGTTTCTTCAGCGGCCAAGCTGGCCGGCCAGGCCGCCAGCTTGGCATCAGACAAACCGGCCAGGATTTCATGCAGCCGCACGGTTGAGACGTGCGCG
GGCGGCTCGAACACGTACCCGGCCGCGATCATCTCCGCCTCGATCTCTTCGGTAATGAAAAACGGTTCGTCCTGGCCGTCCTGGTGCGGTTTCATGCTTGTTC
CTCTTGGCGTTCATTCTCGGCGGCCGCCAGGGCGTCGGCCTCGGTCAATGCGTCCTCACGGAAGGCACCGCGCCGCCTGGCCTCGGTGGGCGTCACTTCCTCG
CTGCGCTCAAGTGCGCGGTACAGGGTCGAGCGATGCACGCCAAGCAGTGCAGCCGCCTCTTTCACGGTGCGGCCTTCCTGGTCGATCAGCTCGCGGGCGTGCG
CGATCTGTGCCGGGGTGAGGGTAGGGCGGGGGCCAAACTTCACGCCTCGGGCCTTGGCGGCCTCGCGCCCGCTCCGGGTGCGGTCGATGATTAGGGAACGCTC
GAACTCGGCAATGCCGGCGAACACGGTCAACACCATGCGGCCGGCCGGCGTGGTGGTGTCGGCCCACGGCTCTGCCAGGCTACGCAGGCCCGCGCCGGCCTCC
TGGATGCGCTCGGCAATGTCCAGTAGGTCGCGGGTGCTGCGGGCCAGGCGGTCTAGCCTGGTCACTGTCACAACGTCGCCAGGGCGTAGGTGGTCAAGCATCC
TGGCCAGCTCCGGGCGGTCGCGCCTGGTGCCGGTGATCTTCTCGGAAAACAGCTTGGTGCAGCCGGCCGCGTGCAGTTCGGCCCGTTGGTTGGTCAAGTCCTG
GTCGTCGGTGCTGACGCGGGCATAGCCCAGCAGGCCAGCGGCGGCGCTCTTGTTCATGGCGTAATGTCTCCGGTTCTAGTCGCAAGTATTCTACTTTATGCGA
CTAAAACACGCGACAAGAAAACGCCAGGAAAAGGGCAGGGCGGCAGCCTGTCGCGTAACTTAGGACTTGTGCGACATGTCGTTTTCAGAAGACGGCTGCACTG
AACGTCAGAAGCCGACTGCACTATAGCAGCGGAGGGGTTGGATCAAAGTACTTTGATCCCGAGGGGAACCCTGTGGTTGGCATGCACATACAAATGGACGAAC
GGATAAACCTTTTCACGCCCTTTTAAATATCCGTTATTCTAATAAACGCTCTTTTCTCTTAG

2. *Cas9-tRNA^{Met} (TLS1)*

GTTTACCCGCCAATATATCCTGTCAAACACTGATAGTTTAAACTGAAGGCGGGAAACGACAATCTGATCCAAGCTCAAGCTAAGCTTGCATGCCTGCAGGATA
TCGTGGATCCAAGCTTGCCACGTGCCGCCACGTGCCGCCACGTGCCGCCACGTGCCTCTAGAGGATCCATCTCCACTGACGTAAGGGATGACGCACAATCCCA
CTATCCTTCGCAAGACCCTTCCTCTATATAAGGAAGTTCATTTCATTTGGAGAGGACACGCTGGGATCCCCAATTCCGGGCGGAATGAAAGCGTTAACGGCCA
GGCAACAAGAGGTGTTTGATCTCATCCGTGA……………………….TGCATGCCAGCTTGGGCTGCAGGTCGAGGCTAAAAACTAATCGCATTATCATCCCCTCG
ACGTACTGTACATATAACCACTGGTTTTATATACAGCAGTACTGTACATATAACCACTGGTTTTATATACAGCAGTCGACGTACTGTACATATAACCACTGGT
TTTATATACAGCAGTACTGTACATATAACCACTGGTTTTATATACAGCAGTCGAGGTAAGATTAGATATGGATATGTATATGGATATGTATATGGTGGTAATG
CCATGTAATATGCTCGACTCTAGGATCTTCGCAAGACCCTTCCTCTATATAAGGAAGTTCATTTCATTTGGAGAGGACACGCTGAAGCTAGTCGACTCTAGCC
TCGAATGGATTACAAGGACCACGACGGGGATTACAAGGACCACGACATTGATTACAAGGATGATGATGACAAGATGGCTCCGAAGAAGAAGAGGAAGGTTGGC
ATCCACGGGGTGCCAGCTGCTGACAAGAAGTACTCGATCGGCCTCGATATTGGGACTAACTCTGTTGGCTGGGCCGTGATCACCGACGAGTACAAGGTGCCCT
CAAAGAAGTTCAAGGTCCTGGGCAACACCGATCGGCATTCCATCAAGAAGAATCTCATTGGCGCTCTCCTGTTCGACAGCGGCGAGACGGCTGAGGCTACGCG
GCTCAAGCGCACCGCCCGCAGGCGGTACACGCGCAGGAAGAATCGCATCTGCTACCTGCAGGAGATTTTCTCCAACGAGATGGCGAAGGTTGACGATTCTTTC
TTCCACAGGCTGGAGGAGTCATTCCTCGTGGAGGAGGATAAGAAGCACGAGCGGCATCCAATCTTCGGCAACATTGTCGACGAGGTTGCCTACCACGAGAAGT
ACCCTACGATCTACCATCTGCGGAAGAAGCTCGTGGACTCCACAGATAAGGCGGACCTCCGCCTGATCTACCTCGCTCTGGCCCACATGATTAAGTTCAGGGG
CCATTTCCTGATCGAGGGGGATCTCAACCCGGACAATAGCGATGTTGACAAGCTGTTCATCCAGCTCGTGCAGACGTACAACCAGCTCTTCGAGGAGAACCCC
ATTAATGCGTCAGGCGTCGACGCGAAGGCTATCCTGTCCGCTAGGCTCTCGAAGTCTCGGCGCCTCGAGAACCTGATCGCCCAGCTGCCGGGCGAGAAGAAGA
ACGGCCTGTTCGGGAATCTCATTGCGCTCAGCCTGGGGCTCACGCCCAACTTCAAGTCGAATTTCGATCTCGCTGAGGACGCCAAGCTGCAGCTCTCCAAGGA
CACATACGACGATGACCTGGATAACCTCCTGGCCCAGATCGGCGATCAGTACGCGGACCTGTTCCTCGCTGCCAAGAATCTGTCGGACGCCATCCTCCTGTCT
GATATTCTCAGGGTGAACACCGAGATTACGAAGGCTCCGCTCTCAGCCTCCATGATCAAGCGCTACGACGAGCACCATCAGGATCTGACCCTCCTGAAGGCGC
TGGTCAGGCAGCAGCTCCCCGAGAAGTACAAGGAGATCTTCTTCGATCAGTCGAAGAACGGCTACGCTGGGTACATTGACGGCGGGGCCTCTCAGGAGGAGTT
CTACAAGTTCATCAAGCCGATTCTGGAGAAGATGGACGGCACGGAGGAGCTGCTGGTGAAGCTCAATCGCGAGGACCTCCTGAGGAAGCAGCGGACATTCGAT
AACGGCAGCATCCCACACCAGATTCATCTCGGGGAGCTGCACGCTATCCTGAGGAGGCAGGAGGACTTCTACCCTTTCCTCAAGGATAACCGCGAGAAGATCG
AGAAGATTCTGACTTTCAGGATCCCGTACTACGTCGGCCCACTCGCTAGGGGCAACTCCCGCTTCGCTTGGATGACCCGCAAGTCAGAGGAGACGATCACGCC
GTGGAACTTCGAGGAGGTGGTCGACAAGGGCGCTAGCGCTCAGTCGTTCATCGAGAGGATGACGAATTTCGACAAGAACCTGCCAAATGAGAAGGTGCTCCCT
AAGCACTCGCTCCTGTACGAGTACTTCACAGTCTACAACGAGCTGACTAAGGTGAAGTATGTGACCGAGGGCATGAGGAAGCCGGCTTTCCTGTCTGGGGAGC
AGAAGAAGGCCATCGTGGACCTCCTGTTCAAGACCAACCGGAAGGTCACGGTTAAGCAGCTCAAGGAGGACTACTTCAAGAAGATTGAGTGCTTCGATTCGGT
CGAGATCTCTGGCGTTGAGGACCGCTTCAACGCCTCCCTGGGGACCTACCACGATCTCCTGAAGATCATTAAGGATAAGGACTTCCTGGACAACGAGGAGAAT
GAGGATATCCTCGAGGACATTGTGCTGACACTCACTCTGTTCGAGGACCGGGAGATGATCGAGGAGCGCCTGAAGACTTACGCCCATCTCTTCGATGACAAGG
TCATGAAGCAGCTCAAGAGGAGGAGGTACACCGGCTGGGGGAGGCTGAGCAGGAAGCTCATCAACGGCATTCGGGACAAGCAGTCCGGGAAGACGATCCTCGA
CTTCCTGAAGAGCGATGGCTTCGCGAACCGCAATTTCATGCAGCTGATTCACGATGACAGCCTCACATTCAAGGAGGATATCCAGAAGGCTCAGGTGAGCGGC
CAGGGGGACTCGCTGCACGAGCATATCGCGAACCTCGCTGGCTCGCCAGCTATCAAGAAGGGGATTCTGCAGACCGTGAAGGTTGTGGACGAGCTGGTGAAGG
TCATGGGCAGGCACAAGCCTGAGAACATCGTCATTGAGATGGCCCGGGAGAATCAGACCACGCAGAAGGGCCAGAAGAACTCACGCGAGAGGATGAAGAGGAT
CGAGGAGGGCATTAAGGAGCTGGGGTCCCAGATCCTCAAGGAGCACCCGGTGGAGAACACGCAGCTGCAGAATGAGAAGCTCTACCTGTACTACCTCCAGAAT
GGCCGCGATATGTATGTGGACCAGGAGCTGGATATTAACAGGCTCAGCGATTACGACGTCGATCATATCGTTCCACAGTCATTCCTGAAGGATGACTCCATTG
ACAACAAGGTCCTCACCAGGTCGGACAAGAACCGGGGCAAGTCTGATAATGTTCCTTCAGAGGAGGTCGTTAAGAAGATGAAGAACTACTGGCGCCAGCTCCT
GAATGCCAAGCTGATCACGCAGCGGAAGTTCGATAACCTCACAAAGGCTGAGAGGGGCGGGCTCTCTGAGCTGGACAAGGCGGGCTTCATCAAGAGGCAGCTG
GTCGAGACACGGCAGATCACTAAGCACGTTGCGCAGATTCTCGACTCACGGATGAACACTAAGTACGATGAGAATGACAAGCTGATCCGCGAGGTGAAGGTCA
TCACCCTGAAGTCAAAGCTCGTCTCCGACTTCAGGAAGGATTTCCAGTTCTACAAGGTTCGGGAGATCAACAATTACCACCATGCCCATGACGCGTACCTGAA
CGCGGTGGTCGGCACAGCTCTGATCAAGAAGTACCCAAAGCTCGAGAGCGAGTTCGTGTACGGGGACTACAAGGTTTACGATGTGAGGAAGATGATCGCCAAG
TCGGAGCAGGAGATTGGCAAGGCTACCGCCAAGTACTTCTTCTACTCTAACATTATGAATTTCTTCAAGACAGAGATCACTCTGGCCAATGGCGAGATCCGGA
AGCGCCCCCTCATCGAGACGAACGGCGAGACGGGGGAGATCGTGTGGGACAAGGGCAGGGATTTCGCGACCGTCAGGAAGGTTCTCTCCATGCCACAAGTGAA
TATCGTCAAGAAGACAGAGGTCCAGACTGGCGGGTTCTCTAAGGAGTCAATTCTGCCTAAGCGGAACAGCGACAAGCTCATCGCCCGCAAGAAGGACTGGGAT
CCGAAGAAGTACGGCGGGTTCGACAGCCCCACTGTGGCCTACTCGGTCCTGGTTGTGGCGAAGGTTGAGAAGGGCAAGTCCAAGAAGCTCAAGAGCGTGAAGG
AGCTGCTGGGGATCACGATTATGGAGCGCTCCAGCTTCGAGAAGAACCCGATCGATTTCCTGGAGGCGAAGGGCTACAAGGAGGTGAAGAAGGACCTGATCAT
TAAGCTCCCCAAGTACTCACTCTTCGAGCTGGAGAACGGCAGGAAGCGGATGCTGGCTTCCGCTGGCGAGCTGCAGAAGGGGAACGAGCTGGCTCTGCCGTCC

AAGTATGTGAACTTCCTCTACCTGGCCTCCCACTACGAGAAGCTCAAGGGCAGCCCCGAGGACAACGAGCAGAAGCAGCTGTTCGTCGAGCAGCACAAGCATT
ACCTCGACGAGATCATTGAGCAGATTTCCGAGTTCTCCAAGCGCGTGATCCTGGCCGACGCGAATCTGGATAAGGTCCTCTCCGCGTACAACAAGCACCGCGA
CAAGCCAATCAGGGAGCAGGCTGAGAATATCATTCATCTCTTCACCCTGACGAACCTCGGCGCCCCTGCTGCTTTCAAGTACTTCGACACAACTATCGATCGC
AAGAGGTACACAAGCACTAAGGAGGTCCTGGACGCGACCCTCATCCACCAGTCGATTACCGGCCTCTACGAGACGCGCATCGACCTGTCTCAGCTCGGGGGCG
ACAAGCGGCCAGCGGCGACGAAGAAGGCGGGGCAGGCGAAGAAGAAGAAG**TGA**ATTATCAGAGTGGCGCAGCGGAAGCGTGGTGGGCCCATAACCCACAGGTC
CCAGGATCGAAACCTGGCTCTGATATCGAGAGAGCTTTCGTTCGTATCATCGGTTTCGACAACGTT…….

3. pMDC7_*Cas9-tRNA*$^{Met\ \Delta DT}$*(TLS2)*

GTTTACCCGCCAATATATCCTGTCAAACACTGATAGTTTAAACTGAAGGCGGGAAACGACAATCTGATCCAAGCTCAAGCTAAGCTTGCATGCCTGCAGGATA
TCGTGGATCCAAGCTTGCCACGTGCCGCCACGTGCCGCCACGTGCCGCCACGTGCCTCTAGAGGATCCATCTCCACTGACGTAAGGGATGACGCACAATCCCA
CTATCCTTCGCAAGACCCTTCCTCTATATAAGGAAGTTCATTTCATTTGGAGAGGACACGCTGGGATCCCCAATTCCGGGCGGAATGAAAGCGTTAACGGCCA
GGCAACAAGAGGTGTTTGATCTCATCCGTGA……………………………..TGCATGCCAGCTTGGGCTGCAGGTCGAGGCTAAAAAACTAATCGCATTATCATCCCCTCG
ACGTACTGTACATATAACCACTGGTTTTATATACAGCAGTACTGTACATATAACCACTGGTTTTATATACAGCAGTCGACGTACTGTACATATAACCACTGGT
TTTATATACAGCAGTACTGTACATATAACCACTGGTTTTATATACAGCAGTCGAGGTAAGATTAGATATGGATATGTATATGGATATGTATATGGTGGTAATG
CCATGTAATATGCTCGACTCTAGGATCTTCGCAAGACCCTTCCTCTATATAAGGAAGTTCATTTCATTTGGAGAGGACACGCTGAAGCTAGTCGACTCTAGCC
TCGAATGGATTACAAGGACCACGACGGGGATTACAAGGACCACGACATTGATTACAAGGATGATGATGACAAGATGGCTCCGAAGAAGAAGAGGAAGGTTGGC
ATCCACGGGGTGCCAGCTGCTGACAAGAAGTACTCGATCGGCCTCGATATTGGGACTAACTCTGTTGGCTGGGCCGTGATCACCGACGAGTACAAGGTGCCCT
CAAAGAAGTTCAAGGTCCTGGGCAACACCGATCGGCATTCCATCAAGAAGAATCTCATTGGCGCTCTCCTGTTCGACAGCGGCGAGACGGCTGAGGCTACGCG
GCTCAAGCGCACCGCCCGCAGGCGGTACACGCGCAGGAAGAATCGCATCTGCTACCTGCAGGAGATTTTCTCCAACGAGATGGCGAAGGTTGACGATTCTTTC
TTCCACAGGCTGGAGGAGTCATTCCTCGTGGAGGAGGATAAGAAGCACGAGCGGCATCCAATCTTCGGCAACATTGTCGACGAGGTTGCCTACCACGAGAAGT
ACCCTACGATCTACCATCTGCGGAAGAAGCTCGTGGACTCCACAGATAAGGCGGACCTCCGCCTGATCTACCTCGCTCTGGCCCACATGATTAAGTTCAGGGG
CCATTTCCTGATCGAGGGGGATCTCAACCCGGACAATAGCGATGTTGACAAGCTGTTCATCCAGCTCGTGCAGACGTACAACCAGCTCTTCGAGGAGAACCCC
ATTAATGCGTCAGGCGTCGACGCGAAGGCTATCCTGTCCGCTAGGCTCTCGAAGTCTCGGCGCCTCGAGAACCTGATCGCCCAGCTGCCGGGCGAGAAGAAGA
ACGGCCTGTTCGGGAATCTCATTGCGCTCAGCCTGGGGCTCACGCCCAACTTCAAGTCGAATTTCGATCTCGCTGAGGACGCCAAGCTGCAGCTCTCCAAGGA
CACATACGACGATGACCTGGATAACCTCCTGGCCCAGATCGGCGATCAGTACGCGGACCTGTTCCTCGCTGCCAAGAATCTGTCGGACGCCATCCTCCTGTCT
GATATTCTCAGGGTGAACACCGAGATTACGAAGGCTCCGCTCTCAGCCTCCATGATCAAGCGCTACGACGAGCACCATCAGGATCTGACCCTCCTGAAGGCGC
TGGTCAGGCAGCAGCTCCCCGAGAAGTACAAGGAGATCTTCTTCGATCAGTCGAAGAACGGCTACGCTGGGTACATTGACGGCGGGGCCTCTCAGGAGGAGTT
CTACAAGTTCATCAAGCCGATTCTGGAGAAGATGGACGGCACGGAGGAGCTGCTGGTGAAGCTCAATCGCGAGGACCTCCTGAGGAAGCAGCGGACATTCGAT
AACGGCAGCATCCCACACCAGATTCATCTCGGGGAGCTGCACGCTATCCTGAGGAGGCAGGAGGACTTCTACCCTTTCCTCAAGGATAACCGCGAGAAGATCG
AGAAGATTCTGACTTTCAGGATCCCGTACTACGTCGGCCCACTCGCTAGGGGCAACTCCCGCTTCGCTTGGATGACCCGCAAGTCAGAGGAGACGATCACGCC
GTGGAACTTCGAGGAGGTGGTCGACAAGGGCGCTAGCGCTCAGTCGTTCATCGAGAGGATGACGAATTTCGACAAGAACCTGCCAAATGAGAAGGTGCTCCCT
AAGCACTCGCTCCTGTACGAGTACTTCACAGTCTACAACGAGCTGACTAAGGTGAAGTATGTGACCGAGGGCATGAGGAAGCCGGCTTTCCTGTCTGGGGAGC
AGAAGAAGGCCATCGTGGACCTCCTGTTCAAGACCAACCGGAAGGTCACGGTTAAGCAGCTCAAGGAGGACTACTTCAAGAAGATTGAGTGCTTCGATTCGGT
CGAGATCTCTGGCGTTGAGGACCGCTTCAACGCCTCCCTGGGGACCTACCACGATCTCCTGAAGATCATTAAGGATAAGGACTTCCTGGACAACGAGGAGAAT
GAGGATATCCTCGAGGACATTGTGCTGACACTCACTCTGTTCGAGGACCGGGAGATGATCGAGGAGCGCCTGAAGACTTACGCCCATCTCTTCGATGACAAGG
TCATGAAGCAGCTCAAGAGGAGGAGGTACACCGGCTGGGGGAGGCTGAGCAGGAAGCTCATCAACGGCATTCGGGACAAGCAGTCCGGGAAGACGATCCTCGA
CTTCCTGAAGAGCGATGGCTTCGCGAACCGCAATTTCATGCAGCTGATTCACGATGACAGCCTCACATTCAAGGAGGATATCCAGAAGGCTCAGGTGAGCGGC
CAGGGGGACTCGCTGCACGAGCATATCGCGAACCTCGCTGGCTCGCCAGCTATCAAGAAGGGGATTCTGCAGACCGTGAAGGTTGTGGACGAGCTGGTGAAGG
TCATGGGCAGGCACAAGCCTGAGAACATCGTCATTGAGATGGCCCGGGAGAATCAGACCACGCAGAAGGGCCAGAAGAACTCACGCGAGAGGATGAAGAGGAT
CGAGGAGGGCATTAAGGAGCTGGGGTCCCAGATCCTCAAGGAGCACCCGGTGGAGAACACGCAGCTGCAGAATGAGAAGCTCTACCTGTACTACCTCCAGAAT
GGCCGCGATATGTATGTGGACCAGGAGCTGGATATTAACAGGCTCAGCGATTACGACGTCGATCATATCGTTCCACAGTCATTCCTGAAGGATGACTCCATTG
ACAACAAGGTCCTCACCAGGTCGGACAAGAACCGGGGCAAGTCTGATAATGTTCCTTCAGAGGAGGTCGTTAAGAAGATGAAGAACTACTGGCGCCAGCTCCT
GAATGCCAAGCTGATCACGCAGCGGAAGTTCGATAACCTCACAAAGGCTGAGAGGGGCGGGCTCTCTGAGCTGGACAAGGCGGGCTTCATCAAGAGGCAGCTG
GTCGAGACACGGCAGATCACTAAGCACGTTGCGCAGATTCTCGACTCACGGATGAACACTAAGTACGATGAGAATGACAAGCTGATCCGCGAGGTGAAGGTCA
TCACCCTGAAGTCAAAGCTCGTCTCCGACTTCAGGAAGGATTTCCAGTTCTACAAGGTTCGGGAGATCAACAATTACCACCATGCCCATGACGCGTACCTGAA
CGCGGTGGTCGGCACAGCTCTGATCAAGAAGTACCCAAAGCTCGAGAGCGAGTTCGTGTACGGGGACTACAAGGTTTACGATGTGAGGAAGATGATCGCCAAG
TCGGAGCAGGAGATTGGCAAGGCTACCGCCAAGTACTTCTTCTACTCTAACATTATGAATTTCTTCAAGACAGAGATCACTCTGGCCAATGGCGAGATCCGGA
AGCGCCCCCTCATCGAGACGAACGGCGAGACGGGGGAGATCGTGTGGGACAAGGGCAGGGATTTCGCGACCGTCAGGAAGGTTCTCTCCATGCCACAAGTGAA
TATCGTCAAGAAGACAGAGGTCCAGACTGGCGGGTTCTCTAAGGAGTCAATTCTGCCTAAGCGGAACAGCGACAAGCTCATCGCCCGCAAGAAGGACTGGGAT
CCGAAGAAGTACGGCGGGTTCGACAGCCCCACTGTGGCCTACTCGGTCCTGGTTGTGGCGAAGGTTGAGAAGGGCAAGTCCAAGAAGCTCAAGAGCGTGAAGG
AGCTGCTGGGGATCACGATTATGGAGCGCTCCAGCTTCGAGAAGAACCCGATCGATTTCCTGGAGGCGAAGGGCTACAAGGAGGTGAAGAAGGACCTGATCAT
TAAGCTCCCCAAGTACTCACTCTTCGAGCTGGAGAACGGCAGGAAGCGGATGCTGGCTTCCGCTGGCGAGCTGCAGAAGGGGAACGAGCTGGCTCTGCCGTCC
AAGTATGTGAACTTCCTCTACCTGGCCTCCCACTACGAGAAGCTCAAGGGCAGCCCCGAGGACAACGAGCAGAAGCAGCTGTTCGTCGAGCAGCACAAGCATT
ACCTCGACGAGATCATTGAGCAGATTTCCGAGTTCTCCAAGCGCGTGATCCTGGCCGACGCGAATCTGGATAAGGTCCTCTCCGCGTACAACAAGCACCGCGA
CAAGCCAATCAGGGAGCAGGCTGAGAATATCATTCATCTCTTCACCCTGACGAACCTCGGCGCCCCTGCTGCTTTCAAGTACTTCGACACAACTATCGATCGC
AAGAGGTACACAAGCACTAAGGAGGTCCTGGACGCGACCCTCATCCACCAGTCGATTACCGGCCTCTACGAGACGCGCATCGACCTGTCTCAGCTCGGGGGCG
ACAAGCGGCCAGCGGCGACGAAGAAGGCGGGGCAGGCGAAGAAGAAGAAGTGAATTATCAGAGTGGTGGTGGGCCCATAACCCACAGGTCCGCTCTGATATCG
AGAGAGCTTTCGTTCGTATCATCGGTTTCGACAACGTT……………

b, Sequences of the *gNIA1*, pMDC100_*gNIA1-tRNA^{Met} (TLS1)* and *gNIA1-tRNA$^{Met\ \Delta DT}$ (TLS2)* constructs in the binary pMDC100 vector used in the study.

Light Grey colour background indicates *pU6-26* promoter; Dark Grey colour background indicates *pU6-29* promoter. Red colour background indicates two *NIA1* target sequences. Light blue colour background indicates *gRNA* Scaffold sequence; Green colour background indicates *tU6-26* terminator sequence. Yellow colour background indicates *Kanamycin* resistance gene sequence. Pink colour background indicates *tRNA^{Met}* (TLS1) or *tRNA$^{Met\ \Delta DT}$* (TLS2) sequences.

1. pMDC100_*gNIA1*

cacatacaaatggacgaacggataaaccttttcacgcccttttaaatatccgttattctaataaacgctcttttctcttaggtttacccgccaatatatcctg
tcaaacactgatagtttaaactgaaggcgggaaacgacaatctgatccaagctcaagctgctctagcattcgccattcaggctgcgcaactgttgggaagggc
gatcggtgcgggcctcttcgctattacgccagctggcgaaagggggatgtgctgcaaggcgattaagttgggtaacgccagggttttcccagtcacgacgttg
taaaacgacggccagtgccaagcttgcatgcctgcaggtcgactctagaggatccccgggtaccgggcccccctcgaggcgcgccaagcCAAGTTTGTACAA
AAAAGCAGGCTCCACCATGGGAACCAATTCAGTCGACTGGATCCcgacttgccttccgcacaatacatcatttcttcttagctttttttcttcttcttcgttc
atacagtttttttttgtttatcagcttacattttcttgaaccgtagctttcgttttcttctttttaactttccattcggagtttttgtatcttgtttcatagt
ttgtcccaggattagaatgattaggcatcgaaccttcaagaatttgattgaataaaacatcttcattcttaagatatgaagataatcttcaaaaggcccctgg
gaatctgaaagaagagaagcaggcccatttatatgggaaagaacaatagtatttcttatataggcccatttaagttgaaaacaatcttcaaaagtcccacatc
gcttagataagaaaacgaagctgagtttatatacagctagagtcgaagtagtgATTGACAACACTGCTGACTCTGCAGTTTtagagctagaaatagcaagtta
aaataaggctagtccgttatcaacttgaaaaagtggcaccgagtcggtgctttttttgcaaaattttccagatcgatttcttcttcctctgttcttcggcgt
tcaatttctggggttttctcttcgttttctgtaactgaaacctaaaatttgacctaaaaaaaatctcaaataatatgattcagtggttttgtacttttcagtt
agttgagttttgcagttccgatgagataaaccaatattaatccaaactactgcagcctgacagacaaatgaggatgcaaacaattttaaagtttatctaacgc
tagctgttttgtttcttctctctggtgcaccaacgacggcgttttctcaatcataaagaggcttgttttacttaaggccaataatgttgatggatcgaaagaa
gagggcttttaataaacgagcccgtttaagctgtaaacgatgtcaaaaacatcccacatcgttcagttgaaaatagaagctctgtttatatattggtagagtc
gactaagagATTGATGGGTTACAACGTGAAGGGTTTtagagctagaaatagcaagttaaaataaggctagtccgttatcaacttgaaaaagtggcaccgagtc
ggtgctttttttgcaaaattttccagatcgatttcttcttcctctgttcttcggcgttcaatttctggggttttctcttcgttttctgtaactgaaacctaa
aatttgacctaaaaaaaatctcaaataatatgattcagtggttttgtacttttcagttagttgagttttgcagttccgatgagataaaccaataCTCGAGATA
TCTAGACCCAGCTTTCTTGTACAAAGTGGtcgataattccttaattaactagttctagagcggccgccaccgcggtggagctcgaattcgtaatcatggtcat
agctgtttcctgtgtgaaattgttatccgctcacaattccacacaacatacgagccggaagcataaagtgtaaagcctggggtgcctaatgagtgagctaact
cacattaattgcgttgcgctcactgcccgctttccagtcgggaaacctgtcgtgccagctgcattaatgaatcggccaacgcgcggggagaggcggtttgcgt
attggctagagcagcttgccaacatggtggagcacgacactctcgtctactccaagaatatcaaagatacagtctcagaagaccaaagggctattgagacttt
tcaacaaagggtaatatcgggaaacctcctcggattccattgcccagctatctgtcacttcatcaaaaggacagtagaaaaggaaggtggcacctacaaatgc
catcattgcgataaaggaaaggctatcgttcaagatgcctctgccgacagtggtcccaaagatggacccccacccacgaggagcatcgtggaaaaagaagacg
ttccaaccacgtcttcaaagcaagtggattgatgtgataacatggtggagcacgacactctcgtctactccaagaatatcaaagatacagtctcagaagacca
aagggctattgagacttttcaacaaagggtaatatcgggaaacctcctcggattccattgcccagctatctgtcacttcatcaaaaggacagtagaaaaggaa
ggtggcacctacaaatgccatcattgcgataaaggaaaggctatcgttcaagatgcctctgccgacagtggtcccaaagatggacccccacccacgaggagca
tcgtggaaaaagaagacgttccaaccacgtcttcaaagcaagtggattgatgtgatatctccactgacgtaagggatgacgcacaatcccactatccttcgca
agacccttcctctatataaggaagttcatttcatttggagaggacacgctgaaatcaccagtctctctctacaaatctatctctctcgagctttcgcagatctg
tcgatcgaccatggggattgaacaagatggattgcacgcaggttctccggccgcttgggtggagaggctattcggctatgactgggcacaacagacaatcggc
tgctctgatgccgccgtgttccggctgtcagcgcaggggcgcccggttctttttgtcaagaccgacctgtccggtgccctgaatgaactccaggacgaggcag
cgcggctatcgtggctggccacgacgggcgttccttgcgcagctgtgctcgacgttgtcactgaagcgggaagggactggctgctattgggcgaagtgccggg
gcaggatctcctgtcatctcaccttgctcctgccgagaaagtatccatcatggctgatgcaatgcggcggctgcatacgcttgatccggctacctgcccattc
gaccaccaagcgaaacatcgcatcgagcgagcacgtactcggatggaagccggtcttgtcgatcaggatgatctggacgaagagcatcaggggctcgcgccag
ccgaactgttcgccaggctcaaggcgcgcatgcccgacggcgaggatctcgtcgtgacacatggcgatgcctgcttgccgaatatcatggtggaaaatggccg
cttttctggattcatcgactgtggccggctgggtgtggcggaccgctatcaggacatagcgttggctacccgtgatattgctgaagagcttggcggcgaatgg
gctgaccgcttcctcgtgctttacggtatcgccgctcccgattcgcagcgcatcgccttctatcgccttcttgacgagttcttctgagcgggactctggggtt
cggatcgatcctctagctagagtcgatcgacaagctcgagtttctccataataatgtgtgagtagttcccagataagggaattagggttcctatagggtttcg
ctcatgtgttgagcatataagaaacccttagtatgtatttgtatttgtaaaatacttctatcaataaaatttctaattcctaaaaccaaaatccagtactaaa
atccagatcccccgaattaattcggcgttaattcagtacattaaaaacgtccgcaatgtgttattaagttgtctaagcgtcaatttgtttacaccacaatata
tcctgccaccagccagccaacagctccccgaccggcagctcggcacaaaatcaccactcgatacaggcagcccatcagtccgggacggcgtcagcgggagagc
cgttgtaaggcggcagactttgctcatgttaccgatgctattcggaagaacggcaactaagctgccgggtttgaaacacggatgatctcgcggagggtagcat
gttgattgtaacgatgacagagcgttgctgcctgtgatcaccgcggtttcaaaatcggctccgtcgatactatgttatacgccaactttgaaaacaactttga
aaaagctgttttctggtatttaaggttttagaatgcaaggaacagtgaattggagttcgtcttgttataattagcttcttggggtatctttaaatactgtaga
aaagaggaaggaaataataaatggctaaaatgagaatatcaccggaattgaaaaaactgatcgaaaaataccgctgcgtaaaagatacggaaggaatgtctcc
tgctaaggtatataagctggtgggagaaaatgaaaacctatatttaaaaatgacggacagccggtataaagggaccacctatgatgtggaacgggaaaaggac
atgatgctatggctggaaggaaagctgcctgttccaaaggtcctgcactttgaacggcatgatggctggagcaatctgctcatgagtgaggccgatggcgtcc
tttgctcggaagagtatgaagatgaacaaagccctgaaaagattatcgagctgtatgcggagtgcatcaggctctttcactccatcgacatatcggattgtcc
ctatacgaatagcttagacagccgcttagccgaattggattacttactgaataacgatctggccgatgtggattgcgaaaactgggaagaagacactccattt
aaagatccgcgcgagctgtatgatttttaaagacggaaaagcccgaagaggaacttgtcttttcccacggcgacctgggagacagcaacatctttgtgaaag
atggcaaagtaagtggctttattgatcttgggagaagcggcagggcggacaagtggtatgacattgccttctgcgtccggtcgatcagggaggatatcgggga
agaacagtatgtcgagctatttttgacttactggggatcaagcctgattgggagaaaataaaatattatattttactggatgaattgttttagtacctagaa
tgcatgaccaaaatcccttaacgtgagttttcgttccactgagcgtcagaccccgtagaaaagatcaaaggatcttcttgagatcctttttttctgcgcgtaa
tctgctgcttgcaaacaaaaaaaccaccgctaccagcggtggtttgtttgccggatcaagagctaccaactctttttccgaaggtaactggcttcagcagagc
gcagataccaaatactgtccttctagtgtagccgtagttaggccaccacttcaagaactctgtagcaccgcctacatacctcgctctgctaatcctgttacca
gtggctgctgccagtggcgataagtcgtgtcttaccgggttggactcaagacgatagttaccggataaggcgcagcggtcgggctgaacggggggttcgtgca
cacagcccagcttggagcgaacgacctacaccgaactgagatacctacagcgtgagctatgagaaagcgccacgcttcccgaagggagaaaggcggacaggta
tccggtaagcggcagggtcggaacaggagagcgcacgagggagcttccagggggaaacgcctggtatctttatagtcctgtcgggtttcgccacctctgactt
gagcgtcgatttttgtgatgctcgtcaggggggcggagcctatggaaaaacgccagcaacgcggcctttttacggttcctggccttttgctggccttttgctc
acatgttctttcctgcgttatcccctgattctgtggataaccgtattaccgcctttgagtgagctgataccgctcgccgcagccgaacgaccgagcgcagcga

gtcagtgagcgaggaagcggaagagcgcctgatgcggtattttctccttacgcatctgtgcggtatttcacaccgcatatggtgcactctcagtacaatctgc
tctgatgccgcatagttaagccagtatacactccgctatcgctacgtgactgggtcatggctgcgccccgacacccgccaacacccgctgacgcgccctgacg
ggcttgtctgctcccggcatccgcttacagacaagctgtgaccgtctccgggagctgcatgtgtcagaggttttcaccgtcatcaccgaaacgcgcgaggcag
ggtgccttgatgtgggcgccggcggtcgagtggcgacggcgcggcttgtccgcgccctggtagattgcctggccgtaggccagccatttttgagcggccagcg
gccgcgataggccgacgcgaagcggcggggcgtagggagcgcagcgaccgaagggtaggcgctttttgcagctcttcggctgtgcgctggccagacagttatg
cacaggccaggcgggttttaagagttttaataagttttaaagagttttaggcggaaaaatcgcctttttctcttttatatcagtcacttacatgtgtgaccg
gttcccaatgtacggctttgggttcccaatgtacgggttccggttcccaatgtacggctttgggttcccaatgtacgtgctatccacaggaaagagaccttttt
cgaccttttcccctgctagggcaatttgccctagcatctgctccgtacattaggaaccggcggatgcttcgccctcgatcaggttgcggtagcgcatgacta
ggatcgggccagcctgccccgcctcctccttcaaatcgtactccggcaggtcatttgacccgatcagcttgcgcacggtgaaacagaacttcttgaactctcc
ggcgctgccactgcgttcgtagatcgtcttgaacaaccatctggcttctgccttgcctgcggcgcggcgtgccaggcggtagagaaaacggccgatgccggga
tcgatcaaaaagtaatcggggtgaaccgtcagcacgtccgggttcttgccttctgtgatctcgcggtacatccaatcagctagctcgatctcgatgtactccg
gccgcccggtttcgctctttacgatcttgtagcggctaatcaaggcttcaccctcggataccgtcaccaggcggccgttcttggccttcttcgtacgctgcat
ggcaacgtgcgtggtgtttaaccgaatgcaggtttctaccaggtcgtctttctgctttccgccatcggctcgccggcagaacttgagtacgtccgcaacgtgt
ggacggaacacgcggccgggcttgtctcccttcccttcccggtatcggttcatggattcggttagatgggaaaccgccatcagtaccaggtcgtaatcccaca
cactggccatgccggccggccctgcggaaacctctacgtgcccgtctggaagctcgtagcggatcacctcgccagctcgtcggtcacgcttcgacagacggaa
aacggccacgtccatgatgctgcgactatcgcgggtgcccacgtcatagagcatcggaacgaaaaaatctggttgctcgtcgcccttgggcggcttcctaatc
gacggcgcaccggctgccggcggttgccgggattctttgcggattcgatcagcggccgcttgccacgattcaccggggcgtgcttctgcctcgatgcgttgcc
gctgggcggcctgcgcggccttcaacttctccaccaggtcatcacccagcgccgcgccgatttgtaccgggccggatggtttgcgaccgtcacgccgattcct
cgggcttgggggttccagtgccattgcagggccggcagacaacccagccgcttacgcctggccaaccgcccgttcctccacacatggggcattccacggcgtc
ggtgcctggttgttcttgattttccatgccgcctcctttagccgctaaaattcatctactcatttattcatttgctcatttactctggtagctgcgcgatgta
ttcagatagcagctcggtaatggtcttgccttggcgtaccgcgtacatcttcagcttggtgtgatcctccgccggcaactgaaagttgacccgcttcatggct
ggcgtgtctgccaggctggccaacgttgcagccttgctgctgcgtgcgctcggacggccggcacttagcgtgtttgtgcttttgctcatttctctttacctc
attaactcaaatgagttttgatttaatttcagcggccagcgcctggacctcgcgggcagcgtcgccctcgggttctgattcaagaacggttgtgccggcggcg
gcagtgcctgggtagctcacgcgctgcgtgatacgggactcaagaatgggcagctcgtacccggccagcgcctcggcaacctcaccgccgatgcgcgtgcctt
tgatcgcccgcgacacgacaaaggccgcttgtagccttccatccgtgacctcaatgcgctgcttaaccagctccaccaggtcggcggtggcccatatgtcgta
agggcttggctgcaccggaatcagcacgaagtcggctgccttgatcgcggacacagccaagtccgccgcctggggcgctccgtcgatcactacgaagtcgcgc
cggccgatggccttcacgtcgcggtcaatcgtcgggcggtcgatgccgacaacggttagcggttgatcttcccgcacggccgcccaatcgcgggcactgccct
ggggatcggaatcgactaacagaacatcggccccggcgagttgcagggcgcgggctagatgggttgcgatggtcgtcttgcctgacccgcctttctggttaag
tacagcgataaccttcatgcgttccccttgcgtatttgtttatttactcatcgcatcatatacgcagcgaccgcatgacgcaagctgttttactcaaatacac
atcaccttttttagacggcggcgctcggtttcttcagcggccaagctggccggccaggccgccagcttggcatcagacaaaccggccaggatttcatgcagccg
cacggttgagacgtgcgcgggcggctcgaacacgtacccggccgcgatcatctccgcctcgatctcttcggtaatgaaaaacggttcgtcctggccgtcctgg
tgcggtttcatgcttgttcctcttggcgttcattctcggcggccgccagggcgtcggcctcggtcaatgcgtcctcacggaaggcaccgcgccgcctggcctc
ggtgggcgtcacttcctcgctgcgctcaagtgcgcggtacagggtcgagcgatgcacgccaagcagtgcagccgcctctttcacggtgcggccttcctggtcg
atcagctcgcgggcgtgcgcgatctgtgccggggtgagggtagggcgggggccaaacttcacgcctcgggccttggcggcctcgcgcccgctccgggtgcggt
cgatgattagggaacgctcgaactcggcaatgccggcgaacacggtcaacaccatgcggccggccggcgtggtggtgtcggcccacggctctgccaggctacg
caggcccgcgccggcctcctggatgcgctcggcaatgtccagtaggtcgcgggtgctgcgggccaggcggtctagcctggtcactgtcacaacgtcgccaggg
cgtaggtggtcaagcatcctggccagctccgggcggtcgcgcctggtgccggtgatcttctcggaaaacagcttggtgcagccggccgcgtgcagttcggccc
gttggttggtcaagtcctggtcgtcggtgctgacgcgggcatagcccagcaggccagcggcggcgctcttgttcatggcgtaatgtctccggttctagtcgca
agtattctactttatgcgactaaaacacgcgacaagaaaacgccaggaaaagggcagggcggcagcctgtcgcgtaacttaggacttgtgcgacatgtcgttt
tcagaagacggctgcactgaacgtcagaagccgactgcactatagcagcggaggggttggatcaaagtactttgatcccgaggggaaccctgtggttggcatg

2. pMDC100_*gNIA1-tRNA*^{Met} *(TLS1)*

cacatacaaatggacgaacggataaaccttttcacgcccttttaaatatccgttattctaataaacgctcttttctcttaggtttacccgccaatatatcctg
tcaaacactgatagtttaaactgaaggcgggaaacgacaatctgatccaagctcaagctgctctagcattcgccattcaggctgcgcaactgttgggaagggc
gatcggtgcgggcctcttcgctattacgccagctggcgaaagggggatgtgctgcaaggcgattaagttgggtaacgccagggttttcccagtcacgacgttg
taaaacgacggccagtgccaagcttgcatgcctgcaggtcgactctagaggatccccgggtaccgggccccccctcgaggcgcgccaagcCAAGTTTGTACAA
AAAAGCAGGCTCCACCATGGGAACCAATTCAGTCGACTGGATCCcgacttgccttccgcacaatacatcatttcttcttagctttttttcttcttcttcgttc
atacagtttttttttgtttatcagcttacatttcttgaaccgtagctttcgttttcttctttttaactttccattcggagttttgtatcttgtttcatagt
ttgtcccaggattagaatgattaggcatcgaaccttcaagaatttgattgaataaaacatcttcattcttaagatatgaagataatcttcaaaaggcccctgg
gaatctgaaagaagagaagcaggcccatttatatgggaaagaacaatagtatttcttatataggcccatttaagttgaaaacaatcttcaaaagtcccacatc
gcttagataagaaaacgaagctgagtttatatacagctagagtcgaagtagtgATTGACAACACTGCTGACTCTGCAGTTTtagagctagaaatagcaagtta
aaataaggctagtccgttatcaacttgaaaaagtggcaccgagtcggtgcATTATCAGAGTGGCGCAGCGGAAGCGTGGTGGGCCCATAACCCACAGGTCCCA
GGATCGAAACCTGGCTCTGATA**AAAAAAAA**ttttttttgcaaaattttccagatcgatttcttcttcctctgttcttcggcgttcaatttctggggttttctc
ttcgttttctgtaactgaaacctaaaatttgacctaaaaaaaatctcaaataatatgattcagtggttttgtacttttcagttagttgagttttgcagttccg
atgagataaaccaatattaatccaaactactgcagcctgacagacaaatgaggatgcaaacaatttaaagtttatctaacgctagctgttttgtttcttctc
tctggtgcaccaacgacggcgttttctcaatcataaagaggcttgttttacttaaggccaataatgttgatggatcgaaagaagaggcttttaataaacgag
cccgtttaagctgtaaacgatgtcaaaaacatcccacatcgttcagttgaaaatagaagctctgtttatatattggtagagtcgactaagagATTGATGGGTT
ACAACGTGAAGGGTTTtagagctagaaatagcaagttaaaataaggctagtccgttatcaacttgaaaaagtggcaccgagtcggtgcATTATCAGAGTGGCG
CAGCGGAAGCGTGGTGGGCCCATAACCCACAGGTCCCAGGATCGAAACCTGGCTCTGATA**AAAAAAAA**ttttttttgcaaaattttccagatcgatttcttct
tcctctgttcttcggcgttcaatttctggggttttctcttcgttttctgtaactgaaacctaaaatttgacctaaaaaaaatctcaaataatatgattcagtg
gttttgtacttttcagttagttgagttttgcagttccgatgagataaaccaataCTCGAGATATCTAGACCCAGCTTTCTTGTACAAAGTGGtcgataattcc
ttaattaactagttctagagcggccgccaccgcggtggagctcgaattcgtaatcatggtcatagctgtttcctgtgtgaaattgttatccgctcacaattcc
acacaacatacgagccggaagcataaagtgtaaagcctggggtgcctaatgagtgagctaactcacattaattgcgttgcgctcactgcccgctttccagtcg
ggaaacctgtcgtgccagctgcattaatgaatcggccaacgcgcggggagaggcggtttgcgtattggctagagcagcttgccaacatggtggagcacgacac
tctcgtctactccaagaatatcaaagatacagtctcagaagaccaaagggctattgagacttttcaacaaagggtaatatcgggaaacctcctcggattccat
tgcccagctatctgtcacttcatcaaaaggacagtagaaaaggaaggtggcacctacaaatgccatcattgcgataaaggaaaggctatcgttcaagatgcct
ctgccgacagtggtcccaaagatggacccccacccacgaggagcatcgtggaaaaagaagacgttccaaccacgtcttcaaagcaagtggattgatgtgataa
catggtggagcacgacactctcgtctactccaagaatatcaaagatacagtctcagaagaccaaagggctattgagacttttcaacaaagggtaatatcggga
aacctcctcggattccattgcccagctatctgtcacttcatcaaaaggacagtagaaaaggaaggtggcacctacaaatgccatcattgcgataaaggaaagg
ctatcgttcaagatgcctctgccgacagtggtcccaaagatggacccccacccacgaggagcatcgtggaaaaagaagacgttccaaccacgtcttcaaagca
agtggattgatgtgatatctccactgacgtaagggatgacgcacaatcccactatccttcgcaagaccttcctctatataaggaagttcatttcatttggaga
ggacacgctgaaatcaccagtctctctctacaaatctatctctctcgagctttcgcagatctgtcgatcgaccatggggattgaacaagatggattgcacgca
ggttctccggccgcttgggtggagaggctattcggctatgactgggcacaacagacaatcggctgctctgatgccgccgtgttccggctgtcagcgcaggggc
gcccggttctttttgtcaagaccgacctgtccggtgccctgaatgaactccaggacgaggcagcgcggctatcgtggctggccacgacgggcgttccttgcgc
agctgtgctcgacgttgtcactgaagcgggaagggactggctgctattgggcgaagtgccggggcaggatctcctgtcatctcaccttgctcctgccgagaaa

gtatccatcatggctgatgcaatgcggcggctgcatacgcttgatccggctacctgcccattcgaccaccaagcgaaacatcgcatcgagcgagcacgtactc
ggatggaagccggtcttgtcgatcaggatgatctggacgaagagcatcaggggctcgcgccagccgaactgttcgccaggctcaaggcgcgcatgcccgacgg
cgaggatctcgtcgtgacacatggcgatgcctgcttgccgaatatcatggtggaaaatggccgcttttctggattcatcgactgtggccggctgggtgtggcg
gaccgctatcaggacatagcgttggctacccgtgatattgctgaagagcttggcggcgaatgggctgaccgcttcctcgtgctttacggtatcgccgctcccg
attcgcagcgcatcgccttctatcgccttcttgacgagttcttctgagcgggact……………

3. pMDC100_*gNIA1-tRNA*$^{Met\ \Delta DT}$*(TLS2)*

cacatacaaatggacgaacggataaaccttttcacgcccttttaaatatccgttattctaataaacgctcttttctcttaggtttacccgccaatatatcctg
tcaaacactgatagtttaaactgaaggcgggaaacgacaatctgatccaagctcaagctgctctagcattcgccattcaggctgcgcaactgttgggaagggc
gatcggtgcgggcctcttcgctattacgccagctggcgaaagggggatgtgctgcaaggcgattaagttgggtaacgccagggttttcccagtcacgacgttg
taaaacgacggccagtgccaagcttgcatgcctgcaggtcgactctagaggatccccgggtaccgggccccccctcgaggcgcgccaagcCAAGTTTGTACAA
AAAAGCAGGCTCCACCATGGGAACCAATTCAGTCGACTGGATCCcgacttgccttccgcacaatacatcatttcttcttagctttttttcttcttcttcgttc
atacagtttttttttgtttatcagcttacattttcttgaaccgtagctttcgttttcttctttttaactttccattcggagtttttgtatcttgtttcatagt
ttgtcccaggattagaatgattaggcatcgaaccttcaagaatttgattgaataaaacatcttcattcttaagatatgaagataatcttcaaaaggcccctgg
gaatctgaaagaagagaagcaggcccatttatatgggaaagaacaatagtatttcttatataggcccatttaagttgaaaacaatcttcaaaagtcccacatc
gcttagataagaaaacgaagctgagtttatatacagctagagtcgaagtagtgATTGACAACACTGCTGACTCTGCAGTTTtagagctagaaatagcaagtta
aaataaggctagtccgttatcaacttgaaaaagtggcaccgagtcggtgcATTATCAGAGTGGTGGTGGGCCCATAACCCACAGGTCCGCTCTGATA**AAAAAA**
AAtttttttgcaaaatttccagatcgatttcttcttcctctgttcttcggcgttcaatttctggggttttctcttcgttttctgtaactgaaacctaaaat
ttgacctaaaaaaaatctcaaataatatgattcagtggttttgtacttttcagttagttgagttttgcagttccgatgagataaaccaatattaatccaaact
actgcagcctgacagacaaatgaggatgcaaacaattttaaagtttatctaacgctagctgttttgtttcttctctctggtgcaccaacgacggcgttttctc
aatcataaagaggcttgttttacttaaggccaataatgttgatggatcgaaagaagagggcttttaataaacgagcccgtttaagctgtaaacgatgtcaaaa
acatcccacatcgttcagttgaaaatagaagctctgtttatatattggtagagtcgactaagagATTGATGGGTTACAACGTGAAGGGTTTtagagctagaaa
tagcaagttaaaataaggctagtccgttatcaacttgaaaaagtggcaccgagtcggtgcATTATCAGAGTGGTGGTGGGCCCATAACCCACAGGTCCGCTCT
GATA**AAAAAAAA**tttttttgcaaaatttccagatcgatttcttcttcctctgttcttcggcgttcaatttctggggttttctcttcgttttctgtaactga
aacctaaaatttgacctaaaaaaaatctcaaataatatgattcagtggttttgtacttttcagttagttgagttttgcagttccgatgagataaaccaataCT
CGAGATATCTAGACCCAGCTTTCTTGTACAAAGTGGtcgataattccttaattaactagttctagagcggccgccaccgcggtggagctcgaattcgtaatca
tggtcatagctgtttcctgtgtgaaattgttatccgctcacaattccacacaacatacgagccggaagcataaagtgtaaagcctggggtgcctaatgagtga
gctaactcacattaattgcgttgcgctcactgcccgctttccagtcgggaaacctgtcgtgccagctgcattaatgaatcggccaacgcgcggggagaggcgg
tttgcgtattggctagagcagcttgccaacatggtggagcacgacactctcgtctactccaagaatatcaaagatacagtctcagaagaccaaagggctattg
agacttttcaacaaagggtaatatcgggaaacctcctcggattccattgcccagctatctgtcacttcatcaaaaggacagtagaaaaggaaggtggcaccta
caaatgccatcattgcgataaaggaaaggctatcgttcaagatgcctctgccgacagtggtcccaaagatggacccccacccacgaggagcatcgtggaaaaa
gaagacgttccaaccacgtcttcaaagcaagtggattgatgtgataacatggtggagcacgacactctcgtctactccaagaatatcaaagatacagtctcag
aagaccaaagggctattgagacttttcaacaaagggtaatatcgggaaacctcctcggattccattgcccagctatctgtcacttcatcaaaaggacagtaga
aaaggaaggtggcacctacaaatgccatcattgcgataaaggaaaggctatcgttcaagatgcctctgccgacagtggtcccaaagatggacccccacccacg
aggagcatcgtggaaaaagaagacgttccaaccacgtcttcaaagcaagtggattgatgtgatatctccactgacgtaagggatgacgcacaatcccactatc
cttcgcaagacccttcctctatataaggaagttcatttcatttggagaggacacgctgaaatcaccagtctctctctacaaatctatctctctcgagctttcgc
agatctgtcgatcgaccatggggattgaacaagatggattgcacgcaggttctccggccgcttgggtggagaggctattcggctatgactgggcacaacagac
aatcggctgctctgatgccgccgtgttccggctgtcagcgcaggggcgcccggttctttttgtcaagaccgacctgtccggtgccctgaatgaactccaggac
gaggcagcgcggctatcgtggctggccacgacgggcgttccttgcgcagctgtgctcgacgttgtcactgaagcgggaagggactggctgctattgggcgaag
tgccggggcaggatctcctgtcatctcaccttgctcctgccgagaaagtatccatcatggctgatgcaatgcggcggctgcatacgcttgatccggctacctg
cccattcgaccaccaagcgaaacatcgcatcgagcgagcacgtactcggatggaagccggtcttgtcgatcaggatgatctggacgaagagcatcaggggctc
gcgccagccgaactgttcgccaggctcaaggcgcgcatgcccgacggcgaggatctcgtcgtgacacatggcgatgcctgcttgccgaatatcatggtggaaa
atggccgcttttctggattcatcgactgtggccggctgggtgtggcggaccgctatcaggacatagcgttggctacccgtgatattgctgaagagcttggcgg
cgaatgggctgaccgcttcctcgtgctttacggtatcgccgctcccgattcgcagcgcatcgccttctatcgccttcttgacgagttcttctgagcgggact…
…………

c, Sequences of the *gVenus*, pMDC100_ *gVenus-tRNA*^{Met} *(TLS1)* and *gVenus-tRNA*$^{Met\ \Delta DT}$ *(TLS2)* constructs in the binary pMDC100 vector used in the study.

Light Grey colour background indicates *pU6-26* promoter; Dark Grey colour background indicates *pU6-29* promoter. Red colour background indicates two *NIA1* target sequences. Light blue colour background indicates *gRNA* Scaffold sequence; Green colour background indicates *tU6-26* terminator sequence. Yellow colour background indicates *Kanamycin* resistance gene sequence. Pink colour background indicates *tRNA*^{Met} (TLS1) or *tRNA*$^{Met\ \Delta DT}$ (TLS2) sequences.

1. pMDC100_*gVenus*

Cacatacaaatggacgaacggataaaccttttcacgcccttttaaatatccgttattctaataaacgctcttttctcttaggtttacccgccaatatatcctg
tcaaacactgatagtttaaactgaaggcgggaaacgacaatctgatccaagctcaagctgctctagcattcgccattcaggctgcgcaactgttgggaagggc
gatcggtgcgggcctcttcgctattacgccagctggcgaaagggggatgtgctgcaaggcgattaagttgggtaacgccagggttttcccagtcacgacgttg
taaaacgacggccagtgccaagcttgcatgcctgcaggtcgactctagaggatccccgggtaccgggccccccctcgaggcgcgccaagcCAAGTTTGTACAA
AAAAGCAGGCTCCACCATGGGAACCAATTCAGTCGACTGGATCCcgacttgccttccgcacaatacatcatttcttcttagctttttttcttcttcttcgttc

atacagtttttttttgtttatcagcttacattttcttgaaccgtagctttcgttttcttctttttaactttccattcggagtttttgtatcttgtttcatagt
ttgtcccaggattagaatgattaggcatcgaaccttcaagaatttgattgaataaaacatcttcattcttaagatatgaagataatcttcaaaaggcccctgg
gaatctgaaagaagagaagcaggcccatttatatgggaaagaacaatagtatttcttatataggcccatttaagttgaaaacaatcttcaaaagtcccacatc
gcttagataagaaaacgaagctgagtttatatacagctagagtcgaagtagtgATTGCGTCGTGACTGGGAAAACCCTGGGTTTtagagctagaaatagcaag
ttaaaataaggctagtccgttatcaacttgaaaaagtggcaccgagtcggtgcttttttttgcaaaattttccagatcgatttcttcttcctctgttcttcgg
cgttcaatttctggggttttctcttcgttttctgtaactgaaacctaaaatttgacctaaaaaaaatctcaaataatatgattcagtggttttgtacttttca
gttagttgagttttgcagttccgatgagataaaccaatattaatccaaactactgcagcctgacagacaaatgaggatgcaaacaattttaaagtttatctaa
cgctagctgttttgtttcttctctctggtgcaccaacgacggcgttttctcaatcataaagaggcttgttttacttaaggccaataatgttgatggatcgaaa
gaagagggctttaataaacgagcccgtttaagctgtaaacgatgtcaaaaacatcccacatcgttcagttgaaaatagaagctctgtttatatattggtaga
gtcgactaagagATTCAGCTGGCGTAATAGCGAAGAGGGTTTtagagctagaaatagcaagttaaaataaggctagtccgttatcaacttgaaaaagtggcac
cgagtcggtgcttttttttgcaaaattttccagatcgatttcttcttcctctgttcttcggcgttcaatttctggggttttctcttcgttttctgtaactgaa
acctaaaatttgacctaaaaaaaatctcaaataatatgattcagtggttttgtacttttcagttagttgagttttgcagttccgatgagataaaccaataCTC
GAGATATCTAGACCCAGCTTTCTTGTACAAAGTGGtcgataattccttaattaactagttctagagcggccgccaccgcggtggagctcgaattcgtaatcat
ggtcatagctgtttcctgtgtgaaattgttatccgctcacaattccacacaacatacgagccggaagcataaagtgtaaagcctggggtgcctaatgagtgag
ctaactcacattaattgcgttgcgctcactgcccgctttccagtcgggaaacctgtcgtgccagctgcattaatgaatcggccaacgcgcggggagaggcggt
ttgcgtattggctagagcagcttgccaacatggtggagcacgacactctcgtctactccaagaatatcaaagatacagtctcagaagaccaaagggctattga
gacttttcaacaaagggtaatatcgggaaacctcctcggattccattgcccagctatctgtcacttcatcaaaaggacagtagaaaaggaaggtggcacctac
aaatgccatcattgcgataaaggaaaggctatcgttcaagatgcctctgccgacagtggtcccaaagatggacccccacccacgaggagcatcgtggaaaaag
aagacgttccaaccacgtcttcaaagcaagtggattgatgtgataacatggtggagcacgacactctcgtctactccaagaatatcaaagatacagtctcaga
agaccaaagggctattgagacttttcaacaaagggtaatatcgggaaacctcctcggattccattgcccagctatctgtcacttcatcaaaaggacagtagaa
aaggaaggtggcacctacaaatgccatcattgcgataaaggaaaggctatcgttcaagatgcctctgccgacagtggtcccaaagatggacccccacccacga
ggagcatcgtggaaaaagaagacgttccaaccacgtcttcaaagcaagtggattgatgtgatatctccactgacgtaagggatgacgcacaatcccactatcc
ttcgcaagaccttcctctatataaggaagttcatttcatttggagaggacacgctgaaatcaccagtctctctctacaaatctatctctctcgagctttcgca
gatctgtcgatcgaccatggggattgaacaagatggattgcacgcaggttctccggccgcttgggtggagaggctattcggctatgactgggcacaacagaca
atcggctgctctgatgccgccgtgttccggctgtcagcgcaggggcgcccggttctttttgtcaagaccgacctgtccggtgccctgaatgaactccaggacg
aggcagcgcggctatcgtggctggccacgacgggcgttccttgcgcagctgtgctcgacgttgtcactgaagcgggaagggactggctgctattgggcgaagt
gccggggcaggatctcctgtcatctcaccttgctcctgccgagaaagtatccatcatggctgatgcaatgcggcggctgcatacgcttgatccggctacctgc
ccattcgaccaccaagcgaaacatcgcatcgagcgagcacgtactcggatggaagccggtcttgtcgatcaggatgatctggacgaagagcatcaggggctcg
cgccagccgaactgttcgccaggctcaaggcgcgcatgcccgacggcgaggatctcgtcgtgacacatggcgatgcctgcttgccgaatatcatggtggaaaa
tggccgcttttctggattcatcgactgtggccggctgggtgtggcggaccgctatcaggacatagcgttggctacccgtgatattgctgaagagcttggcggc
gaatgggctgaccgcttcctcgtgctttacggtatcgccgctcccgattcgcagcgcatcgccttctatcgccttcttgacgagttcttctgagcgggactct
ggggttcggatcgatcctctagctagagtcgatcgacaagctcgagtttctccataataatgtgtgagtagttcccagataagggaattagggttcctatagg
gtttcgctcatgtgttgagcatataagaaacccttagtatgtatttgtatttgtaaaatacttctatcaataaaatttctaattcctaaaaccaaaatccagt
actaaaatccagatcccccgaattaattcggcgttaattcagtacattaaaaacgtccgcaatgtgttattaagttgtctaagcgtcaatttgtttacaccac
aatatatcctgccaccagccagccaacagctccccgaccggcagctcggcacaaaatcaccactcgatacaggcagcccatcagtccgggacggcgtcagcgg
gagagccgttgtaaggcggcagactttgctcatgttaccgatgctattcggaagaacggcaactaagctgccgggtttgaaacacggatgatctcgcggaggg
tagcatgttgattgtaacgatgacagagcgttgctgcctgtgatcaccgcggtttcaaaatcggctccgtcgatactatgttatacgccaactttgaaaacaa
ctttgaaaaagctgttttctggtatttaaggttttagaatgcaaggaacagtgaattggagttcgtcttgttataattagcttcttggggtatctttaaatac
tgtagaaaagaggaaggaaataataaatggctaaaatgagaatatcaccggaattgaaaaaactgatcgaaaaataccgctgcgtaaaagatacggaaggaat
gtctcctgctaaggtatataagctggtgggagaaaatgaaaacctatatttaaaaatgacggacagccggtataaagggaccacctatgatgtggaacgggaa
aaggacatgatgctatggctggaaggaaagctgcctgttccaaaggtcctgcactttgaacggcatgatggctggagcaatctgctcatgagtgaggccgatg
gcgtcctttgctcggaagagtatgaagatgaacaaagccctgaaaagattatcgagctgtatgcggagtgcatcaggctctttcactccatcgacatatcgga
ttgtccctatacgaatagcttagacagccgcttagccgaattggattacttactgaataacgatctggccgatgtggattgcgaaaactgggaagaagacact
ccatttaaagatccgcgcgagctgtatgatttttaaagacggaaaagcccgaagaggaacttgtcttttcccacggcgacctgggagacagcaacatctttg
tgaaagatggcaaagtaagtggctttattgatcttgggagaagcggcagggcggacaagtggtatgacattgccttctgcgtccggtcgatcagggaggatat
cggggaagaacagtatgtcgagctatttttttgacttactggggatcaagcctgattgggagaaaataaaatattatattttactggatgaattgttttagtac
ctagaatgcatgaccaaaatcccttaacgtgagttttcgttccactgagcgtcagaccccgtagaaaagatcaaaggatcttcttgagatcctttttttctgc
gcgtaatctgctgcttgcaaacaaaaaaaccaccgctaccagcggtggtttgtttgccggatcaagagctaccaactctttttccgaaggtaactggcttcag
cagagcgcagataccaaatactgtccttctagtgtagccgtagttaggccaccacttcaagaactctgtagcaccgcctacatacctcgctctgctaatcctg
ttaccagtggctgctgccagtggcgataagtcgtgtcttaccgggttggactcaagacgatagttaccggataaggcgcagcggtcgggctgaacggggggtt
cgtgcacacagcccagcttggagcgaacgacctacaccgaactgagatacctacagcgtgagctatgagaaagcgccacgcttcccgaagggagaaaggcgga
caggtatccggtaagcggcagggtcggaacaggagagcgcacgagggagcttccagggggaaacgcctggtatctttatagtcctgtcgggtttcgccacctc
tgacttgagcgtcgatttttgtgatgctcgtcaggggggcggagcctatggaaaaacgccagcaacgcggcctttttacggttcctggccttttgctggcctt
ttgctcacatgttctttcctgcgttatcccctgattctgtggataaccgtattaccgcctttgagtgagctgataccgctcgccgcagccgaacgaccgagcg
cagcgagtcagtgagcgaggaagcggaagagcgcctgatgcggtattttctccttacgcatctgtgcggtatttcacaccgcatatggtgcactctcagtaca
atctgctctgatgccgcatagttaagccagtatacactccgctatcgctacgtgactgggtcatggctgcgccccgacacccgccaacacccgctgacgcgcc
ctgacgggcttgtctgctcccggcatccgcttacagacaagctgtgaccgtctccgggagctgcatgtgtcagaggttttcaccgtcatcaccgaaacgcgcg
aggcagggtgccttgatgtgggcgccggcggtcgagtggcgacggcgcggcttgtccgcgccctggtagattgcctggccgtaggccagccatttttgagcgg
ccagcggccgcgataggccgacgcgaagcggcggggcgtagggagcgcagcgaccgaagggtaggcgctttttgcagctcttcggctgtgcgctggccagaca
gttatgcacaggccaggcgggttttaagagttttaataagttttaaagagttttaggcggaaaaatcgcctttttttctcttttatatcagtcacttacatgtg
tgaccggttcccaatgtacggctttgggttcccaatgtacgggttccggttcccaatgtacggctttgggttcccaatgtacgtgctatccacaggaaagaga
ccttttcgacctttttcccctgctagggcaatttgccctagcatctgctccgtacattaggaaccggcggatgcttcgccctcgatcaggttgcggtagcgca
tgactaggatcgggccagcctgccccgcctcctccttcaaatcgtactccggcaggtcatttgacccgatcagcttgcgcacggtgaaacagaacttcttgaa
ctctccggcgctgccactgcgttcgtagatcgtcttgaacaaccatctggcttctgccttgcctgcggcgcggcgtgccaggcggtagagaaaacggccgatg
ccgggatcgatcaaaaagtaatcggggtgaaccgtcagcacgtccgggttcttgccttctgtgatctcgcggtacatccaatcagctagctcgatctcgatgt
actccggccgcccggtttcgctctttacgatcttgtagcggctaatcaaggcttcaccctcggataccgtcaccaggcggccgttcttggccttcttcgtacg
ctgcatggcaacgtgcgtggtgtttaaccgaatgcaggtttctaccaggtcgtctttctgctttccgccatcggctcgccggcagaacttgagtacgtccgca
acgtgtggacggaacacgcggccgggcttgtctcccttcccttcccggtatcggttcatggattcggttagatgggaaaccgccatcagtaccaggtcgtaat
cccacacactggccatgccggccggccctgcggaaacctctacgtgcccgtctggaagctcgtagcggatcacctcgccagctcgtcggtcacgcttcgacag
acggaaaacggccacgtccatgatgctgcgactatcgcgggtgcccacgtcatagagcatcggaacgaaaaaatctggttgctcgtcgcccttgggcggcttc
ctaatcgacggcgcaccggctgccggcggttgccgggattctttgcggattcgatcagcggccgcttgccacgattcaccggggcgtgcttctgcctcgatgc
gttgccgctgggcggcctgcgcggccttcaacttctccaccaggtcatcacccagcgccgcgccgatttgtaccgggccggatggtttgcgaccgtcacgccg
attcctcggcttggggggttccagtgccattgcagggccggcagacaacccagccgcttacgcctggccaaccgcccgttcctccacacatggggcattccac
ggcgtcggtgcctggttgttcttgattttccatgccgcctcctttagccgctaaaattcatctactcatttattcatttgctcatttactctggtagctgcgc
gatgtattcagatagcagctcggtaatggtcttgccttggcgtaccgcgtacatcttcagcttggtgtgatcctccgccggcaactgaaagttgacccgcttc
atggctggcgtgtctgccaggctggccaacgttgcagccttgctgctgcgtgcgctcggacggccggcacttagcgtgtttgtgcttttgctcattttctctt
tacctcattaactcaaatgagttttgatttaatttcagcggccagcgcctggacctcgcgggcagcgtcgccctcgggttctgattcaagaacggttgtgccg
gcggcggcagtgcctgggtagctcacgcgctgcgtgatacgggactcaagaatgggcagctcgtacccggccagcgcctcggcaacctcaccgccgatgcgcg
tgcctttgatcgcccgcgacacgacaaaggccgcttgtagccttccatccgtgacctcaatgcgctgcttaaccagctccaccaggtcggcggtggcccatat

gtcgtaagggcttggctgcaccggaatcagcacgaagtcggctgccttgatcgcggacacagccaagtccgccgcctggggcgctccgtcgatcactacgaag
tcgcgccggccgatggccttcacgtcgcggtcaatcgtcgggcggtcgatgccgacaacggttagcggttgatcttcccgcacggccgcccaatcgcgggcac
tgccctggggatcggaatcgactaacagaacatcggccccggcgagttgcagggcgcgggctagatgggttgcgatggtcgtcttgcctgacccgcctttctg
gttaagtacagcgataaccttcatgcgttccccttgcgtatttgtttatttactcatcgcatcatatacgcagcgaccgcatgacgcaagctgttttactcaa
atacacatcaccttttagacggcggcgctcggtttcttcagcggccaagctggccggccaggccgccagcttggcatcagacaaaccggccaggatttcatg
cagccgcacggttgagacgtgcgcgggcggctcgaacacgtacccggccgcgatcatctccgcctcgatctcttcggtaatgaaaaacggttcgtcctggccg
tcctggtgcggtttcatgcttgttcctcttggcgttcattctcggcggccgccagggcgtcggcctcggtcaatgcgtcctcacggaaggcaccgcgccgcct
ggcctcggtgggcgtcacttcctcgctgcgctcaagtgcgcggtacagggtcgagcgatgcacgccaagcagtgcagccgcctctttcacggtgcggccttcc
tggtcgatcagctcgcgggcgtgcgcgatctgtgccggggtgagggtagggcgggggccaaacttcacgcctcgggccttggcggcctcgcgcccgctccggg
tgcggtcgatgattagggaacgctcgaactcggcaatgccggcgaacacggtcaacaccatgcggccggccggcgtggtggtgtcggcccacggctctgccag
gctacgcaggcccgcgccggcctcctggatgcgctcggcaatgtccagtaggtcgcgggtgctgcgggccaggcggtctagcctggtcactgtcacaacgtcg
ccagggcgtaggtggtcaagcatcctggccagctccgggcggtcgcgcctggtgccggtgatcttctcggaaaacagcttggtgcagccggccgcgtgcagtt
cggcccgttggttggtcaagtcctggtcgtcggtgctgacgcgggcatagcccagcaggccagcggcggcgctcttgttcatggcgtaatgtctccggttcta
gtcgcaagtattctactttatgcgactaaaacacgcgacaagaaaacgccaggaaaagggcagggcggcagcctgtcgcgtaacttaggacttgtgcgacatg
tcgttttcagaagacggctgcactgaacgtcagaagccgactgcactatagcagcggaggggttggatcaaagtactttgatcccgaggggaaccctgtggtt
ggcatg

2. pMDC100_*gVenus-tRNA*^{Met} *(TLS1)*

cacatacaaatggacgaacggataaaccttttcacgcccttttaaatatccgttattctaataaacgctcttttctcttaggtttacccgccaatatatcctg
tcaaacactgatagtttaaactgaaggcgggaaacgacaatctgatccaagctcaagctgctctagcattcgccattcaggctgcgcaactgttgggaagggc
gatcggtgcgggcctcttcgctattacgccagctggcgaaagggggatgtgctgcaaggcgattaagttgggtaacgccagggttttcccagtcacgacgttg
taaaacgacggccagtgccaagcttgcatgcctgcaggtcgactctagaggatccccgggtaccgggccccccctcgaggcgcgccaagcCAAGTTTGTACAA
AAAAGCAGGCTCCACCATGGGAACCAATTCAGTCGACTGGATCCcgacttgccttccgcacaatacatcatttcttcttagctttttttcttcttcttcgttc
atacagtttttttttgtttatcagcttacattttcttgaaccgtagctttcgttttcttctttttaactttccattcggagtttttgtatcttgtttcatagt
ttgtcccaggattagaatgattaggcatcgaaccttcaagaatttgattgaataaaacatcttcattcttaagatatgaagataatcttcaaaaggcccctgg
gaatctgaaagaagagaagcaggcccatttatatgggaaagaacaatagtatttcttatataggcccatttaagttgaaaacaatcttcaaaagtcccacatc
gcttagataagaaaacgaagctgagtttatatacagctagagtcgaagtagtgATTGCGTCGTGACTGGGAAAACCCTGGGTTTtagagctagaaatagcaag
ttaaaataaggctagtccgttatcaacttgaaaaagtggcaccgagtcggtgcATTATCAGAGTGGCGCAGCGGAAGCGTGGTGGGCCCATAACCCACAGGTC
CCAGGATCGAAACCTGGCTCTGATA**AAAAAAAA**ttttttttgcaaaattttccagatcgatttcttcttcctctgttcttcggcgttcaatttctggggtttt
ctcttcgttttctgtaactgaaacctaaaatttgacctaaaaaaaatctcaaataatatgattcagtggttttgtacttttcagttagttgagttttgcagtt
ccgatgagataaaccaatattaatccaaactactgcagcctgacagacaaatgaggatgcaaacaattttaaagtttatctaacgctagctgttttgtttctt
ctctctggtgcaccaacgacggcgttttctcaatcataaagaggcttgttttacttaaggccaataatgttgatggatcgaaagaagagggcttttaataaac
gagcccgtttaagctgtaaacgatgtcaaaaacatcccacatcgttcagttgaaaatagaagctctgtttatatattggtagagtcgactaagagATTCAGCT
GGCGTAATAGCGAAGAGGGTTTtagagctagaaatagcaagttaaaataaggctagtccgttatcaacttgaaaaagtggcaccgagtcggtgcATTATCAGA
GTGGCGCAGCGGAAGCGTGGTGGGCCCATAACCCACAGGTCCCAGGATCGAAACCTGGCTCTGATA**AAAAAAAA**ttttttttgcaaaattttccagatcgatt
tcttcttcctctgttcttcggcgttcaatttctggggttttctcttcgttttctgtaactgaaacctaaaatttgacctaaaaaaaatctcaaataatatgat
tcagtggttttgtacttttcagttagttgagttttgcagttccgatgagataaaccaataCTCGAGATATCTAGACCCAGCTTTCTTGTACAAAGTGGtcgat
aattccttaattaactagttctagagcggccgccaccgcggtggagctcgaattcgtaatcatggtcatagctgtttcctgtgtgaaattgttatccgctcac
aattccacacaacatacgagccggaagcataaagtgtaaagcctggggtgcctaatgagtgagctaactcacattaattgcgttgcgctcactgcccgctttc
cagtcgggaaacctgtcgtgccagctgcattaatgaatcggccaacgcgcggggagaggcggtttgcgtattggctagagcagcttgccaacatggtggagca
cgacactctcgtctactccaagaatatcaaagatacagtctcagaagaccaaagggctattgagacttttcaacaaagggtaatatcgggaaacctcctcgga
ttccattgcccagctatctgtcacttcatcaaaaggacagtagaaaaggaaggtggcacctacaaatgccatcattgcgataaaggaaaggctatcgttcaag
atgcctctgccgacagtggtcccaaagatggacccccacccacgaggagcatcgtggaaaaagaagacgttccaaccacgtcttcaaagcaagtggattgatg
tgataacatggtggagcacgacactctcgtctactccaagaatatcaaagatacagtctcagaagaccaaagggctattgagacttttcaacaaagggtaata
tcgggaaacctcctcggattccattgcccagctatctgtcacttcatcaaaaggacagtagaaaaggaaggtggcacctacaaatgccatcattgcgataaag
gaaaggctatcgttcaagatgcctctgccgacagtggtcccaaagatggacccccacccacgaggagcatcgtggaaaaagaagacgttccaaccacgtcttc
aaagcaagtggattgatgtgatatctccactgacgtaagggatgacgcacaatcccactatccttcgcaagacccttcctctatataaggaagttcatttcatt
tggagaggacacgctgaaatcaccagtctctctctacaaatctatctctctcgagctttcgcagatctgtcgatcgaccatggggattgaacaagatggattg
cacgcaggttctccggccgcttgggtggagaggctattcggctatgactgggcacaacagacaatcggctgctctgatgccgccgtgttccggctgtcagcgc
aggggcgcccggttctttttgtcaagaccgacctgtccggtgccctgaatgaactccaggacgaggcagcgcggctatcgtggctggccacgacgggcgttcc
ttgcgcagctgtgctcgacgttgtcactgaagcgggaagggactggctgctattgggcgaagtgccggggcaggatctcctgtcatctcaccttgctcctgcc
gagaaagtatccatcatggctgatgcaatgcggcggctgcatacgcttgatccggctacctgcccattcgaccaccaagcgaaacatcgcatcgagcgagcac
gtactcggatggaagccggtcttgtcgatcaggatgatctggacgaagagcatcaggggctcgcgccagccgaactgttcgccaggctcaaggcgcgcatgcc
cgacggcgaggatctcgtcgtgacacatggcgatgcctgcttgccgaatatcatggtggaaaatggccgcttttctggattcatcgactgtggccggctgggt
gtggcggaccgctatcaggacatagcgttggctacccgtgatattgctgaagagcttggcggcgaatgggctgaccgcttcctcgtgctttacggtatcgccg
ctcccgattcgcagcgcatcgccttctatcgccttcttgacgagttcttctgagcgggact…………….

3. pMDC100_*gVenus-tRNA*$^{Met\ \Delta DT}$*(TLS2)*

cacatacaaatggacgaacggataaaccttttcacgcccttttaaatatccgttattctaataaacgctcttttctcttaggtttacccgccaatatatcctg
tcaaacactgatagtttaaactgaaggcgggaaacgacaatctgatccaagctcaagctgctctagcattcgccattcaggctgcgcaactgttgggaagggc
gatcggtgcgggcctcttcgctattacgccagctggcgaaagggggatgtgctgcaaggcgattaagttgggtaacgccagggttttcccagtcacgacgttg
taaaacgacggccagtgccaagcttgcatgcctgcaggtcgactctagaggatccccgggtaccgggccccccctcgaggcgcgccaagcCAAGTTTGTACAA
AAAAGCAGGCTCCACCATGGGAACCAATTCAGTCGACTGGATCCcgacttgccttccgcacaatacatcatttcttcttagctttttttcttcttcttcgttc
atacagtttttttttgtttatcagcttacattttcttgaaccgtagctttcgttttcttctttttaactttccattcggagtttttgtatcttgtttcatagt
ttgtcccaggattagaatgattaggcatcgaaccttcaagaatttgattgaataaaacatcttcattcttaagatatgaagataatcttcaaaaggcccctgg
gaatctgaaagaagagaagcaggcccatttatatgggaaagaacaatagtatttcttatataggcccatttaagttgaaaacaatcttcaaaagtcccacatc
gcttagataagaaaacgaagctgagtttatatacagctagagtcgaagtagtgATTGCGTCGTGACTGGGAAAACCCTGGGTTTtagagctagaaatagcaag
ttaaaataaggctagtccgttatcaacttgaaaaagtggcaccgagtcggtgcATTATCAGAGTGGTGGTGGGCCCATAACCCACAGGTCCGCTCTGATA**AAA**
AAAAAttttttttgcaaaattttccagatcgatttcttcttcctctgttcttcggcgttcaatttctggggttttctcttcgttttctgtaactgaaacctaa
aatttgacctaaaaaaaatctcaaataatatgattcagtggttttgtacttttcagttagttgagttttgcagttccgatgagataaaccaatattaatccaa
actactgcagcctgacagacaaatgaggatgcaaacaattttaaagtttatctaacgctagctgttttgtttcttctctctggtgcaccaacgacggcgtttt
ctcaatcataaagaggcttgttttacttaaggccaataatgttgatggatcgaaagaagagggcttttaataaacgagcccgtttaagctgtaaacgatgtca
aaaacatcccacatcgttcagttgaaaatagaagctctgtttatatattggtagagtcgactaagagATTCAGCTGGCGTAATAGCGAAGAGGGTTTtagagc

tagaaatagcaagttaaaataaggctagtccgttatcaacttgaaaaagtggcaccgagtcggtgcATTATCAGAGTGGTGGTGGCCCATAACCCACAGGTC
CGCTCTGATA**AAAAAAAA**tttttttgcaaaattttccagatcgatttcttcttcctctgttcttcggcgttcaatttctggggttttctcttcgttttctgt
aactgaaacctaaaatttgacctaaaaaaaatctcaaataatatgattcagtggttttgtacttttcagttagttgagttttgcagttccgatgagataaacc
aataCTCGAGATATCTAGACCCAGCTTTCTTGTACAAAGTGGtcgataattccttaattaactagttctagagcggccgccaccgcggtggagctcgaattcg
taatcatggtcatagctgtttcctgtgtgaaattgttatccgctcacaattccacacaacatacgagccggaagcataaagtgtaaagcctggggtgcctaat
gagtgagctaactcacattaattgcgttgcgctcactgcccgctttccagtcgggaaacctgtcgtgccagctgcattaatgaatcggccaacgcgcggggag
aggcggtttgcgtattggctagagcagcttgccaacatggtggagcacgacactctcgtctactccaagaatatcaaagatacagtctcagaagaccaaaggg
ctattgagacttttcaacaaagggtaatatcgggaaacctcctcggattccattgcccagctatctgtcacttcatcaaaaggacagtagaaaaggaaggtgg
cacctacaaatgccatcattgcgataaaggaaaggctatcgttcaagatgcctctgccgacagtggtcccaaagatggacccccacccacgaggagcatcgtg
gaaaaagaagacgttccaaccacgtcttcaaagcaagtggattgatgtgataacatggtggagcacgacactctcgtctactccaagaatatcaaagatacag
tctcagaagaccaaagggctattgagacttttcaacaaagggtaatatcgggaaacctcctcggattccattgcccagctatctgtcacttcatcaaaaggac
agtagaaaaggaaggtggcacctacaaatgccatcattgcgataaaggaaaggctatcgttcaagatgcctctgccgacagtggtcccaaagatggaccccca
cccacgaggagcatcgtggaaaaagaagacgttccaaccacgtcttcaaagcaagtggattgatgtgatatctccactgacgtaagggatgacgcacaatccc
actatccttcgcaagaccttcctctatataaggaagttcatttcatttggagaggacacgctgaaatcaccagtctctctctacaaatctatctctctcgagc
tttcgcagatctgtcgatcgaccatggggattgaacaagatggattgcacgcaggttctccggccgcttgggtggagaggctattcggctatgactgggcaca
acagacaatcggctgctctgatgccgccgtgttccggctgtcagcgcaggggcgcccggttctttttgtcaagaccgacctgtccggtgccctgaatgaactc
caggacgaggcagcgcggctatcgtggctggccacgacgggcgttccttgcgcagctgtgctcgacgttgtcactgaagcgggaagggactggctgctattgg
gcgaagtgccggggcaggatctcctgtcatctcaccttgctcctgccgagaaagtatccatcatggctgatgcaatgcggcggctgcatacgcttgatccggc
tacctgcccattcgaccaccaagcgaaacatcgcatcgagcgagcacgtactcggatggaagccggtcttgtcgatcaggatgatctggacgaagagcatcag
gggctcgcgccagccgaactgttcgccaggctcaaggcgcgcatgcccgacggcgaggatctcgtcgtgacacatggcgatgcctgcttgccgaatatcatgg
tggaaaatggccgcttttctggattcatcgactgtggccggctgggtgtggcggaccgctatcaggacatagcgttggctacccgtgatattgctgaagagct
tggcggcgaatgggctgaccgcttcctcgtgctttacggtatcgccgctcccgattcgcagcgcatcgccttctatcgccttcttgacgagttcttctgagcg
ggact……………

d, Sequence of the *35S$_{promoter}$::H2B-Venus::35S$_{terminator}$::Basta* construct in the binary Pri-909 vector used in the study.

Light Grey colour background indicates *35S* promoter; Green colour background indicates *H2B-Venus gene* sequence. Dark Grey colour background indicates *35S* terminator. Red colour background indicates two *Venus* target sequences. Light blue colour background indicates *BlpR Basta Resistance* gene sequence; Dark Yellow colour background indicates *NOS* terminator sequence.

TGAGCAAAAGGCCAGCAAAAGGCCAGGAACCGTAAAAAGGCCGCGTTGCTGGCGTTTTTCCATAGGCTCCGCCCCCCTGACGAGCATCACAAAAATCGACGCT
CAAGTCAGAGGTGGCGAAACCCGACAGGACTATAAAGATACCAGGCGTTTCCCCCTGGAAGCTCCCTCGTGCGCTCTCCTGTTCCGACCCTGCCGCTTACCGG
ATACCTGTCCGCCTTTCTCCCTTCGGGAAGCGTGGCGCTTTCTCATAGCTCACGCTGTAGGTATCTCAGTTCGGTGTAGGTCGTTCGCTCCAAGCTGGGCTGT
GTGCACGAACCCCCCGTTCAGCCCGACCGCTGCGCCTTATCCGGTAACTATCGTCTTGAGTCCAACCCGGTAAGACACGACTTATCGCCACTGGCAGCAGCCA
CTGGTAACAGGATTAGCAGAGCGAGGTATGTAGGCGGTGCTACAGAGTTCTTGAAGTGGTGGCCTAACTACGGCTACACTAGAAGAACAGTATTTGGTATCTG
CGCTCTGCTGAAGCCAGTTACCTTCGGAAAAAGAGTTGGTAGCTCTTGATCCGGCAAACAAACCACCGCTGGTAGCGGTGGTTTTTTTGTTTGCAAGCAGCAG
ATTACGCGCAGAAAAAAAGGATCTCAAGAAGATCCTTTGATCTTTTCTACGGGGTCTGACGCTCAGTGGAACGAAAACTCACGTTAAGGGATTTTGGTCATGA
GATTATCAAAAAGGATCTTCACCTAGATCCTTTTAAATTAAAAATGAAGTTTTAAATCAATCTAAAGTATATATGAGTAAACTTGGTCTGACAGTTACCAATG
CTTAATCAGTGAGGCACCTATCTCAGCGATCTGTCTATTTCGTTCATCCATAGTTGCCTGACTCGATCCTACAAGGTAGAATCCGCCTGAGTCGCAAGGGTGA
CTTCGCCTATATTGGACGACGGCGCGCAGAGGGCGACCTCTTTTTGGGTTACGATTGTAGGATTATCACTAAAACAATACATGAACATATTCAAATGGCAATC
TCTCTAAGGCATTGGAAATAAATACAAATAACAGTTGGGTGGAGTTTTCGACCTGAGGGCGTTAACCTTCTGTTAACCTAAAAGCTCTTGCCCAAACAGCAG
AATCGGCGCTAATTGCCAGCGGCGGAACTTTTCCAGTTTCGCGAAAAATATCGCCACTGGCAAGGAATGGGTTTGAGATGGCGAAGTCTGTCCTAAAAGCAGC
GCCTGTAGTTGTAGGGTTGACGGCCTTGATGGAGCGTCATGCCGATGCCCTCTCGAGCCAACTTCAAGCACATCATCTTAAGGTTTTCCCGCCGCATTCCGAG
AAGGGTATTCGAACATTCGGGCCATCGGAGGCGTCCAAGCTGCTCGGCGTTGGCGAGTCATATTTACGGCAGACCGCGTCTGAGATGCCAGAGTTGAATGTTA
GCATGAGCCCAGGTGGCAGGCGAATGTTCTCAATTGAAGATATCCATGTGATTCGGAAGTATATGGATCAGGTCGGCCGCGGGAACCGGCGCTACCTGCCACA
TCGTCGAGGCGGCGAGCAGCTTCAGGTTATCTCTGTGATGAATTTCAAAGGTGGGTCGGGTAAGACCACCACCGCCGCGCATCTGGCGCAGTACCTCGCTATG
CGCGGATATCGAGTCTTGGCCATTGATCTCGATCCTCAAGCGAGCCTTTCTGCACTCTTTGGGAGCCAACCGGAGACGGACGTTGGCCCGAACGAAACGCTCT
ACGGCGCTATAAGGTATGATGATGAGCAGGTGGCAATCGAACGAGTCGTCCGAGGGACTTACATTCCCGACCTCCACCTGATTCCTGGTAACCTTGAGCTGAT
GGAGTTTGAACACGATACGCCACGCGCGCTGATGAACCGCAAAGAGGGCGACACGCTCTTTTATGGTCGCATCAGCCAAGTAATTGAAGATATCGCGGATAAC
TATGACGTCGTGGTCATCGACTGCCCTCCCCAGCTTGGGTATCTCACGCTATCCGCATTGACTGCGGCGACGTCCATTCTTGTCACGGTCCATCCGCAGATGC
TGGATGTGATGTCGATGAACCAGTTTCTGGCAATGACATCGAACCTTTTGCGTGAAATCGAGAATGCTGGCGCCAAGTTCAAGTTTAATTGGATGCGCTATCT
GATAACCCGTTTCGAACCGAGCGACGGACCACAGAACCAAATGGTAGGTTATCTGCGGTCGATTTTTGGCGAAAATGTCCTCAATTTTCCGATGCTTAAAACC
ACCGCGGTTTCGGACGCTGGCCTGACAAACCAGACTCTATTCGAAGTGGAGCGTGGCCTGTTCACGCGCTCGACCTATGATCGAGCCTTGGAGGCGATGAACG
CCGTCAACGACGAGATCGAAACACTGATCAAAAAAGCATGGGGTAGGCCCACATGAGCCGGAAGCACATCCTTGGCGTCTCAACTGACGCCCCTGAGACGTCG
CCCGCCGACAATAGGACGGCAAAGAACCGCTCCATGCCGCTCCTCGGCGTAACAAGGAAGGAGCGCGATCCGGCAACGAAGCTCACAGCGAACATTGGTAACG
CACTGCGAGAGCAAAACGATCGTCTTAGCCGTGCCGAAGAGATCGAGCGGCGTCTCGCTGAAGGTCAGGCAGTGATAGAGTTGGATGCCTCGTCAATAGAACC
GTCTTTCGTGCAGGATCGTATGCGAGGGGACATTGACGGGCTCCTTACTTCGATCCGGGAACAAGGACAGCAAGTCCCAATCCTTGTGCGACCGCATCCGAGC
CAGCCGGGCCGATATCAGGTTGCCTTCGGCCACCGCCGGCTACGCGCCGTTTCAGAACTCGGACTTCCGGTCAGGGCGGTCGTTCGCGAACTGACGGACGAGC
AAGTGGTCGTAGCACAGGGTCAGGAAAACAATGTGCGCGAAGATCTTACCTTCATCGAAAAGGCGCGCTTCGCACATCGCCTGAACAGGCAGTTTTCTCGAGA
GATTGTCATCGCCGCGATGTCGATCGACAAGAGCAATTTGTCCAAGATGCTTCTGCTCGTTGACGCCCTCCCCTCTGAACTGACCGATGCTATTGGTGCCGCT
CCTGGTGTTGGACGGCCGAGTTGGCAACAACTTGCCGAGCTGATTGAGAAAGTTTCTTCACCGGCCGACGTGGCTAAATATGCTATGTCGGAGGAAGTTCAAG
CGCTGCCATCGGCAGAACGATTCAAGGCGGTGATCGCTAGTCTGAAGCCCAGTCGGGTTGCGCGTGGACTTCCCGAGGTCATGGCCACCCCAGACGGCACCAG
AATTGCACAGGTGACGCAGAGCAAGGCCAAACTGGAAATCACGATTGACAGGAAGGCGACGCCCGATTTTGCGACCTTCGTGCTCGATCATGTGCCAGCGCTG
TATCAAGCGTACCACGCTGAGAACCAACGGAAACGGGGAGAGTAAACCGCAAAAGAAAAGAGCCCCCTCAACGTCGCCGTCGCGGAAGCCCTTCTGTCTCTCT
AGCGCGAACAGAATCGCATTTCCTCGAATCCTCGTCAAGAGTTTTTAGCGCCGTTTTGGTGAGCTGATTTCCTTTGCCTGCTGAAAGGTGAAAGATGATGCAG
ACAGGAAGTGTAACGACGCCATTCGGGCGGCGGCCAATGACGCTTGCGCTTGTGCGGCGCCAGACGGCGCTGGCCGATATCAAACAAGGCAAGACAGCGGACA
AGTGGAAGGTCTTTAGGACGCGTCCGCGGCCATGGAACTACTTGGAATCCAGTCCAACAGTCTTGCCGTCCTTGATGCGCTATTGAGCTTTCACCCGGAAAC

GGAGTTGCGTCAGGAGGCACAGCTGATCGTCTTCCCGTCGAATGCTCAGCTTGCCCTTCGGGCGCATGGGATGGCTGGCGCGACTTTGCGTAGGCACATCGCC
ATGCTCGTGGAGTCAGGCTTGATCGTCCGGAAGGATAGCGCCAACGGAAAGCGTTACGCTCGTAAGGATGGCGCTGGTCAGATCGAGCGCGCGTTTGGCTTCG
ATTTGTCTCCGCTTCTCGCGCGGTCCGAAGAGCTAGCGATGATGGCACAGCAGGTGATGGCCGATCGAGCAGCATTCAGGATGGCCAAAGAAAGTCTGACGAT
TTGCCGACGGGACGTTCGGAAGCTAATTACGGCAGCTATGGAAGAGGGAGCGGAGGGCGACTGGCAAGCTGTCGAGGAAGTCTATGTGGAACTTGTGGGTAGA
ATCCCACGCGCCCCGACGCTTGCTGATGTAGAGTCAATTCTCGAAGAGATGTGGATGCTCCAGGAAGAGATAATCAACCGGTTGGAAATTAGAGACAATTCAG
AAAATAATAGCACCAATGCTGCCCAGAGCGAGCAGCACATACAGAACTCAAAACCCGAATCCGTTAATGAACTTGAACCTCGCTCTGAAAAGGAGCAGGGCGC
TAAGCCGAGTGAAATAGACCGGGCAAGGAGCGAGCCGATAAAAGCGTTCCCCCTCGGGATGATCCTGAAAGCGTGCCCGACCATTGGCAATTATGGGCCGAGC
GGTGCGGTTGCTAGCTGGCGTGACCTCATGTCGGCTGCGGTGGTGGTTCGGTCTATGCTGGGGGTCAGCCCGTCGGCTTACCAAGACGCGTGTGAGGCAATGG
GACCGGAGAATGCGGCAGCAGCGATGGCGTGCATTTTGGAGCGAGCGAACTTCATCAATTCGCCTGGGGGCTATCTCCGAGATCTGACACGGCGGAGCGAACT
CGGGAAGTTTTCACTTGGCCCGATGATAATGGCGCTCTTGAAGGCTAGCGGGCAGGGGACGTTGCGGTTTGGCTAGAATTAGCGAGTATGGGGAGCAGGATGGTC
TGTGGTCAGCTGACCACAGACCTAATAGGTTGAAAACATGAGCGTTTTTTGGATGATCGACAGACCATCCGATTCCCGGAGTACCAAGCGTGCTCTGATGGGA
GCGATAACATTACTCAACAAGCACGAAGGCCCCATGCCGATCGTTGATCGTGAAGGAGAGCCTGCTCTACATGCGGCGGTATTTTGCCGGCCGAGGCATGTAG
TCGCGGAGCACTGCCTATTTACTGCCCTAGGCACAAACGTTGACTCTTGGATCGAGCTGGCAGACAAAGCAATAACCCCACACAGAGGACGATTAATGGCTGAC
GAAGAGATCCAGAATCCGCCGGACGGTACTGCTGCTGCCGAAGTTGAGCCGGCTGCTCCTAGAGGTAGAAGAGCAAAGAAAGCACCAGCCGAAACAGCCCCGCA
CGGGATCGTTCAAATCCGTGAAGCCGAAAACCCGCGGCCTCAGCAACCGAGAAAAACTGGAGAAGATCGGTCAAATCGAAGCTCAGGTCGCTGGCGGCGCAAC
CTTGAAGGACGCCCGTTAAGATCGTGGGTATTTCCGTTCAGACCTATTATCAATGGAAGAGAGCTGCGGTTCAACCTGTCTCACAGAATCCGGCCGTGTCTGTT
TCAGTTGACGATGAACTCGGCGAGTTCATCCAACTCGAGGAGGAAAATCGGCGGCTCAGAAAGCTAGCTTGTGGCAGGATATATTGTGGTGTAAACAAATTGA
CGCTTAGACAACTTAATAACACATTGCGGACGTTTTTAATGTACTGGGGTGGTTTTTCTTTTCACCAGTGAGACGGGCAACAGCTGATTGCCCTTCACCGCCT
GGCCCTGAGAGAGTTGCAGCAAGCGGTCCACGCTGGTTTGCCCCAGCAGGCGAAAATCCTGTTTGATGGTGGTTCCGAAATCGGCAAAATCCCTTATAAATCA
AAAGAATAGCCCGAGATAGGGTTGAGTGTTGTTCCAGTTTGGAACAAGAGTCCACTATTAAAGAACGTGGACTCCAACGTCAAAGGGCGAAAAACCGTCTATC
AGGGCGATGGCCCAAATTCCCGATCTAGTAACATAGATGACACCGCGCGCGATAATTTATCCTAGTTTGCGCGCTATATTTTGTTTTCTATCGCGTATTAAAT
GTATAATTGCGGGACTCTAATCATAAAAACCCATCTCATAAATAACGTCATGCATTACATGTTAATTATTACATGCTTAACGTAATTCAACAGAAATTATATG
ATAATCATCGCAAGACCGGCAACAGGATTCAATCTTAAGAAACTTTATTGCCAAATGTTTGAACGATCGGGGCCCGATCATATTGTCGCTCAGGATCGTGGCG
TTGTGCTTGTCGGCCGTTGCTGTCGTAATGATATCGGCACCTTCGACCGCCTGTTCCGCAGAGATCCCGTGGGCGAAGAACTCCAGCATGAGATCCCCGCGCT
GGAGGATCATCCAGCCGGCGTCCCGGAAAACGATTCCGAAGCCCAACCTTTCATAGAAGGCGGCGGTGGAATCGAAATCTCGTGATGGCAGGTTGGGCGTCGC
TTGGTCGGTCATTTCGAACCCCAGAGTCCCGCTCAGAAGAACTCGTCAAGAAGGCGATAGAAGGCGATGCGCTGCGAATCGGGAGCGGCGATACCGTAAAGCA
CGAGGAAGCGGTCAGCCCATTCGCCGCCAAGCTCTTCAGCAATATCACGGGTAGCCAACGCTATGTCCTGATAGCGGTCCGCCACACCCAGCCGGCCACAGTC
GATGAATCCAGAAAAGCGGCCATTTTCCACCATGATATTCGGCAAGCAGGCATCGCCATGGGTCACGACGAGATCATCGCCGTCGGGCATACGCGCCTTGAGC
CTGGCGAACAGTTCGGCTGGCGCGAGCCCCTGATGCTCTTCGTCCAGATCATCCTGATCGACAAGACCGGCTTCCATCCGAGTACGTGCTCGCTCGATGCGAT
GTTTCGCTTGGTGGTCGAATGGGCAGGTAGCCGGATCAAGCGTATGCAGCCGCCGCATTGCATCAGCCATGATGGATACTTTCTCGGCAGGAGCAAGGTGAGA
TGACAGGAGATCCTGCCCCGGCACTTCGCCCAATAGCAGCCAGTCCCTTCCCGCTTCAGTGACAACGTCGAGCACAGCTGCGCAAGGAACGCCCGTCGTGGCC
AGCCACGATAGCCGCGCTGCCTCGTCCTGTAGTTCATTCAGGGCACCGGACAGGTCGGTCTTGACAAAAAGAACCGGGCGCCCCTGCGCTGACAGCCGGAACA
CGGCGGCATCAGAGCAGCCGATTGTCTGTTGTGCCCAGTCATAGCCGAATAGCCTCTCCACCCAAGCGGCCGGAGAACCTGCGTGCAATCCATCTTGTTCAAT
CATGCGAAACGATCCAGATCCGGTGCAGATTATTTGGATTGAGAGTGAATATGAGACTCTAATTGGATACCGAGGGGAATTTATGGAACGTCAGTGGAGCATT
TTTGACAAGAAATATTTGCTAGCTGATAGTGACCTTAGGCGACTTTTGAACGCGCAATAATGGTTTCTGACGTATGTGCTTAGCTCATTAAACTCCAGAAACC
CGCGGCTGAGTGGCTCCTTCAACGTTGCGGTTCTGTCAGTTCCAAACGTAAAACGGCTTGTCCCGCGTCATCGGCGGGGGTCATAACGTGACTCCCTTAATTC
TCCGCTCATGATCCTGGCGAAAGGGGGATGTGCTGCAAGGCGATTAAGTTGGGTAACGCCAGGGTTTTCCCAGTCACGACGTTGTAAAACGACGGCCAGTGCC
AAGCTTGCATGCCTGCAGGTCGACTCTAGAGGATCCTGAGACTTTTCAACAAAGGATAATTTCGGGAAACCTCCTCGGATTCCATTGCCCAGCTATCTGTCAC
TTCATCGAAAGGACAGTAGAAAAGGAAGGTGGCTCCTACAAATGCCATCATTGCGATAAAGGAAAGGCTATCATTCAAGATCTCTCTGCCGACAGTGGTCCCA
AAGATGGACCCCCACCCACGAGGAGCATCGTGGAAAAAGAAGAGGTTCCAACCACGTCTACAAAGCAAGTGGATTGATGTGACATCTCCACTGACGTAAGGGA
TGACGCACAATCCCACTATCCTTCGCAAGACCCTTCCTCTATATAAGGAAGTTCATTTCATTTGGAGAGGACACGCTCGAGTATAAGAGCTCATTTTTACAAC
AATTACCAACAACAACAAACAACAAACAACATTACAATTACATTTACAATTATCGATACACGTCGTGACTGGGAAAACCCTGGATGGCGAAAGCGGATAAAAA
ACCGGCGGAAAAAAACCGGCGGAAAAAAACCCCGGCGGCGGAACCGGCGGCGGCGGCGGAAAAAAAACCGAAAGCGGGCAAAAAACTGCCGAAAGAACCGGCG
GGCGCGGGCGATAAAAAAAAAAAACGCAGCAAAAAAAACGTGGAAACCTATAAAATTTATATTTTTAAAGTGCTGAAACAGGTGCATCCGGATATTGGCATTA
GCAGCAAAGCGATGGGCATTATGAACAGCTTTATTAACGATATTTTTGAAAAACTGGCGGGCGAAAGCAGCAAACTGGCGCGCTATAACAAAAAACCGACCAT
TACCAGCCGCGAAATTCAGACCGCGGTGCGCCTGGTGCTGCCGGGCGAACTGGCGAAACATGCGGTGAGCGAAGGCACCAAAGCGGTGACCAAATTTACCAGC
ATGGTGAGCAAAGGCGAAGAACTGTTTACCGGCGTGGTGCCGATTCTGGTGGAACTGGATGGCGATGTGAACGGCCATAAATTTAGCGTGAGCGGCGAAGGCG
AAGGCGATGCGACCTATGGCAAACTGACCCTGAAACTGATTTGCACCACCGGCAAACTGCCGGTGCCGTGGCCGACCCTGGTGACCACCCTGGGCTATGGCCT
GCAGTGCTTTGCGCGCTATCCGGATCATATGAAACAGCATGATTTTTTTAAAAGCGCGATGCCGGAAGGCTATGTGCAGGAACGCACCATTTTTTTTAAAGAT
GATGGCAACTATAAAACCCGCGCGGAAGTGAAATTTGAAGGCGATACCCTGGTGAACCGCATTGAACTGAAAGGCATTGATTTTAAAGAAGATGGCAACATTC
TGGGCCATAAACTGGAATATAACTATAACAGCCATAACGTGTATATTACCGCGGATAAACAGAAAAACGGCATTAAAGCGAACTTTAAAATTCGCCATAACAT
TGAAGATGGCGGCGTGCAGCTGGCGGATCATTATCAGCAGAACACCCCGATTGGCGATGGCCCGGTGCTGCTGCCGGATAACCATTATCTGAGCTATCAGAGC
GCGCTGAGCAAAGATCCGAACGAAAAACGCGATCATATGGTGCTGCTGGAATTTGTGACCGCGGCGGGCATTACCCTGGGCATGGATGAACTGTATAAAGGCA
GCTGActagagtccgcaaaaatcaccagtctctctctacaaatctatctctctctattttctccagaataatgtgtgagtagttcccagataagggaattag
ggttcttatagggtttcgctcatgtgttgagcatataagaaacccttagtatgtatttgtatttgtaaaatacttctatcaataaaatttctaattcctaaaa
ccaaaatccagtgacccCAGCTGGCGTAATAGCGAAGAGGATGAGCCCAGAACGACGCCCGGCCGACATCCGCCGTGCCACCGAGGCGGACATGCCGGCGGTCT
GCACCATCGTCAACCACTACATCGAGACAAGCACGGTCAACTTCCGTACCGAGCCGCAGGAACCGCAGGAGTGGACGGACGACCTCGTCCGTCTGCGGGAGCG
CTATCCCTGGCTCGTCGCCGAGGTGGACGGCGAGGTCGCCGGCATAGCCTACGCGGGTCCCTGGAAGGCACGCAACGCCTACGACTGGACGGCCGAGTCGACC
GTGTACGTGTCCCCCCGCCACCAGCGGACGGGACTGGGCTCCACGCTCTACACCCACCTGCTGAAGTCCCTGGAGGCACAGGGCTTCAAGAGCGTGGTCGCTG
TCATCGGGCTGCCCAACGACCCGAGCGTGCGCATGCACGAGGCGCTCGGATATGCCCCCCGCGGCATGCTGCGGGCGGCCGGCTTCAAGCACGGGAACTGGCA
TGACGTGGGTTTCTGGCAGCTGGACTTCAGCCTGCCGGTGCCGCCCCGTCCGGTCCTGCCCGTCACCGAAATCTGAAATCATAAAAACCCATCTCATAAATAA
CGTCATGCATTACATGTTAATTATTACATGCTTAACGTAATTCAACAGAAATTATATGATAATCATCGCAAGACCGGCAACAGGATTCAATCTTAAGAAACTT
TATTGCCAAATGTTTGAACGATCGGGGAAATTCGAGCTGGATCCCCGGGTACCGAGCTCGAATTCGTAATCATGGTCATAGCTGTTTCCTGTGTGAAATTGTT
ATCCGCTCACAATTCCACACAACATACGAGCCGGAAGCATAAAGTGTAAAGCCTGGGGTGCCTAATGAGTGAGCTAACTCACATTAATTGCGTTGCGCTCACT
GCCCGCTTTCCAGTCGGGAAACCTGTCGTGCCAGGATCAGATTGTCGTTTCCCGCCTTCAGTTTAAACTATCAGTGTTTGACAGGATATATTGGCGGGTAAAC
CTAAGAGAAAAGAGCGTTTATTAGAATAATCGGATATTTAAAAGGGCGTGAAAAGGTTTATCCGTTCGTCCATTTGTATGTGACTAAAACAATTCATCCAGTA
AAATATAATATTTTATTTTCTCCCAATCAGGCTTGATCCCCAGTAAGTCAAAAAATAGCTCGACATACTGTTCTTCCCCGATATCCTCCCTGATCGACCGGAC
GCAGAAGGCAATGTCATACCACTTGTCCGCCCTGCCGCTTCTCCCAAGATCAATAAAGCCACTTACTTTGCCATCTTTCACAAAGATGTTGCTGTCTCCCAGG
TCGCCGTGGGAAAAGACAAGTTCCTCTTCGGGCTTTTCCGTCTTTAAAAAATCATACAGCTCGCGCGGATCTTTAAATGGAGTGTCTTCTTCCCAGTTTTCGC
AATCCACATCGGCCAGATCGTTATTCAGTAAGTAATCCAATTCGGCTAAGCGGCTGTCTAAGCTATTCGTATAGGGACAATCCGATATGTCGATGGAGTGAAA
GAGCCTGATGCACTCCGCATACAGCTCGATAATCTTTTCAGGGCTTTGTTCATCTTCATACTCTTCCGAGCAAAGGACGCCATCGGCCTCACTCATGAGCAGA
TTGCTCCAGCCATCATGCCGTTCAAAGTGCAGGACCTTTGGAACAGGCAGCTTTCCTTCCAGCCATAGCATCATGTCCTTTTCCCGTTCCACATCATAGGTGG
TCCCTTTATACCGGCTGTCCGTCATTTTTAAATATAGGTTTTCATTTTCTCCCACCAGCTTATATACCTTAGCAGGAGACATTCCTTCCGTATCTTTTACGCA
GCGGTATTTTTCGATCAGTTTTTTCAATTCCGGTGATATTCTCATTTTAGCCATTTATTATTTCCTTCCTCTTTTCTACAGTATTTAAAGATACCCCAAGAAG
CTAATTATAACAAGACGAACTCCAATTCACTGTTCCTTGCATTCTAAAACCTTAAATACCAGAAAACAGCTTTTTCAAAGTTGTTTTCAAAGTTGGCGTATAA
CATAGTATCGACGGAGCCGATTTTGAAACCACAATTATGGGTGATGCTGCCAACTTACTGATTTAGTGTATGATGGTGTTTTTGAGGTGCTCCAGTGGCTTCT
GTTTCTATCAGCTGTCCCTCCTGTTCAGCTACTGACGGGGTGGTGCGTAACGGCAAAAGCACCGCCGGACATCAGCGCTATCTCTGCTCTCACTGCCGTAAAA
CATGGCAAC
