# Supplementary figures and images for: Heritable transgene-free genome editing in plants by grafting of wild-type shoots to transgenic donor rootstocks
Source: Nat Biotechnol. 2023 Jan 2;41(7):958–67. doi: 10.1038/s41587-022-01585-8 (PMC10344777; doi:10.1038/s41587-022-01585-8)

# Source Data Fig. 2b

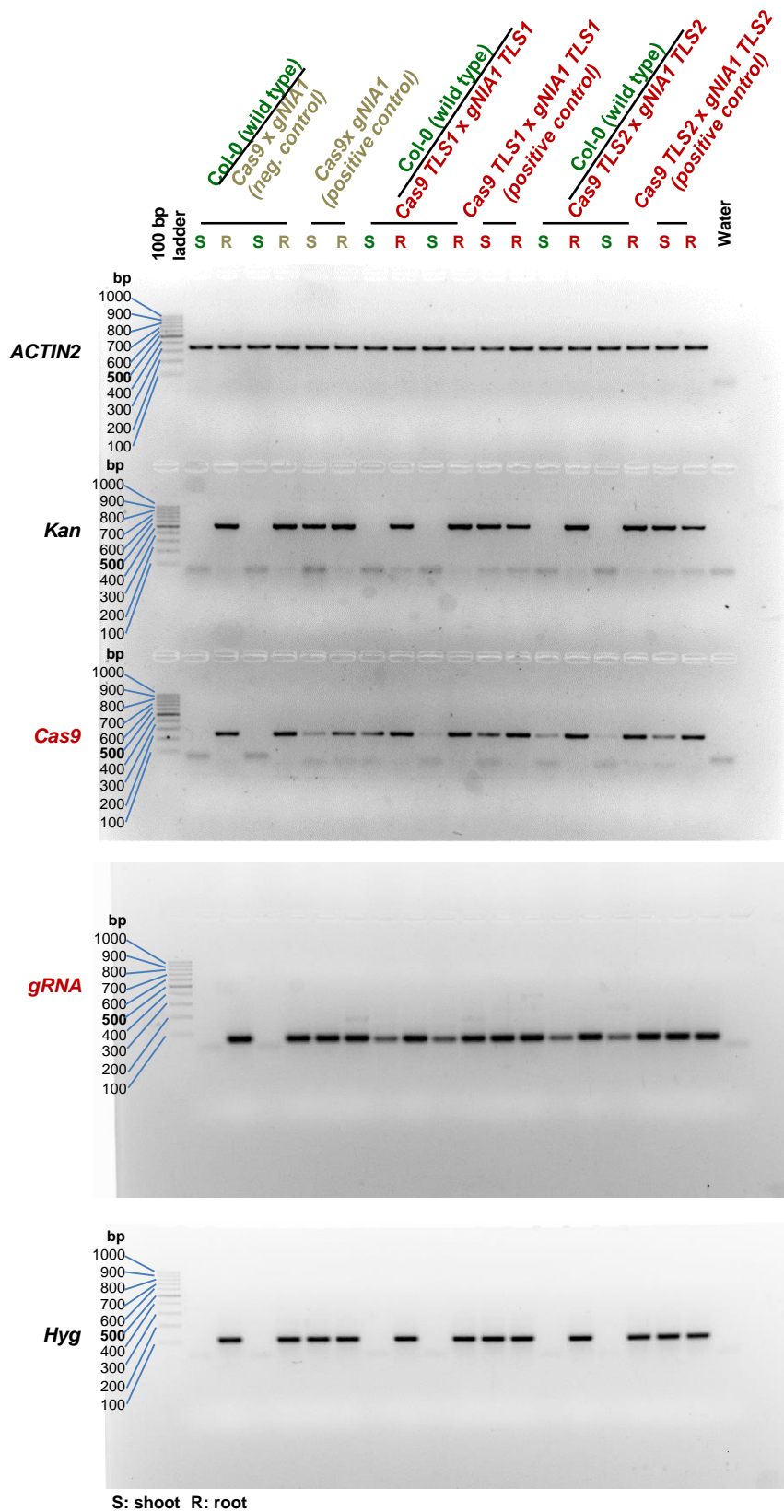

# Source Data Fig. 2d

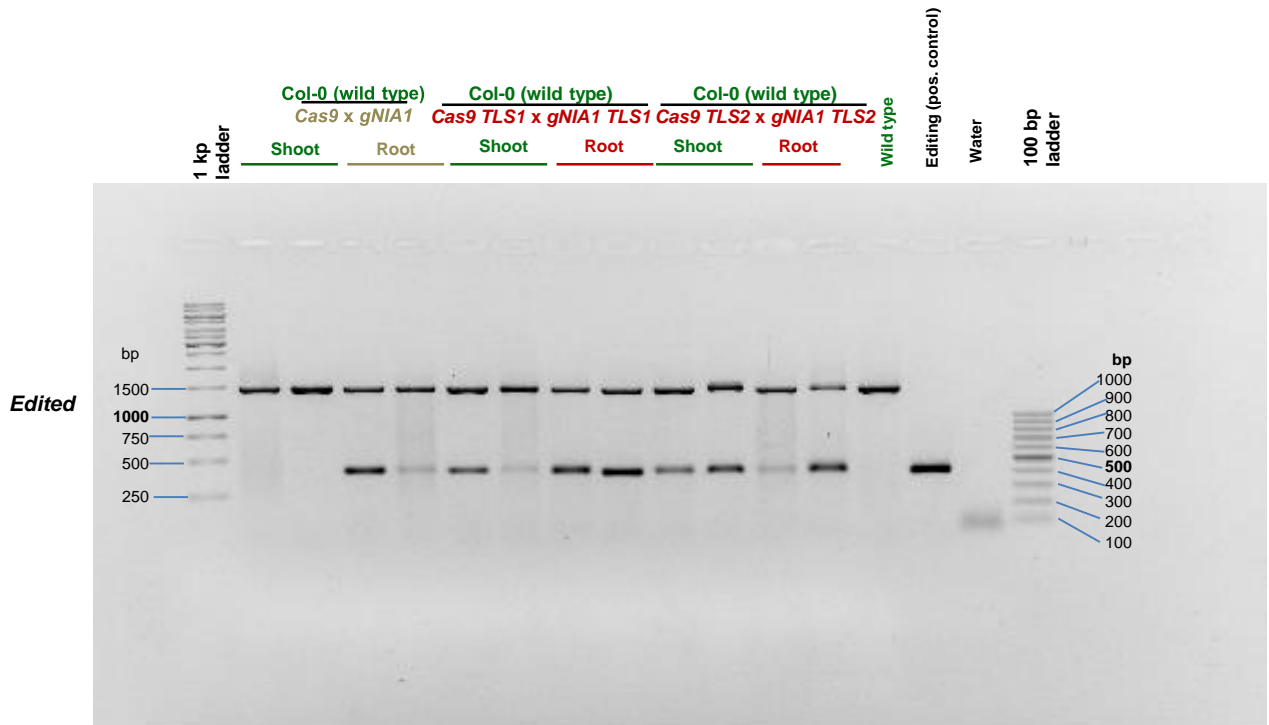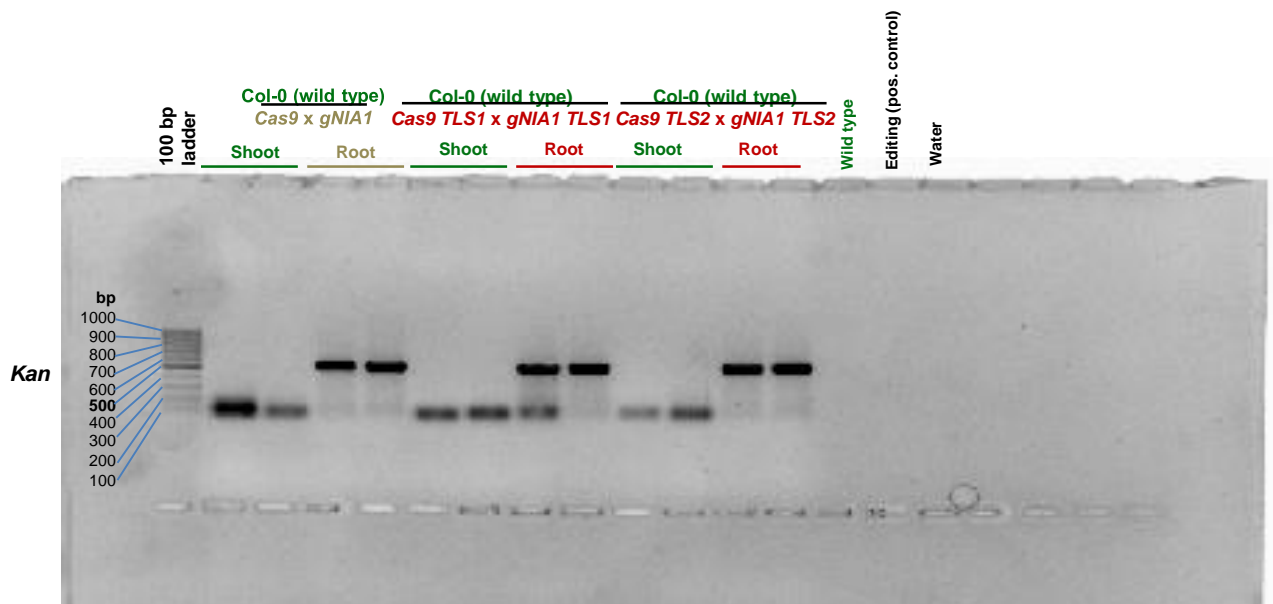

Supplement: Source Data Fig. 2 — Unprocessed gels. [file 41587_2022_1585_MOESM3_ESM.pdf]

# Source Data Fig. 3b

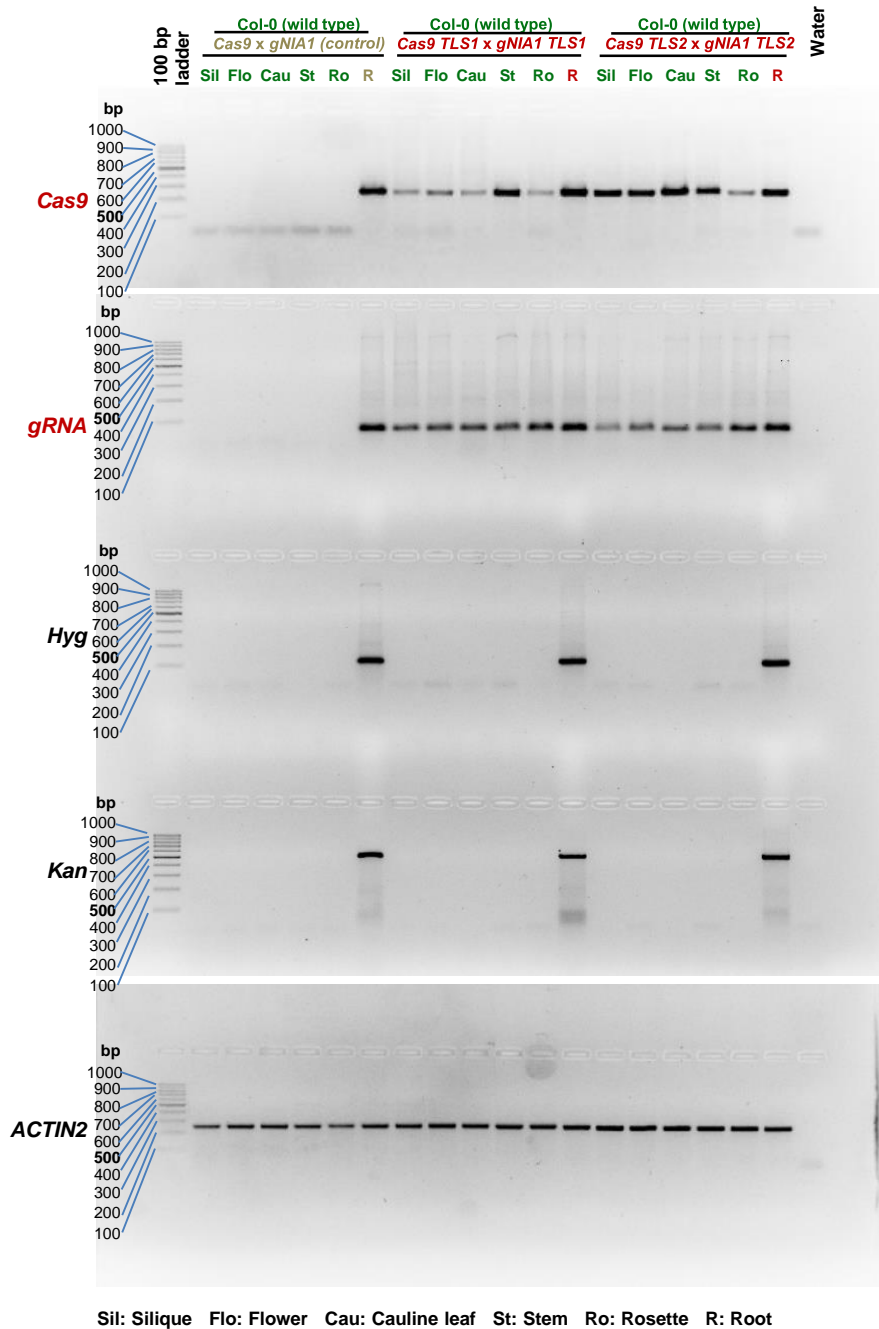

# Source Data Fig. 3d

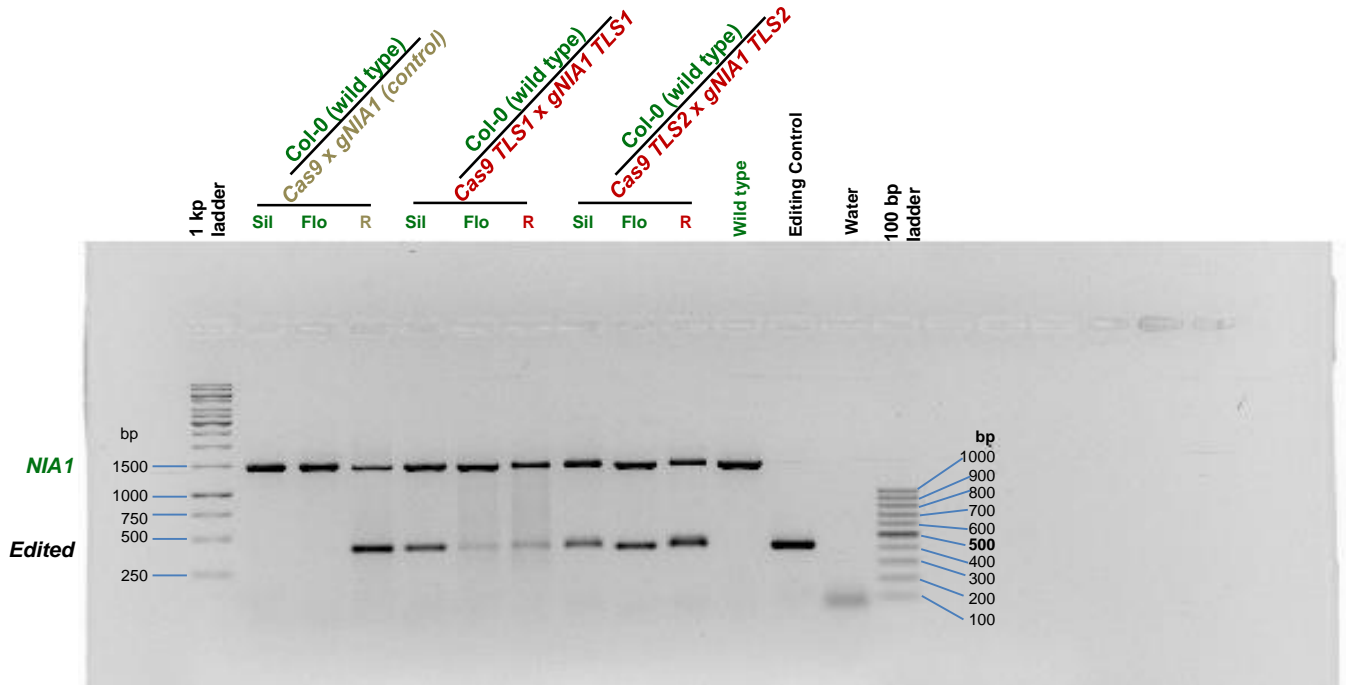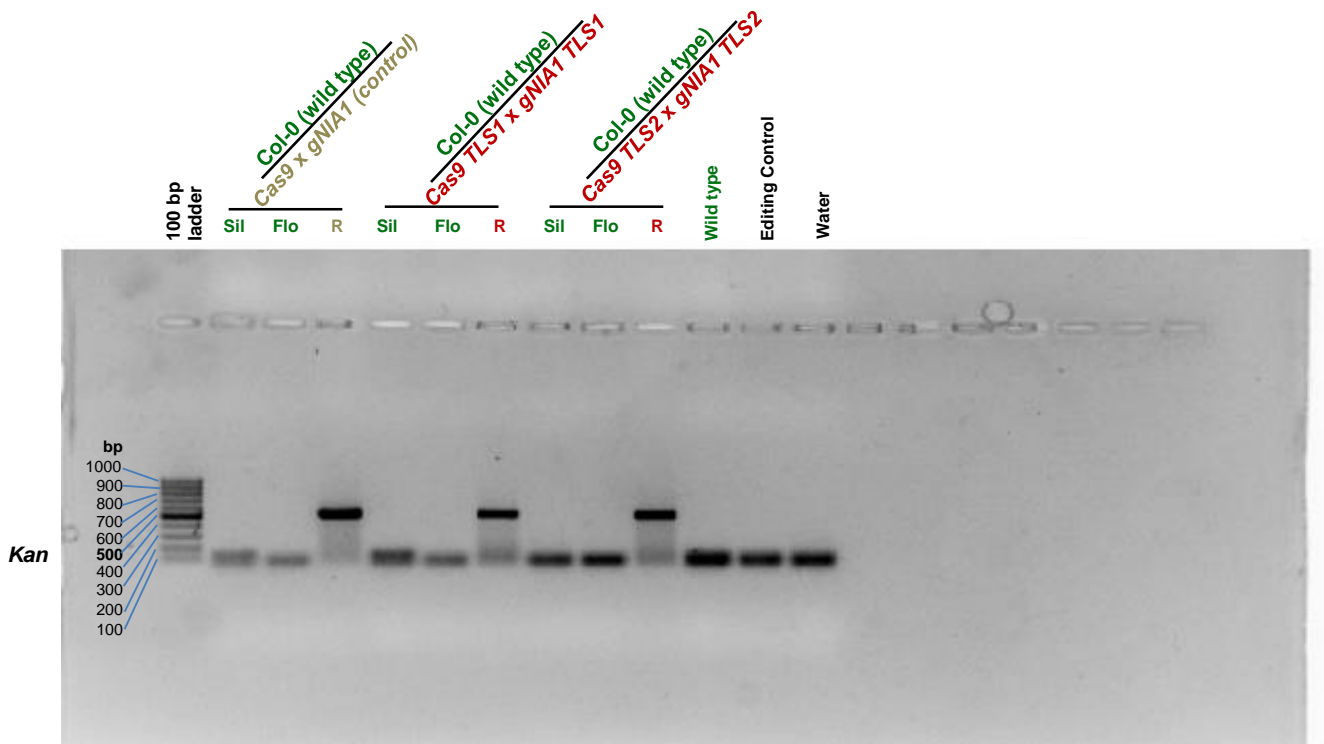

Sil: Silique Flo: Flower R: Root

Supplement: Source Data Fig. 3 — Unprocessed gels. [file 41587_2022_1585_MOESM4_ESM.pdf]

## Source Data Fig. 4a

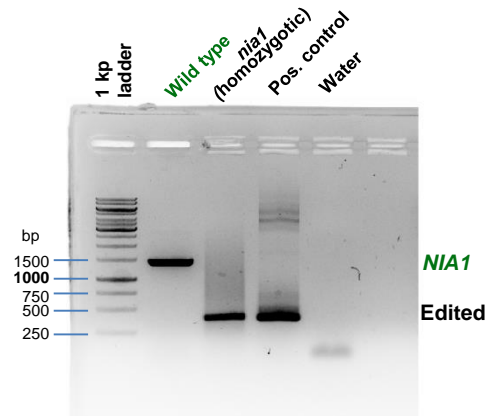

# Source Data Fig. 4b

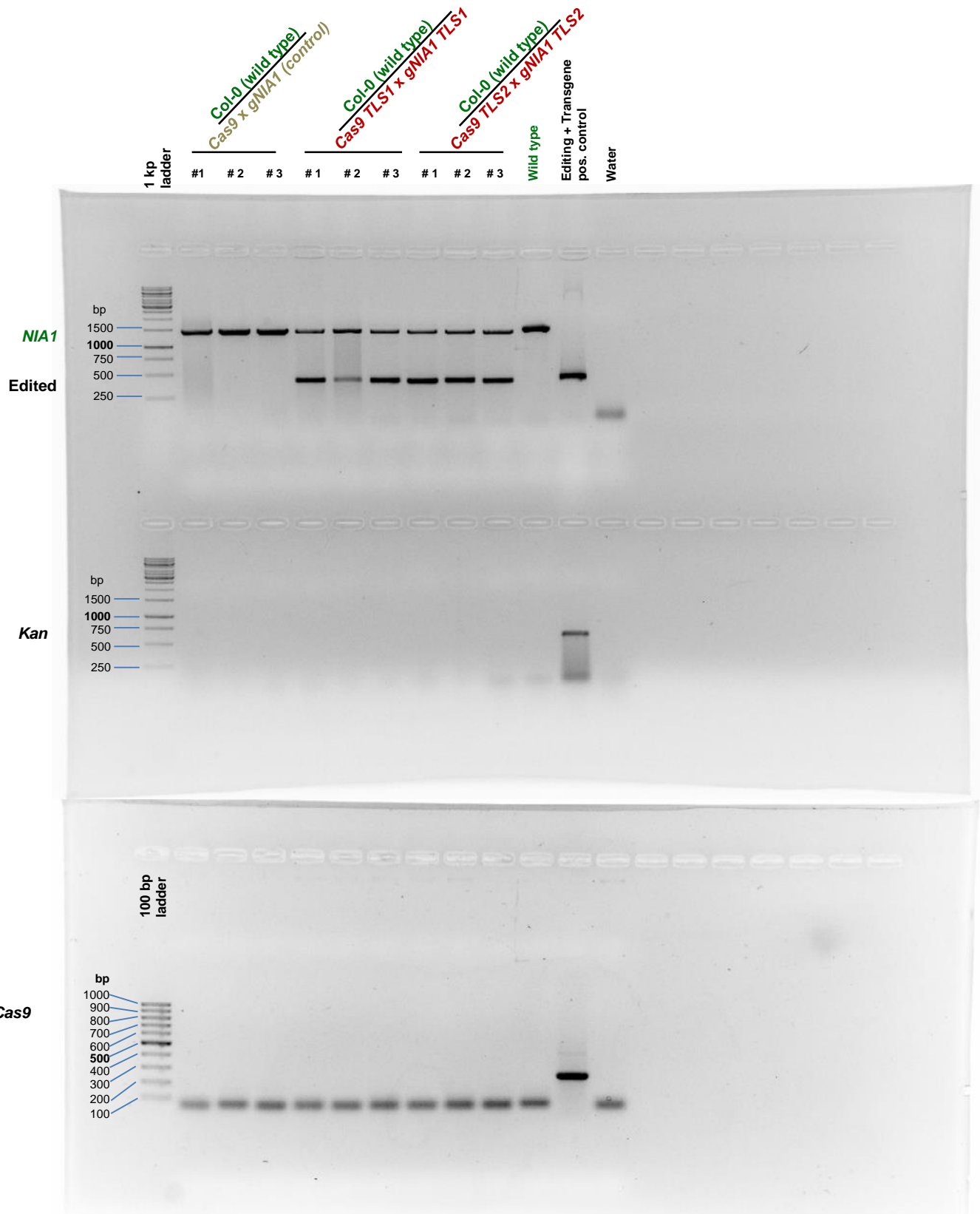

Supplement: Source Data Fig. 4 — Unprocessed gels. [file 41587_2022_1585_MOESM5_ESM.pdf]

# Source Data Fig. 5b

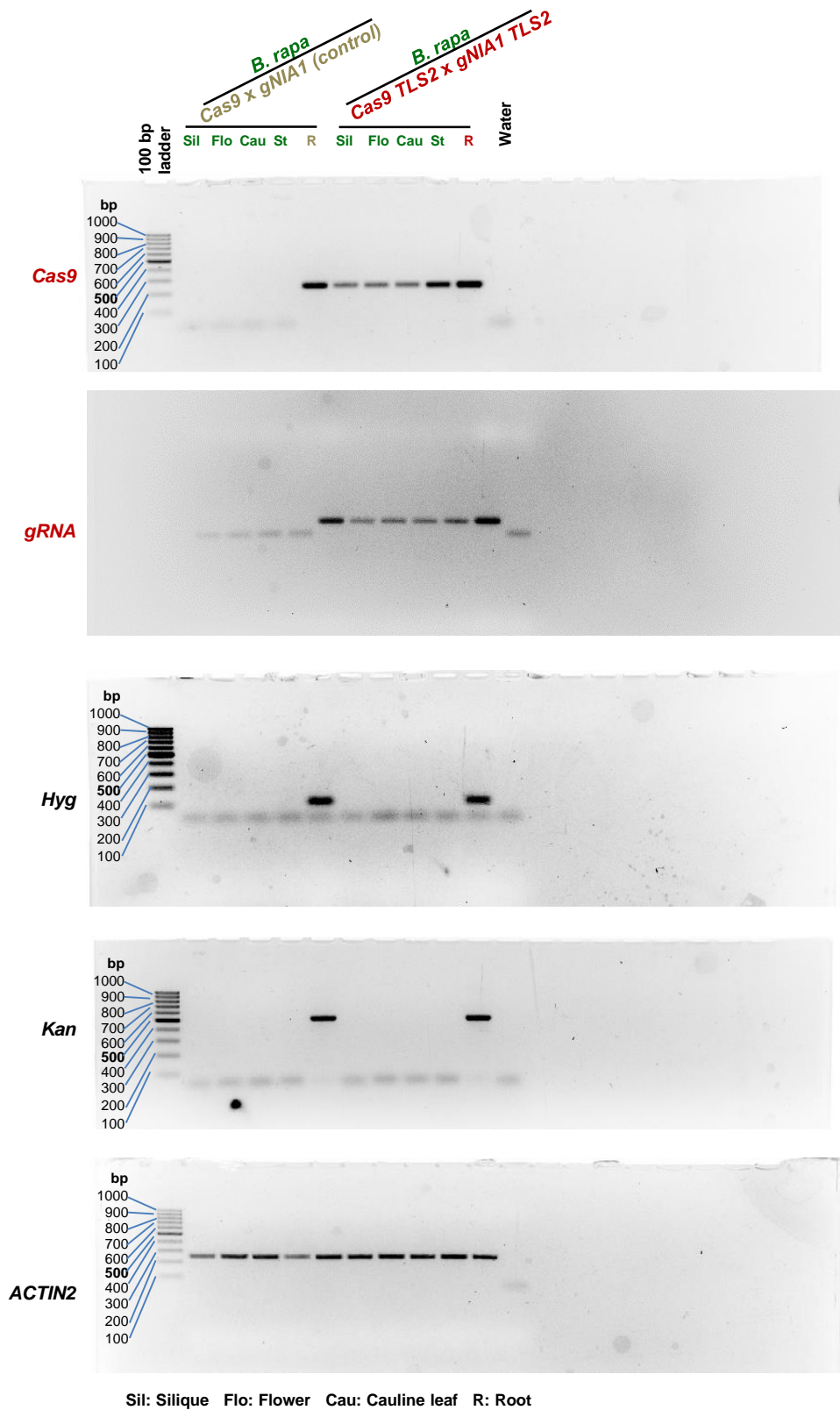

# Source Data Fig. 5d

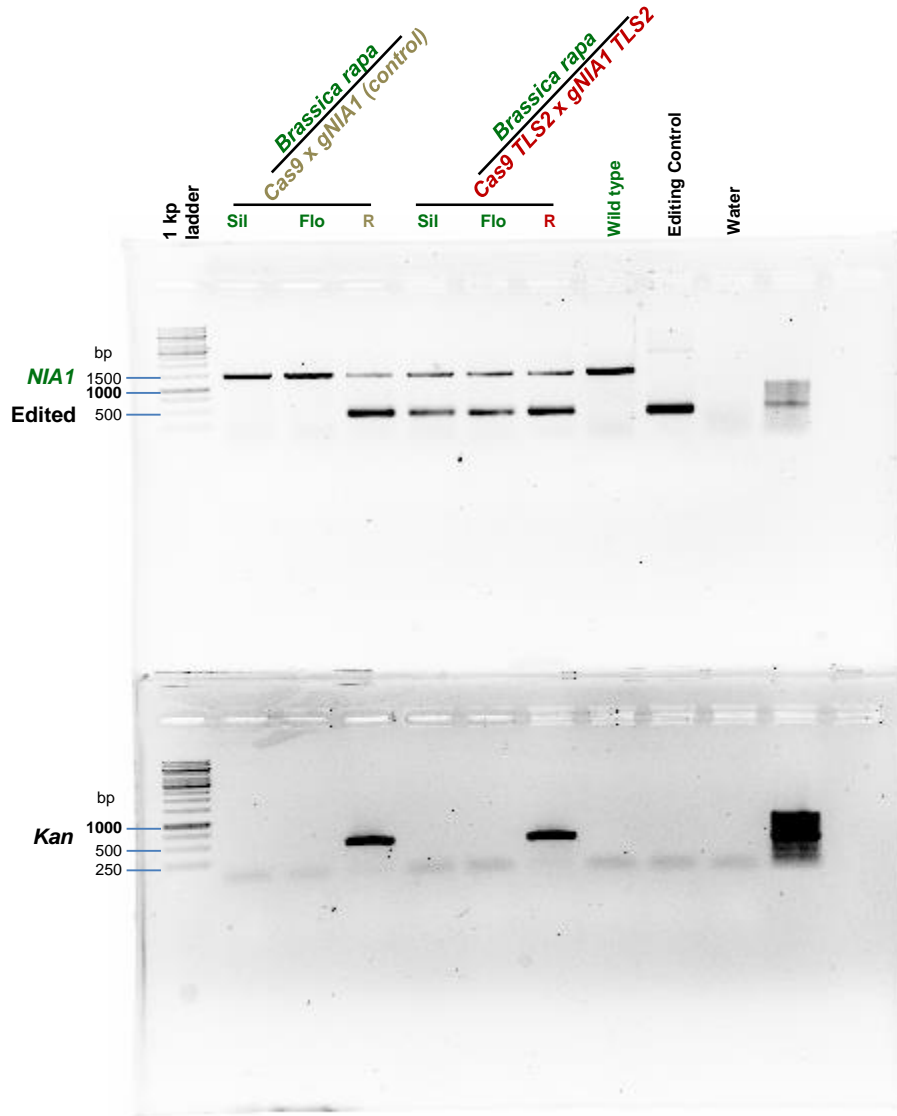

Supplement: Source Data Fig. 5 — Unprocessed gels. [file 41587_2022_1585_MOESM6_ESM.pdf]

# Source Data Extended Fig. 1-1

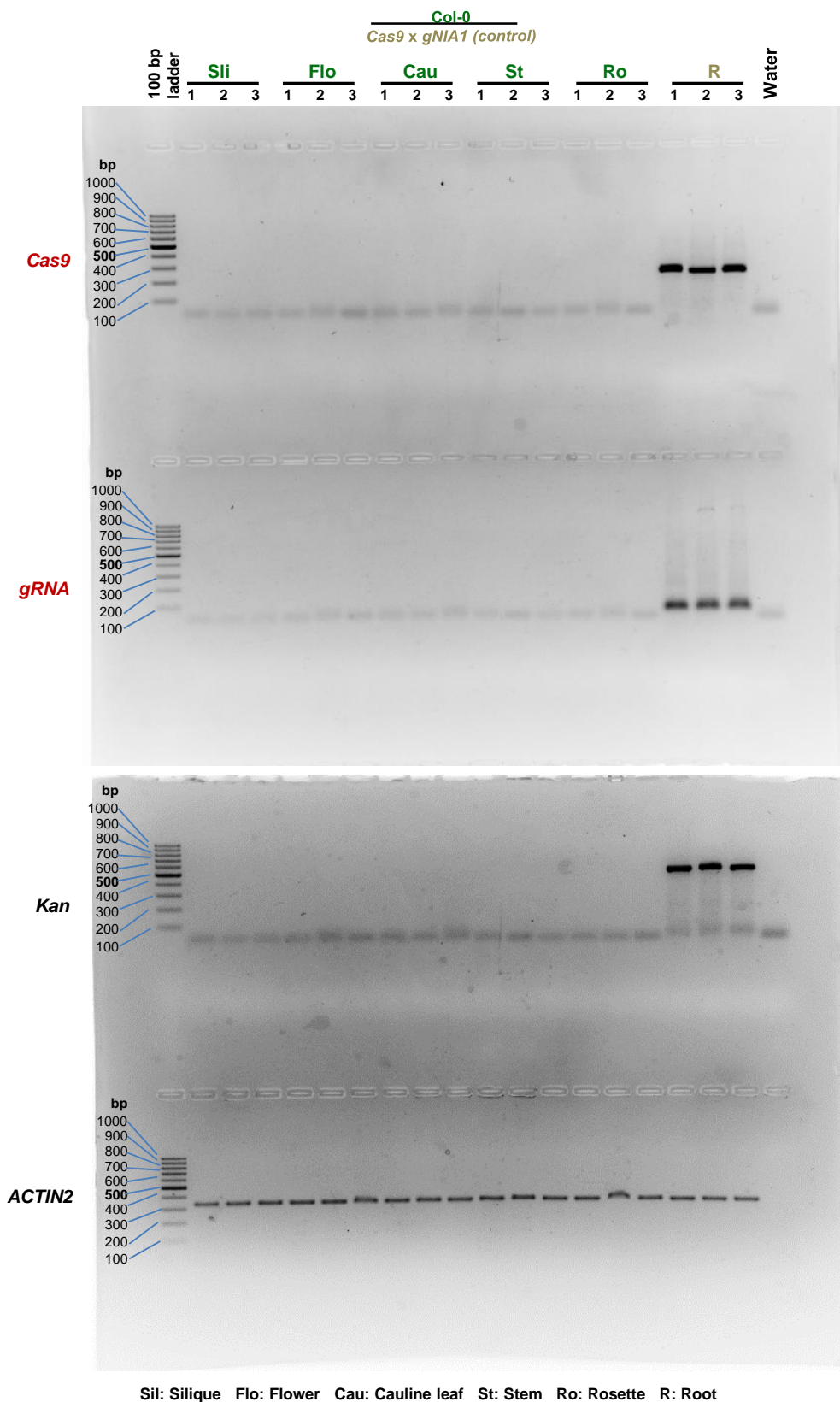

Source Data Extended Fig. 1-2

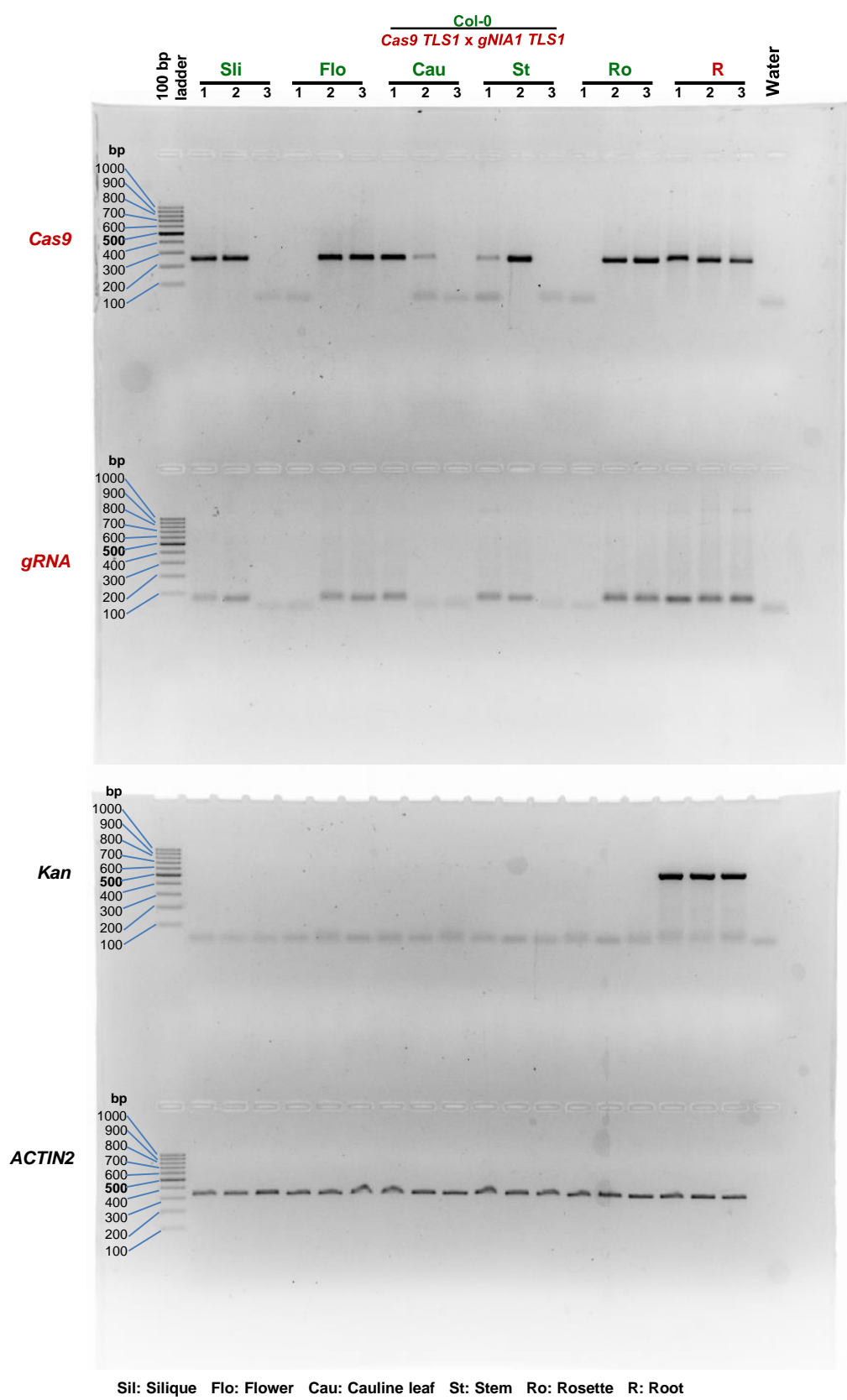

# Source Data Extended Fig. 1-3

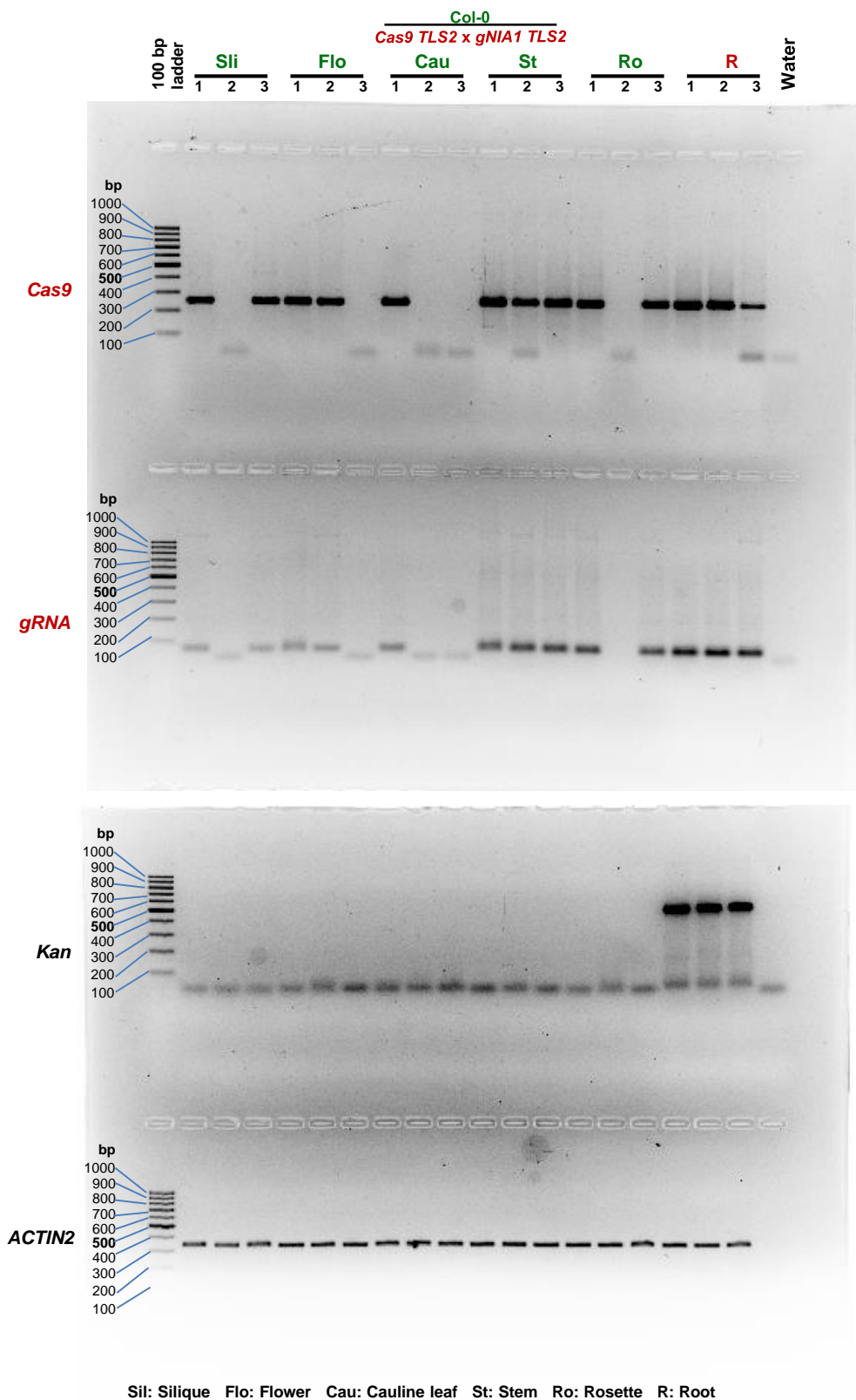

Supplement: Source Data Extended Data Fig. 1 — Unprocessed gels. [file 41587_2022_1585_MOESM7_ESM.pdf]

# Source Data Extended Fig. 2-1

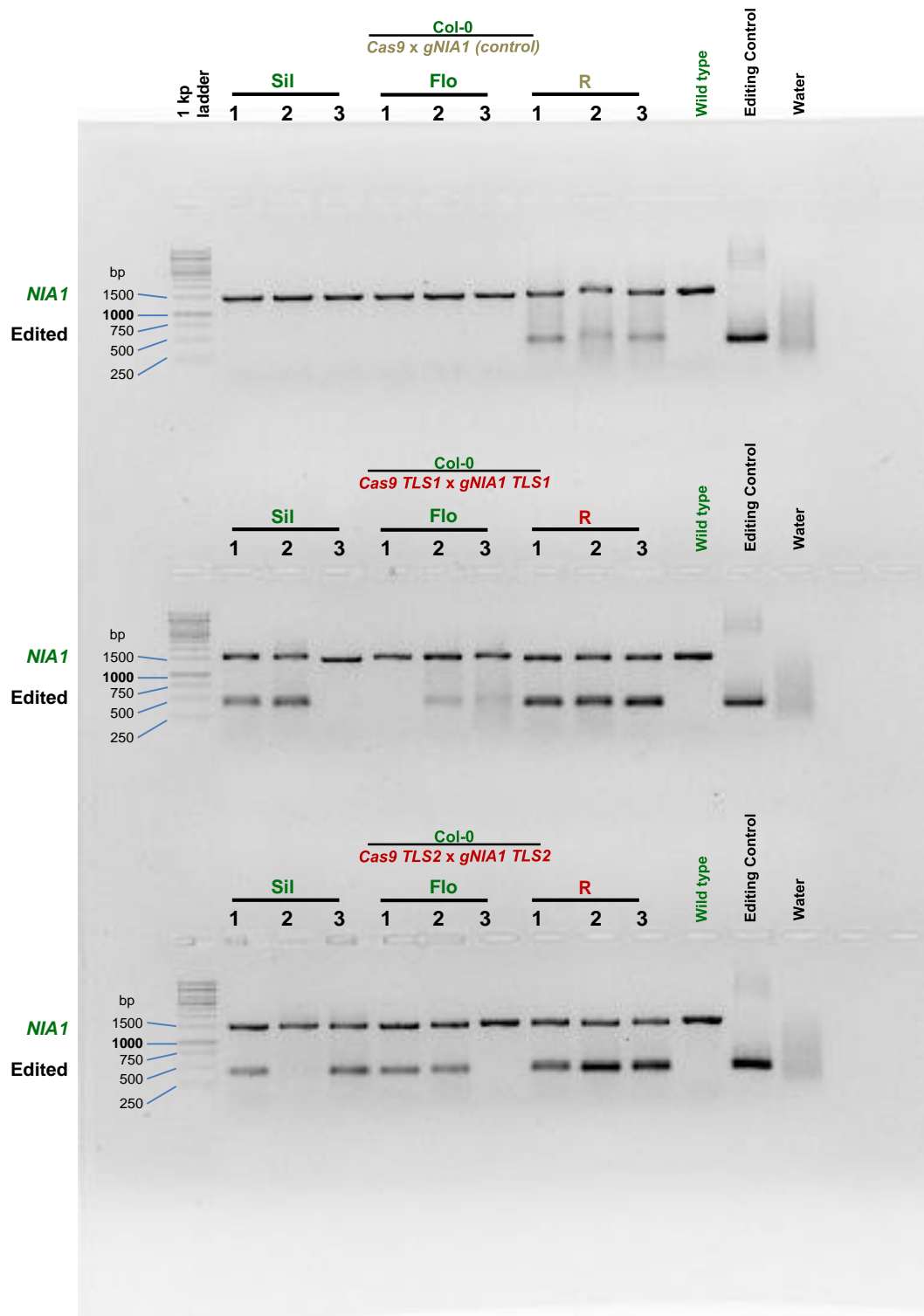

Sil: Silique Flo: Flower R: Root

# Source Data Extended Fig. 2-2

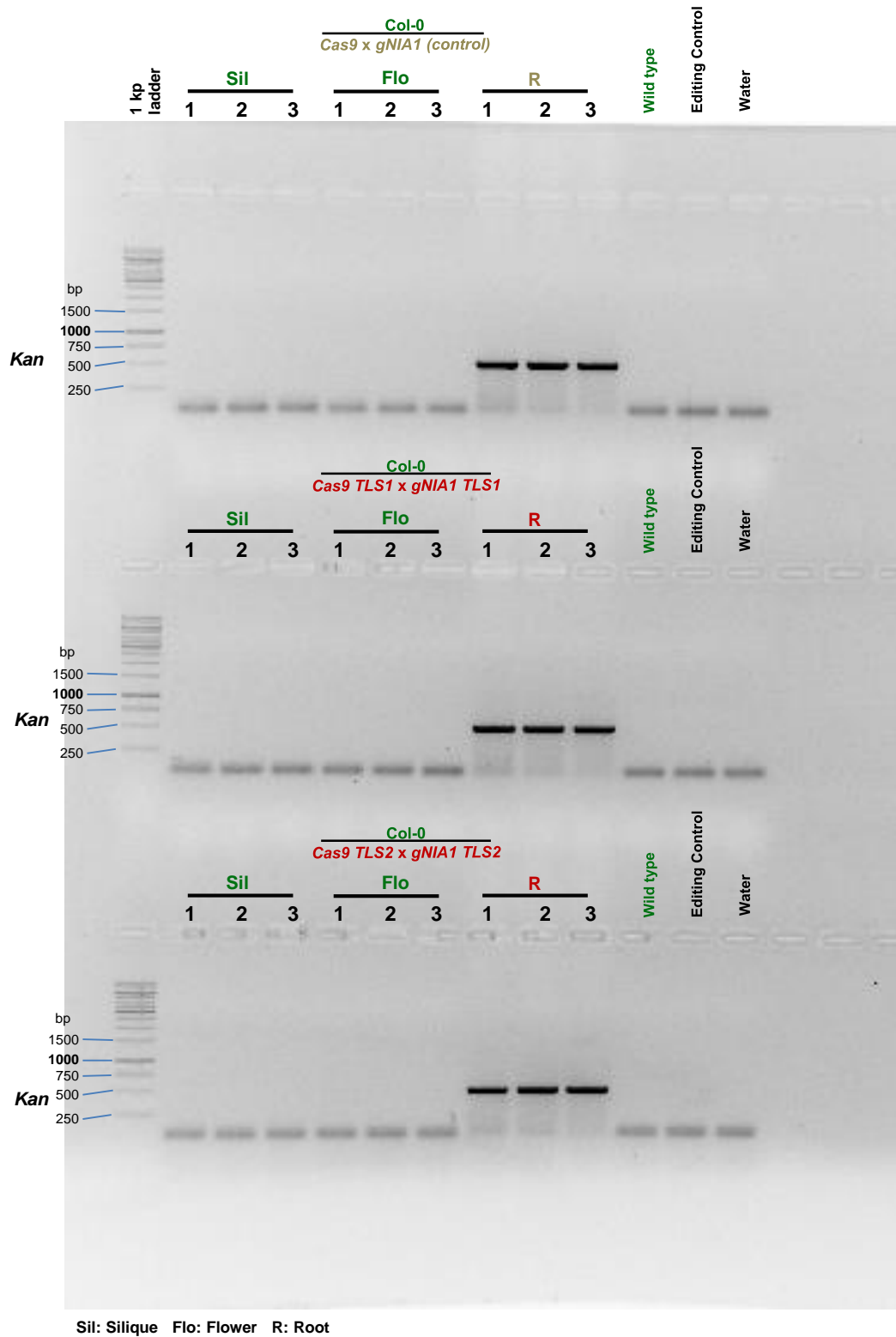

Supplement: Source Data Extended Data Fig. 2 — Unprocessed gels. [file 41587_2022_1585_MOESM8_ESM.pdf]

# Source Data Extended Fig. 3-1

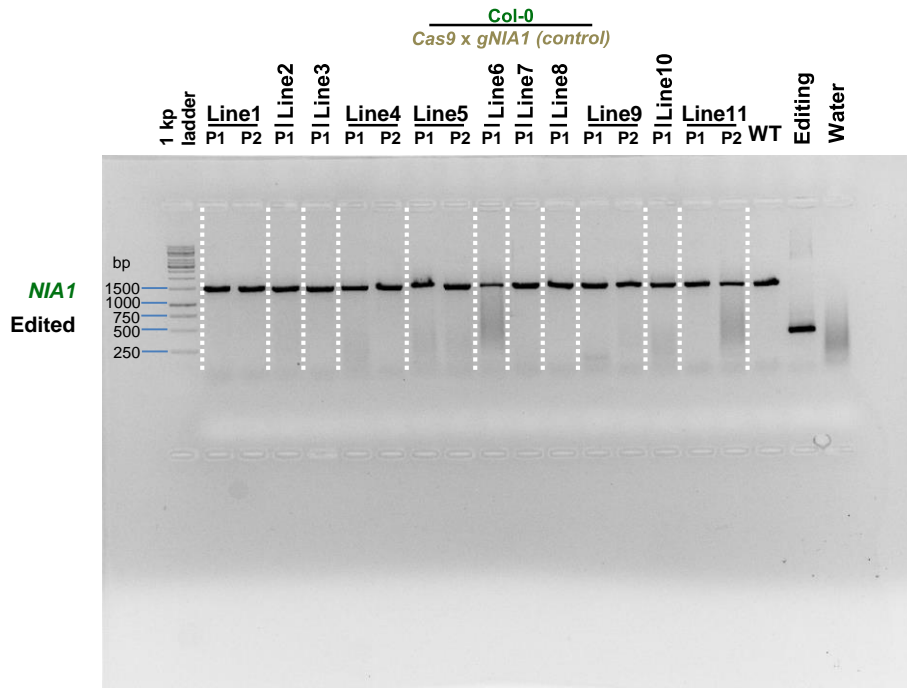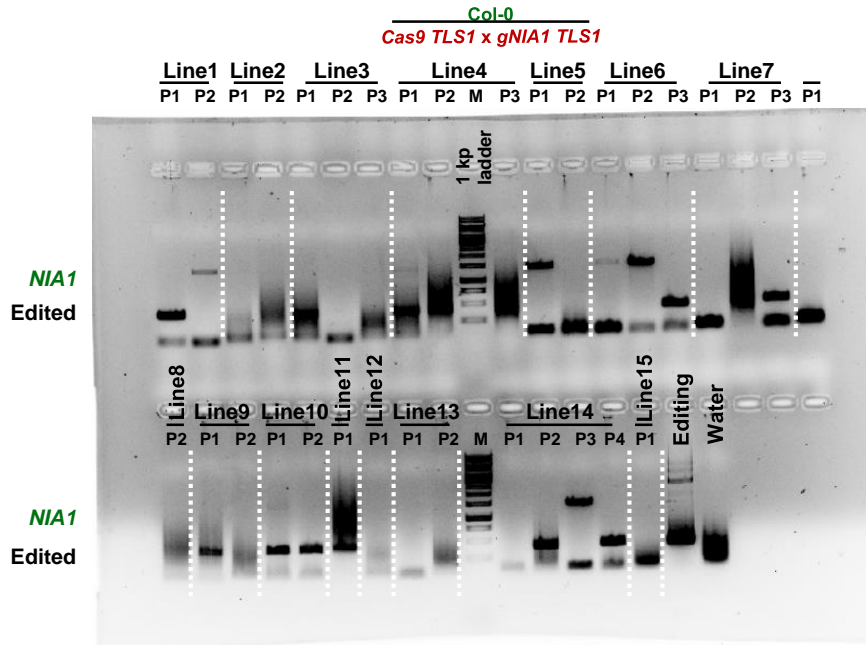

# Source Data Extended Fig. 3-2

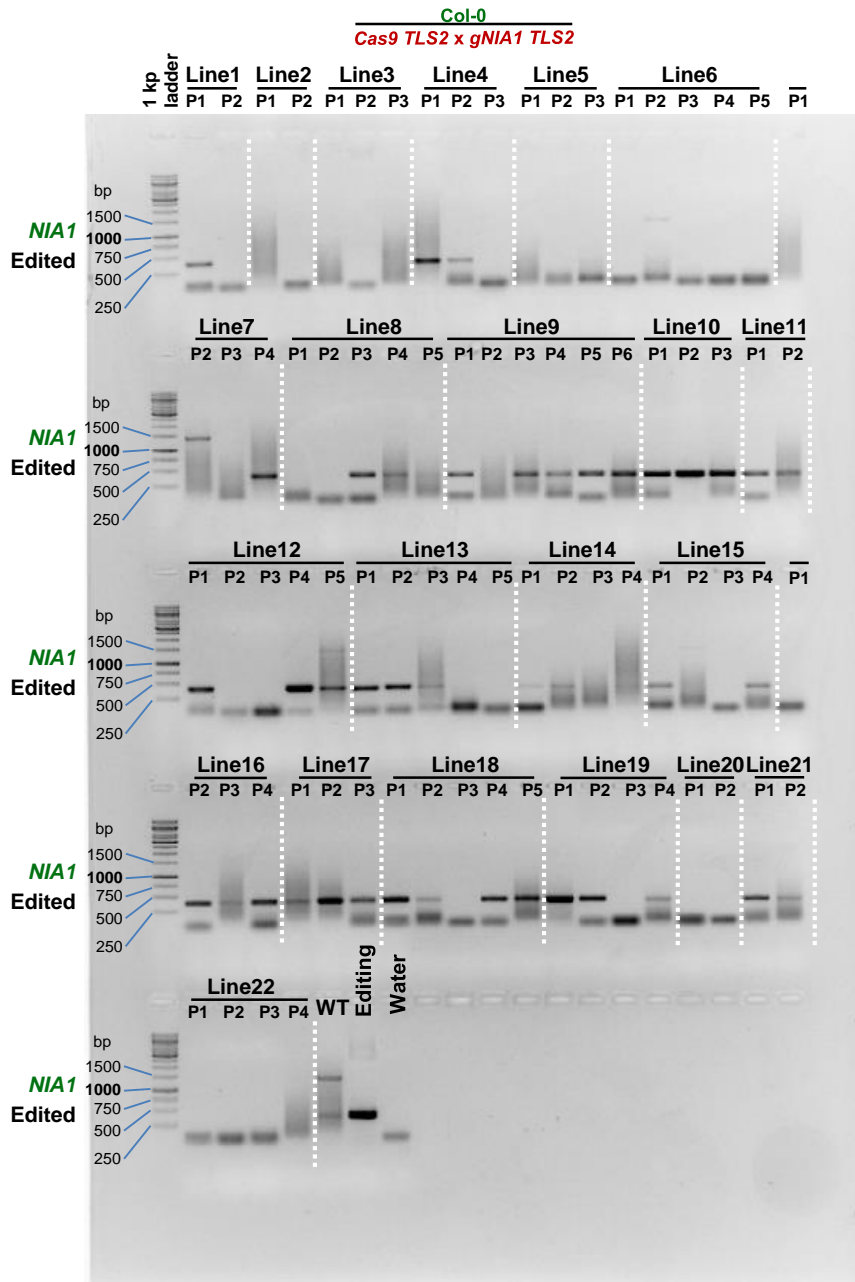

Supplement: Source Data Extended Data Fig. 3 — Unprocessed gels. [file 41587_2022_1585_MOESM9_ESM.pdf]

# Source Data Extended Fig. 4c

*35S<sub>prom</sub>::H2B-Venus*

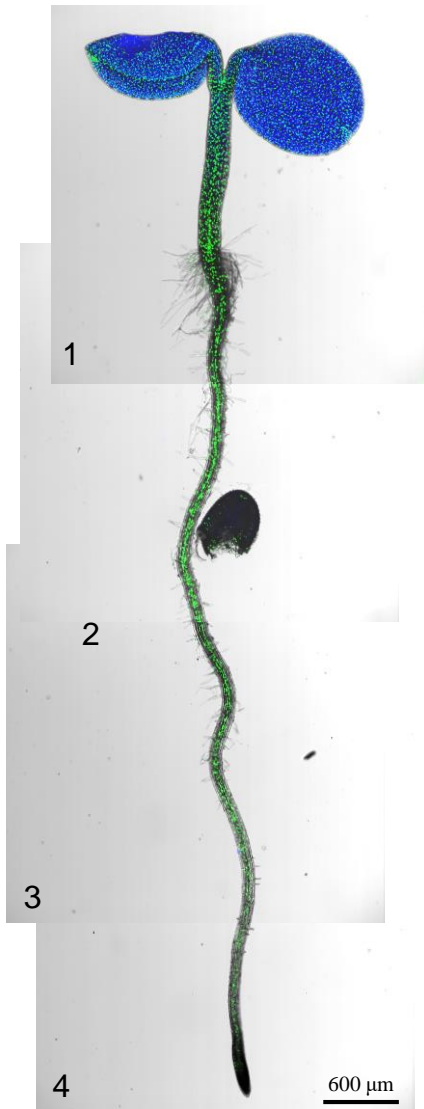

*35S<sub>prom</sub>::H2B-Venus edited*

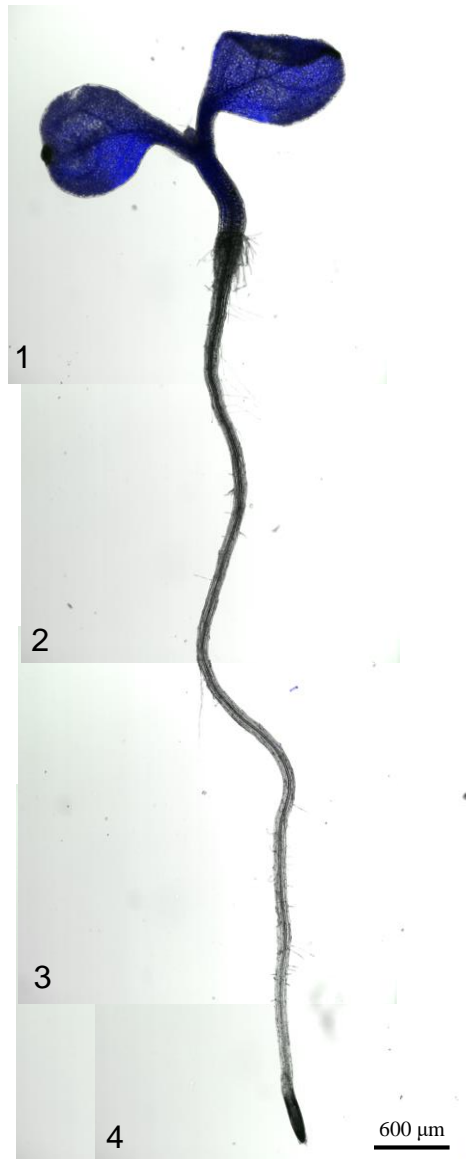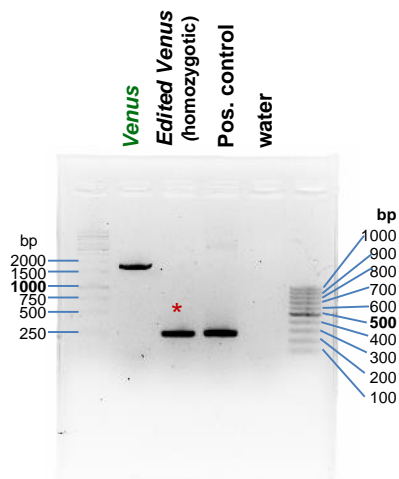

Supplement: Source Data Extended Data Fig. 4 — Unprocessed gels. [file 41587_2022_1585_MOESM10_ESM.pdf]

# Source Data Extended Fig. 7

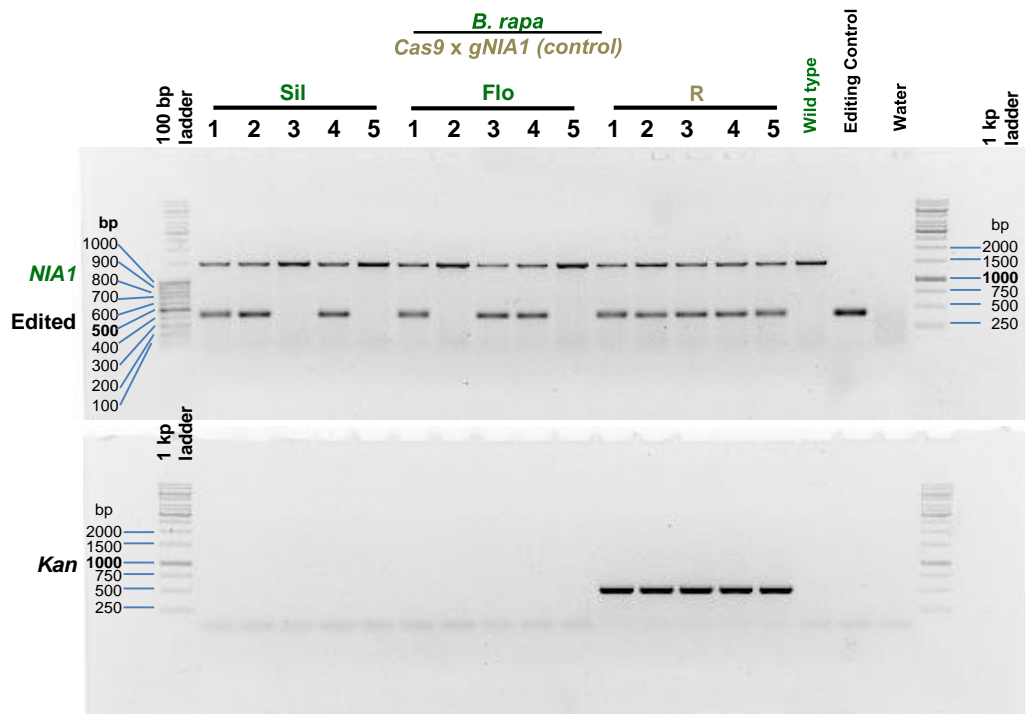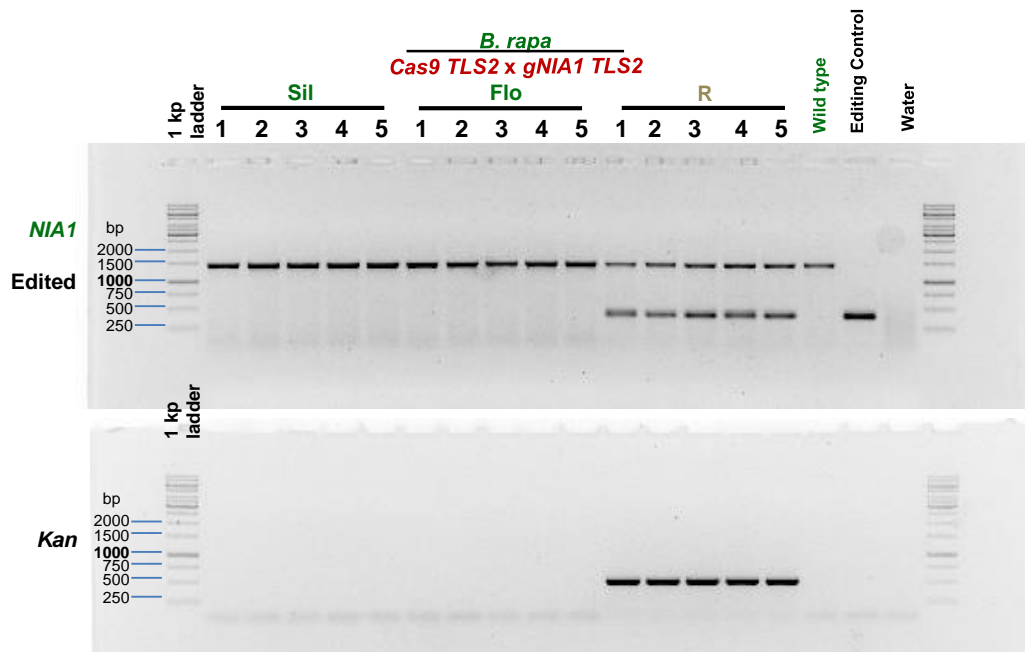

Sil: Silique Flo: Flower R: Root

Supplement: Source Data Extended Data Fig. 7 — Unprocessed gels. [file 41587_2022_1585_MOESM13_ESM.pdf]
