# Supplementary material for: Heritable transgene-free genome editing in plants by grafting of wild-type shoots to transgenic donor rootstocks
Source: Nat Biotechnol. 2023 Jan 2;41(7):958–67. doi: 10.1038/s41587-022-01585-8 (PMC10344777; doi:10.1038/s41587-022-01585-8)

Source Data Extended Fig. 5-1

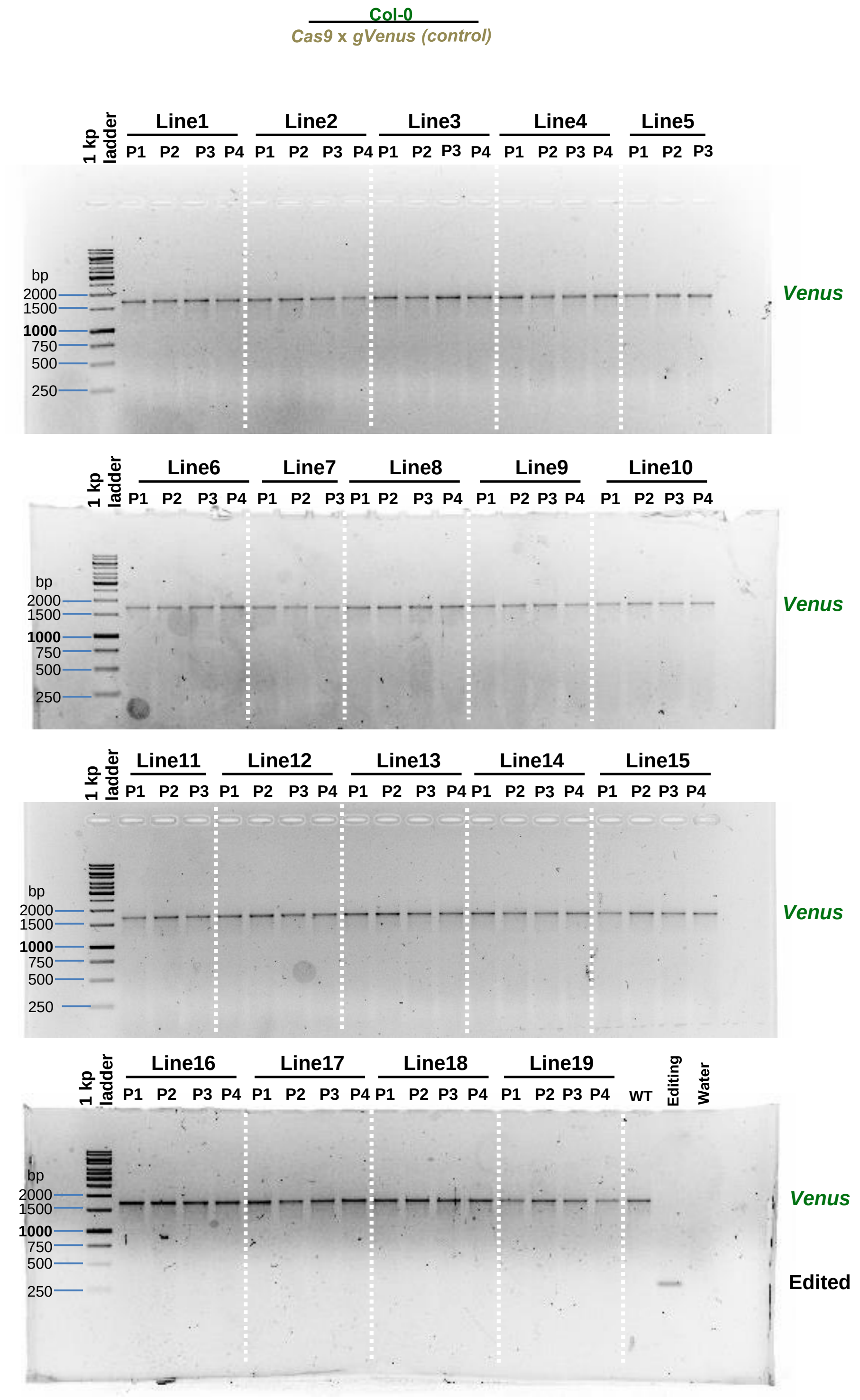

Source Data Extended Fig. 5-2

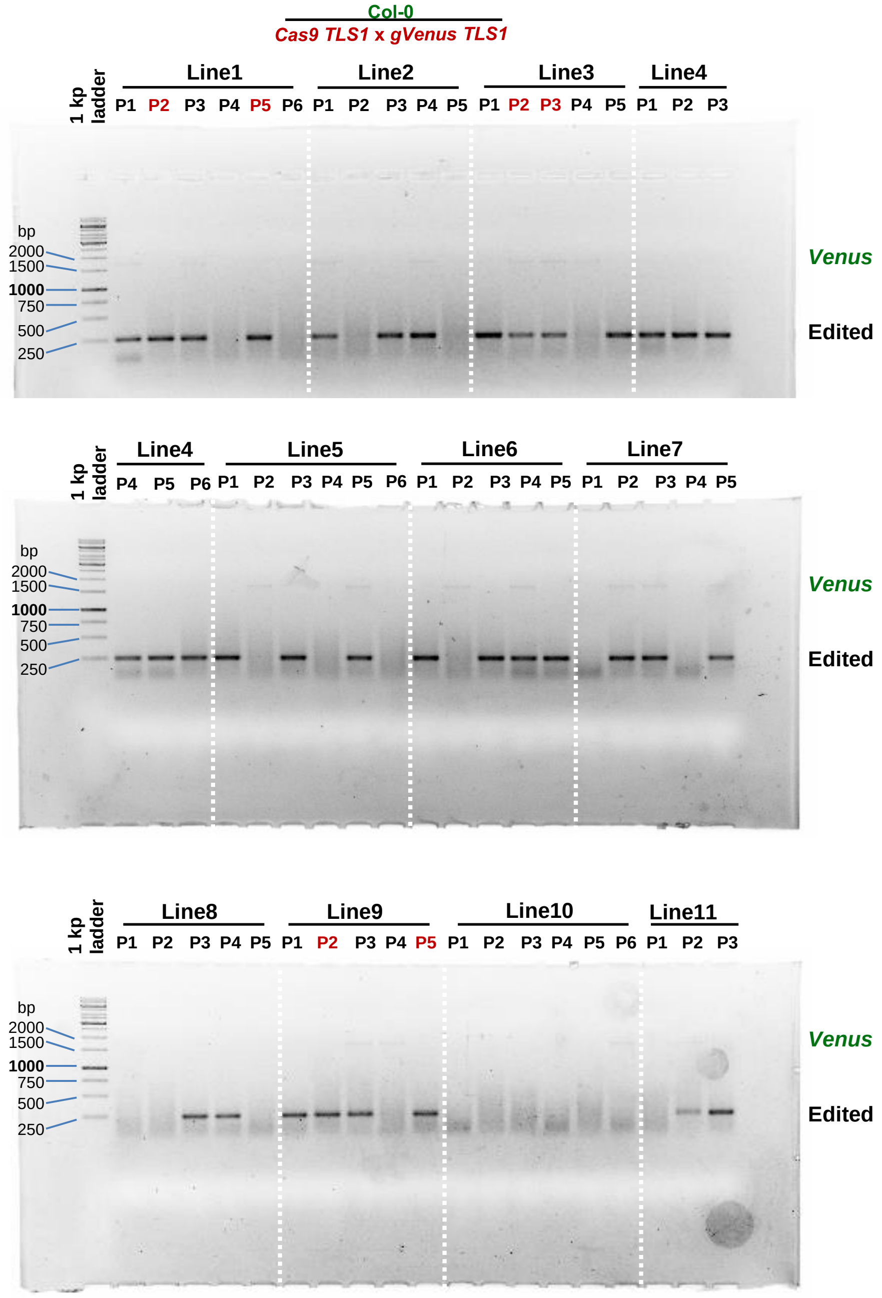

Source Data Extended Fig. 5-2

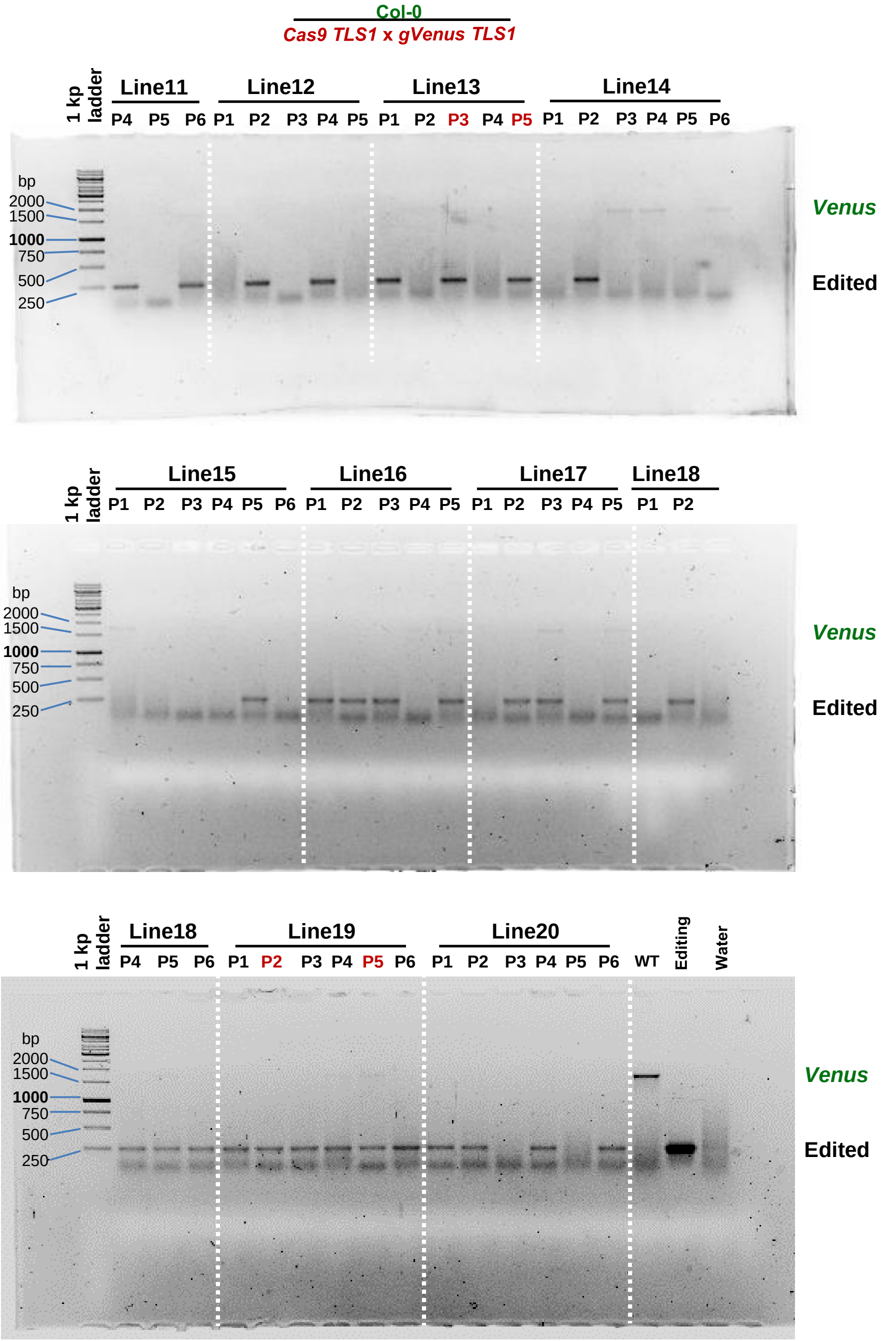

Source Data Extended Fig. 5-3

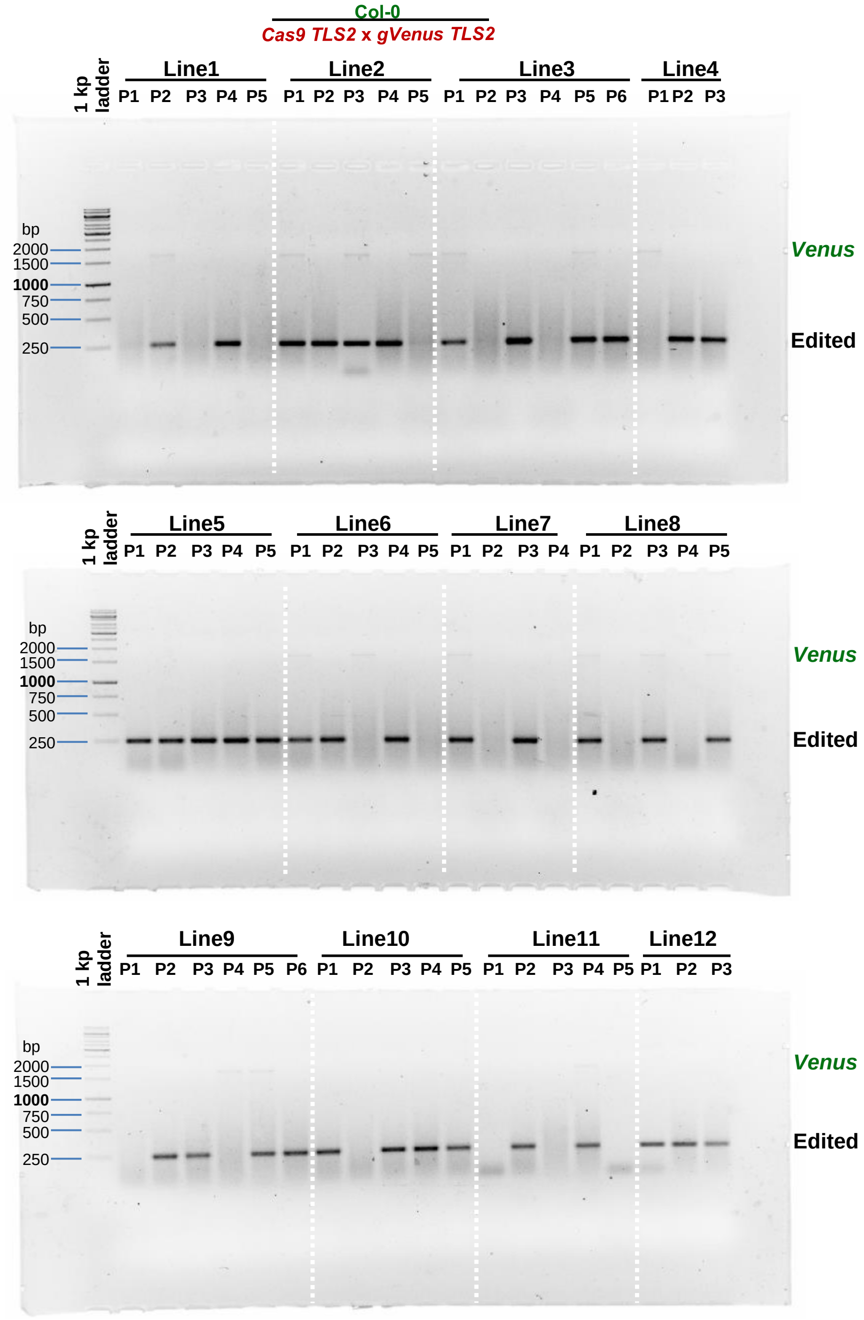

Source Data Extended Fig. 5-3

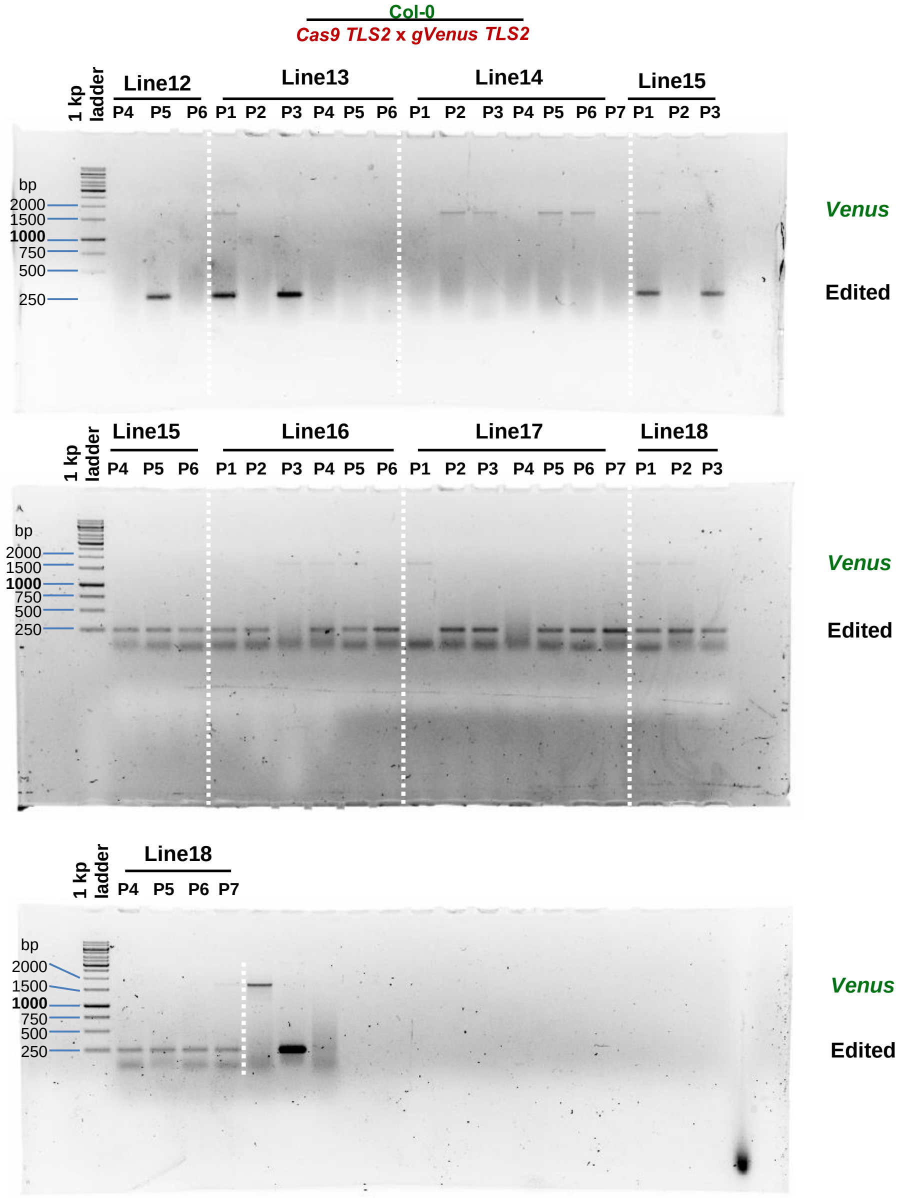

Supplement: Source Data Extended Data Fig. 5 — Unprocessed gels. [file 41587_2022_1585_MOESM11_ESM.pdf]
