# Supplementary material for: Heritable transgene-free genome editing in plants by grafting of wild-type shoots to transgenic donor rootstocks
Source: Nat Biotechnol. 2023 Jan 2;41(7):958–67. doi: 10.1038/s41587-022-01585-8 (PMC10344777; doi:10.1038/s41587-022-01585-8)
Supplement: Source Data Extended Data Fig. 6 — Unprocessed gels. [file 41587_2022_1585_MOESM12_ESM.pdf]

Source Data Extended Fig. 6-1

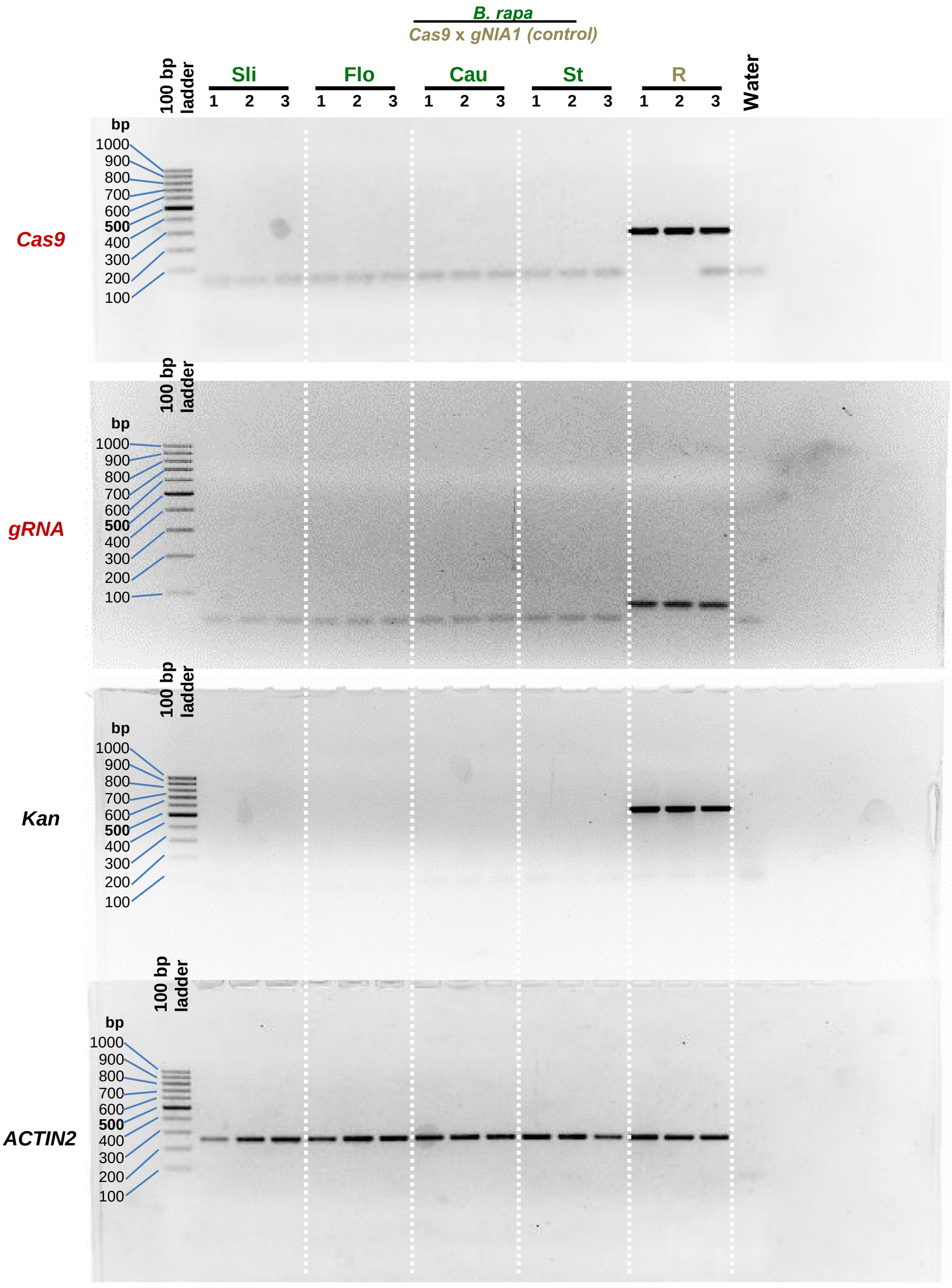

Sil: Silique Flo: Flower Cau: Cauline leaf St: Stem R: Root

Source Data Extended Fig. 6-2

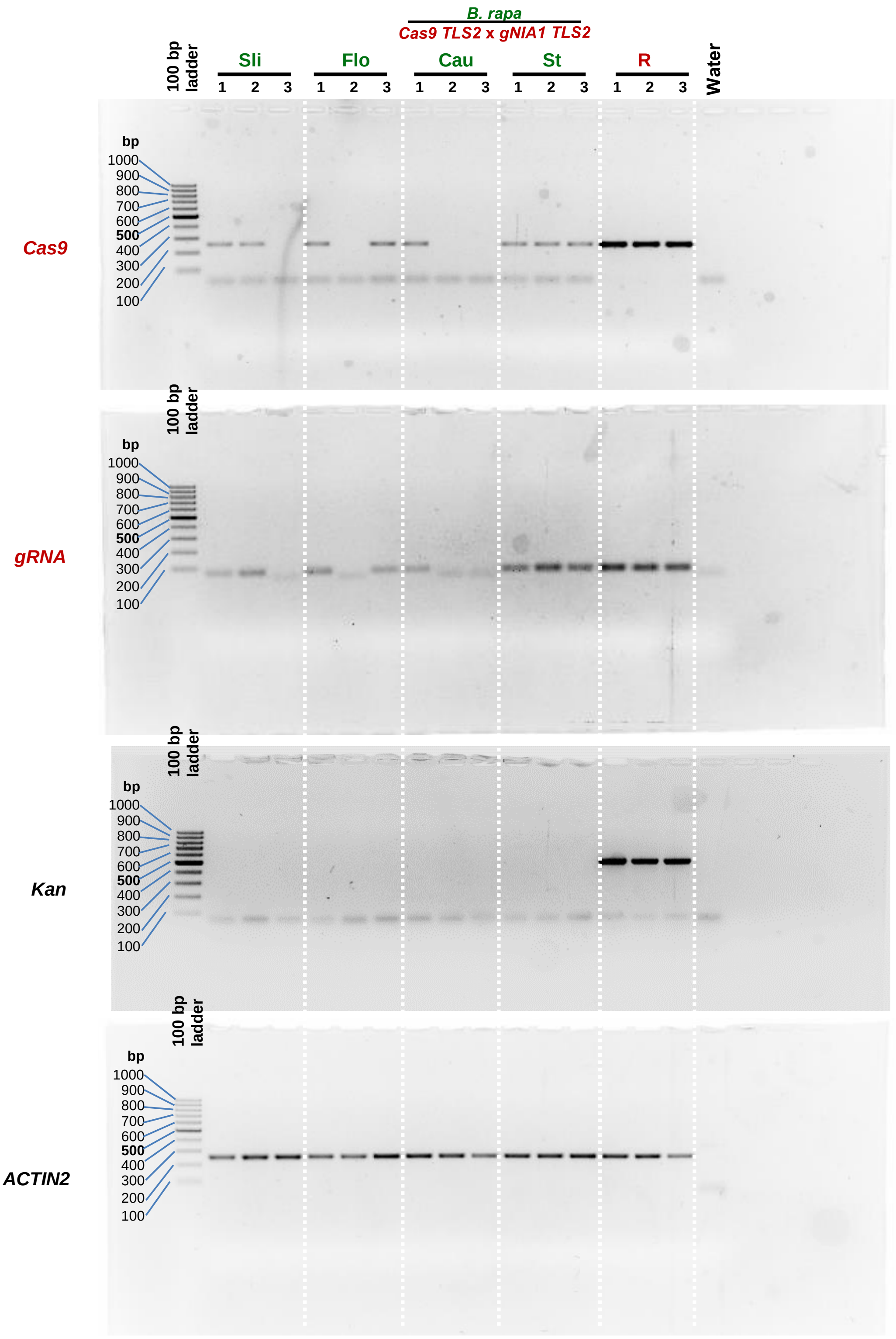

Sil: Silique   Flo: Flower   Cau: Cauline leaf   St: Stem   R: Root
